# First-in-Human Phase I Trial to Assess the Safety and Immunogenicity of an Orf Virus-Based COVID-19 Vaccine Booster

**DOI:** 10.3390/vaccines12111288

**Published:** 2024-11-18

**Authors:** Meral Esen, Johanna Fischer-Herr, Julian Justin Gabor, Johanna Marika Gaile, Wim Alexander Fleischmann, Geerten Willem Smeenk, Roberta Allgayer de Moraes, Sabine Bélard, Carlos Lamsfus Calle, Tamirat Gebru Woldearegai, Diane Egger-Adam, Verena Haug, Carina Metz, Alena Reguzova, Markus W. Löffler, Baiba Balode, Lars C. Matthies, Michael Ramharter, Ralf Amann, Peter G. Kremsner

**Affiliations:** 1 Institute of Tropical Medicine, University Hospital Tübingen, 72074 Tübingen, Germany; 2 Centre de Recherches Médicales de Lambaréné, Lambaréné BP 242, Gabon; 3 German Center for Infection Research (DZIF), Partner Site Tübingen, 72076 Tübingen, Germany; 4 Center for Tropical Medicine, Bernhard Nocht Institute for Tropical Medicine & I. Dep of Medicine, University Medical Center Hamburg-Eppendorf, 20246 Hamburg, Germany; 5 Bernhard Nocht Center for Clinical Trials (BNCCT), 20359 Hamburg, Germany; 6 German Center for Infection Research (DZIF), Partner Sites Hamburg-Lübeck-Borstel-Riems, Germany; 7 Klinikverbund-Suedwest, Germany; 8 Center for Infectious Diseases, Virology, University Hospital Heidelberg, 69120 Heidelberg, Germany; 9 MediTÜV GmbH & Co. KG, Hannover, Standort Hagen, 44263 Dortmund, Germany; 10 Institute of Immunology, University Hospital Tübingen, 72076 Tübingen, Germany; 11 Institute for Clinical and Experimental Transfusion Medicine, Medical Faculty, University Hospital Tübingen, 72076 Tübingen, Germany

**Keywords:** Orf virus, viral vector, vaccine, SARS-CoV-2, COVID-19, Parapoxvirus, first-in-human, phase 1, safety, immunogenicity

## Abstract

The emergence of SARS-CoV-2 has necessitated the development of versatile vaccines capable of addressing evolving variants. Prime-2-CoV_Beta, a novel Orf virus-based COVID-19 vaccine, was developed to express the SARS-CoV-2 spike and nucleocapsid antigens. This first-in-human, phase I, dose-finding clinical trial was conducted to assess the safety, reactogenicity, and immunogenicity of Prime-2-CoV_Beta as a booster in healthy adults. From June 2022 to June 2023, 60 participants in Germany received varying doses of Prime-2-CoV_Beta. The study demonstrated a favorable safety profile, with no serious adverse events (AEs) reported. All AEs were mild (107) or moderate (10), with the most common symptoms being pain at the injection site, fatigue, and headache. Immunogenicity assessments revealed robust vaccine-induced antigen-specific immune responses. High doses notably elicited significant increases in antibodies against the spike and nucleocapsid proteins as well as neutralizing antibodies against SARS-CoV-2 and its variants. Additionally, the vaccine did not induce ORFV-neutralizing antibodies, indicating the potential for repeated administration. In conclusion, Prime-2-CoV_Beta was safe, well tolerated, and immunogenic, demonstrating potential as a broadly protective vaccine against SARS-CoV-2 and its variants. These promising results support further evaluation of higher doses and additional studies to confirm efficacy and long-term protection. This trial was registered at ClinicalTrials, NCT05389319.

## 1. Introduction

The emergence of severe acute respiratory syndrome coronavirus 2 (SARS-CoV-2), which causes COVID-19, led to a global pandemic with devastating impacts, including nearly 7 million deaths worldwide. As of July 2024, over 775 million COVID-19 infections have been reported [1], despite the administration of more than 13 billion vaccine doses [1,2]. Vaccination has been a cornerstone in the fight against COVID-19, significantly reducing infection rates, severity of clinical disease, and mortality. Currently, over 70.6% of the world’s population has received at least one dose of a COVID-19 vaccine [2].

Despite the official end of the pandemic as a public health emergency of international concern in May 2023, COVID-19 continues to cause morbidity and mortality. The dynamic nature of SARS-CoV-2 has led to the identification of multiple variants of concern (VOC) and variants of interest (VOI), including Alpha (B.1.1.7), Beta (B.1.351), Gamma (P.1), Delta (B.1.617.2), and various Omicron sublineages [3,4]. The recent Omicron JN.1 variant, first detected in August 2023, is predicted to be replaced by the KP.3 and KP.3.1.1 variants as the most prevalent variants, underscoring the ongoing evolution of the virus [3,5]. This continual emergence of variants highlights the need for vaccines that can provide broad protection and the importance of developing versatile vaccine platforms that can be rapidly adapted to address future pathogens.

In response to the ongoing need for effective COVID-19 vaccines, we developed Prime-2-CoV_Beta, a novel multi-antigenic vaccine candidate based on the Orf virus strain D1701-VrV (ORFV), a member of the genus Parapoxvirus. Wild-type Orf viruses primarily affect sheep and goats [6,7] and rarely infect humans. The D1701-VrV strain has been rendered non-pathogenic by deleting several virulence genes [8], making it suitable for expressing multiple antigens through predefined insertion sites [9]. A unique advantage of ORFV-based vaccines is their transient vector-specific immunity, preventing the formation of long-lasting vector-specific neutralizing antibodies and enabling effective revaccination [10,11,12]. Preclinical models have demonstrated that ORFV-based vaccines induce robust and long-lasting immune responses, providing protection against various viral infections [12,13,14,15,16,17]. Their favorable safety profile, marked by an inability to replicate in vivo [8], the lack of systemic spread [18,19], and non-virulence even in immunocompromised sheep, justifies their classification under the lowest biosafety level (Category 1) [8]. Additionally, ORFV boasts exceptional thermal stability, remaining stable at 25 °C for up to four weeks and enduring storage at 4 °C or −20 °C for at least two years [20].

Capitalizing on these advantages, Prime-2-CoV_Beta was engineered to express a stabilized form of the full-length SARS-CoV-2 spike protein [21], including the receptor binding domain from the Beta variant, along with the nucleocapsid protein from the original Wuhan Hu-1 strain [22]. The highly conserved nature of the nucleocapsid protein makes it a promising target for the next generation of broadly protective vaccines. This dual-antigen approach has already been incorporated into clinical vaccine candidates [23] (Clinical Trials: NCT04732468; NCT04546841; NCT04639466; NCT04977024; and NCT05370040). Prime-2-CoV_Beta, along with its variant-specific derivatives, has demonstrated the ability to elicit strong humoral and cellular immune responses across different species and has provided protection against SARS-CoV-2 challenge in hamsters and non-human primates [22,24].

In this initial application of the ORFV platform for human use, we embarked on a phase I clinical trial designed to assess the safety, tolerability, and immunogenicity of Prime-2-CoV_Beta as a booster vaccination. Here, we report an interim analysis of a phase I clinical trial dose-escalation study, which seeks to determine the optimal dosing regimen for Prime-2-CoV_Beta. Preliminary findings indicate that the vaccine induces antigen-specific immune responses, resulting in increased and broadened humoral immunity to SARS-CoV-2 and its variants. It further exhibits a high safety profile, and notably, no ORFV-neutralizing antibodies were detected. Based on these encouraging results, we plan to further escalate the dose, as the current highest dose administered has been well-tolerated, suggesting that immunogenicity could be further enhanced at higher dose levels without compromising safety.

## 2. Materials and Methods

### 2.1. Study Design and Participants

To assess the safety and immunogenicity of the multi-antigenic ORFV-based Prime-2-CoV_Beta vaccine candidate, a phase I, multi-center, sequential dose-escalation, open-label clinical trial was conducted. The study sites were the Institute of Tropical Medicine, Internal Medicine VII of the University Hospital of Tübingen (TUE site), and the Bernhardt-Nocht-Institute in Hamburg, Bernhard Nocht Center for Clinical Trials (BNCCT) (HH site), Germany. Healthy participants aged 18-55 years who were fully immunized with at least three doses of any licensed mRNA COVID-19 vaccine (BNT162b2 (Biontech/Pfizer) or mRNA-1273 (Moderna)) were recruited to receive the Prime-2-CoV_Beta booster vaccine.

Following written informed consent, all participants underwent physical examination, hematological and biochemical screening, and were tested for current infections with SARS-CoV-2, human immunodeficiency virus, and hepatitis B and C viruses. Detailed inclusion and exclusion criteria are provided in the study protocol (Supplementary Materials).

### 2.2. Study Cohorts and Dosing

Participants were divided into five cohorts of 12 volunteers each. Cohort 1 received a dose of 3 × 10^4^ PFU, cohort 2 received 3 × 10^5^ PFU, cohort 3 received 3 × 10^6^ PFU, cohort 4 received 1.5 × 10^7^ PFU, and cohort 5 received 3 × 10^7^ PFU. The vaccine (1.0 mL) was administered intramuscularly (i.m.) into the deltoid muscle of the non-dominant arm on Day 1.

### 2.3. Sentinel Participant Protocol

Each cohort began with the vaccination of a sentinel participant, who was observed for at least 48 h post-vaccination, including a minimum four-hour stay at the study center. Follow-up included telephone contacts on the day after vaccination and at the end of the 48 h period to report solicited and unsolicited AEs recorded in a diary until Day 7. If no safety issues arose after 48 h, the next two participants were vaccinated with an interval of at least four hours between each. Upon observing no safety concerns, the subsequent four participants were vaccinated, followed by an additional 48 h observation period. If safety was confirmed, the final five participants were vaccinated with at least a 30 min interval between each participant.

### 2.4. Dose Escalation

Post-vaccination, each participant stayed at the study center for at least four hours for observation. After the last participant in each cohort completed seven days of follow-up, all safety data, including full cardiac assessments and complete safety laboratory results, were reviewed by an independent dose escalation committee (DEC). The DEC provided recommendations on whether to proceed with dose escalation.

### 2.5. Adverse Events Monitoring

Solicited local AEs (pain at the injection site, redness, induration, and swelling) and systemic AEs (fever, fatigue, headache, chills, vomiting, nausea, diarrhea, new or worsened muscle pain, and new or worsened joint pain) were recorded for the first seven days using a structured online diary. Unsolicited AEs, treatment-emergent AEs (TEAEs), serious AEs (SAEs), and AEs of special interest (AESIs) were monitored throughout the study.

### 2.6. Follow-Up Schedule

Follow-up visits were scheduled on Days 4, 8, 15, and 29, and at months 3 and 6 (±14 days). Days 2 and 3 involved telephone calls to review AEs recorded in the diaries. Any unscheduled visits were documented accordingly.

### 2.7. Blood Sample Collection

Blood samples were collected at baseline before immunization (Day 1), Day 8, Day 15, Day 28, Month 3, and Month 6 to monitor blood values for safety and analyze the immunogenicity of the vaccine candidate, as well as anti-vector immunity.

### 2.8. Outcomes

The primary objective was to assess the safety and tolerability of Prime-2-CoV_Beta administered as a booster vaccine by analyzing the proportion of participants with solicited local AEs and solicited systemic AEs during the first 7 days following vaccination. Additionally, SAEs and unsolicited TEAEs were assessed throughout the study. Causality to the study interventions was graded by investigators as unrelated or related. The severity of AEs was graded as mild (grade 1), moderate (grade 2), severe (grade 3), or life-threatening (grade 4) according to the common terminology criteria for adverse events version 4.0. The secondary objectives were to assess the immune responses induced by Prime-2-CoV_Beta based on neutralizing antibodies against the ancient SARS-CoV-2 and binding IgG antibody titers against the receptor-binding domain (RBD) of the spike protein. Further exploratory endpoints included binding IgG antibody responses to the spike protein and the nucleocapsid protein of SARS-CoV-2, neutralizing antibodies against Beta, Delta, and Omicron variants of SARS-CoV-2, induction of T cell responses to the spike protein and nucleocapsid protein, or the immune response to the ORFV vector backbone.

### 2.9. Immunological Assays

Serum IgG antibodies against the Wuhan S1 subunit, RBD, and the Beta variant RBD-domain of the spike protein and the nucleocapsid protein were quantified using a validated in-house ELISA specifically targeting the nucleocapsid protein of SARS-CoV-2 performed by VisMederi Srl (Siena, Italy). Briefly, 96-well microplates (Thermo Fisher, 442404, Hampton, NH, USA) were coated overnight at 4 °C with 1 µg/mL of recombinant S1 (eEnzyme, SCV2-S1-150P, Gaithersburg, MD, USA), RBD (Sino Biological, 40592-V08H, Beijing, China), nucleocapsid (Sino Biological, 40588-V08B), and RBD-Beta (Sino Biological, 40592-V08H85). Plates were then washed with TBS-0.05% Tween 20 (Thermo Fisher, 28360) and blocked with TBS containing 5% non-fat dry milk (Euroclone, APA08300500, Pero, Italy) for 1 h at 37 °C. Serially 2-fold diluted samples, standards, and controls were added and incubated for 1 h at 37 °C, followed by washing and incubation with a goat anti-human IgG-Fc HRP-conjugated antibody (Bethyl Laboratories, A80-104P, Montgomery, TX, USA) at 37 °C for 30 min. Plates were then washed and developed with 3,3′,5,5′-tetramethylbenzidine substrate (Sigma Aldrich, T0440, St. Louis, MO, USA) in the dark at room temperature for 20 min. The reaction was stopped with 0.18 N sulfuric acid (Fisher Scientific, 10080210, Hampton, NH, USA) and read at 450 nm on a SpectraMax plate reader (Molecular Devices, San Jose, CA, USA). The lower limit of quantification (LLOQ) was defined as three times the average optical density (OD) values from blank wells and set to 800 (S1, RBD, RBD-Beta) and 100 (nucleocapsid). Samples below the LLOQ were assigned a value of 0.5*LLOQ, while those above the upper limit of quantitation were pre-diluted before testing.

### 2.10. Neutralizing Antibodies

Neutralizing antibodies against the ancient SARS-CoV-2 variant Wuhan-Hu-1 (Victoria/01/2020) and subsequent VOCs Beta (B.1.351), Delta (B.1.617.2), and Omicron (BA.5) were measured using the validated microneutralization assay (MN-CPE). The MN titer (MNt) is defined as the reciprocal of the highest sample dilution that protects the cell monolayer from cytopathic effect (CPE). Experiments were conducted by VisMederi under BSL3 conditions. SARS-CoV-2 strains were sourced from the CEPI laboratory network, the European Virus Archive GLOBAL (EVAG), Swab isolation, and Rega Institute. Serum samples were heat-inactivated, serially 2-fold diluted in complete DMEM with 2% FBS, and incubated with 25 TCID50 of SARS-CoV-2 variants. VeroE6 cells (ATCC) were infected with virus-serum mixtures and incubated for 3 days for Wuhan and 4 days for Beta, Delta, and Omicron variants. CPE was evaluated, and the highest serum dilution that protected more than 50% of cells was recorded as the neutralization titer. The LLOQ was set to 160. Samples below the LLOQ were assigned a value of 0.5*LLOQ.

### 2.11. ORFV Binding and Neutralizing Antibodies

The ORFV D1701-VrV virus was propagated as previously described [25]. Serum IgG antibodies against ORFV were quantified using an in-house ELISA with 96-well microplates coated with ORFV at 10^7^ PFU/mL overnight at 4 °C. Serial 2-fold dilutions of study participant sera were prepared starting from 1:10 and detected with HRP-conjugated goat anti-IgG-Fc antibody (Bethyl Laboratories, A80-104P) at 1:100,000 dilution. Sera with titers above three times the average plate blank (LLOQ) were considered positive. The MNA was used to measure ORFV-specific neutralizing antibodies. ORFV at 5 × 10^2^ TCID50/mL was preincubated with serum dilutions or controls, and MDCK cells (1.5 × 10^5^ cells/mL) were added. After incubation, CPE was evaluated, and neutralization titers were defined as the highest dilution protecting 50% of cells. If no neutralization was observed, Nt < 10 was assumed and reported as 7.1.

### 2.12. Vaccine Candidate

Prime-2-CoV_Beta is based on the ORFV strain D1701-VrV that encodes the nucleocapsid protein as well as a modified version of the spike protein of SARS-CoV-2 as described elsewhere [22]. The drug substance was produced by ABL Europe SAS, Lyon, France, and the drug product was filled and labeled by Novalabs, Leicester, UK, both under GMP and re-introduced to the EU by PRONAV Clinical Limited, Sligo, Ireland. The vaccine was stored frozen at −20 °C (±10 °C) and reconstituted in natrium chloride solution at each site. Formulated vaccines were administered within 30 min after preparation.

### 2.13. Statistical Analysis

This exploratory phase I clinical trial aimed to detect dose-dependent differences in AEs and vaccine-specific antibodies. The sample size was considered sufficient to evaluate the study objectives. Analyses of demographic characteristics and local and systemic adverse events were performed using descriptive methods. For analysis of immunological assays, data were processed and analyzed with GraphPad Prism 10.3.1 software (GraphPad, San Diego, CA, USA). P-values for pairwise differences between time points within each dose group were calculated using the Wilcoxon signed-rank test, which was performed only if the Friedman test showed significance (*p*-value ≤ 0.05).

## 3. Results

**Figure 1:**
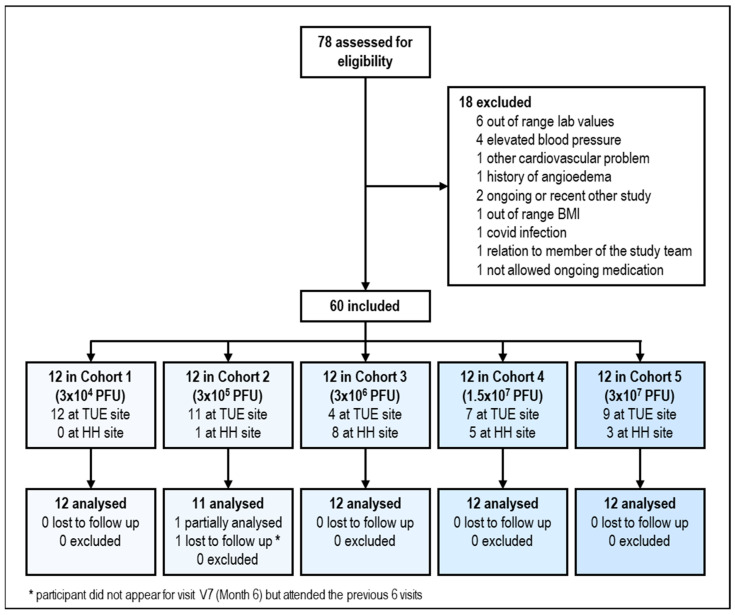
Enrollment flow diagram. BMI = body mass index. PFU= plaque-forming units. TUE site = Institute of Tropical Medicine, University Hospital Tübingen. HH site = Bernhard Nocht Center for Clinical Trials, Hamburg.

From June 2022 to November 2022, 78 volunteers were screened for eligibility. Sixty individuals were enrolled in the study across two sites: 43 at the Clinical Trial Platform of the Institute of Tropical Medicine in Tübingen (TUE site) and 17 at the Bernhard Nocht Center for Clinical Trials in Hamburg (HH site). The participants were allocated into five groups with 12 participants each. All but one volunteer from cohort 2 completed the study (Figure 1). Of the participants, 32 (53.3%) were female and 28 (46.7%) were male. Demographic characteristics were generally balanced across dose cohorts, with no significant differences between the study cohorts or the two respective centers (Table 1).

All participants were fully immunized against COVID-19, with 59 having received three vaccinations and one participant having received five vaccinations (two of which were non-licensed mRNA vaccine candidates (CVnCoV, CureVac)). Of these, 50 were vaccinated exclusively with BNT162b2 (BioNTech/Pfizer), 1 was fully immunized with mRNA-1273 (Moderna), 8 received a combination of BNT162b2 and mRNA-1273, and 1 individual was vaccinated with the vaccine candidate CVnCoV followed by three doses of BNT162b2 (cohort 3). While 10% of the participants reported a history of COVID-19 infection prior to booster immunization (Table 1), 72% exhibited baseline anti-nucleocapsid antibody titers that exceeded the detection limit by more than 3-fold (Figure 4D), highlighting a notable discrepancy.

**Figure 2:**
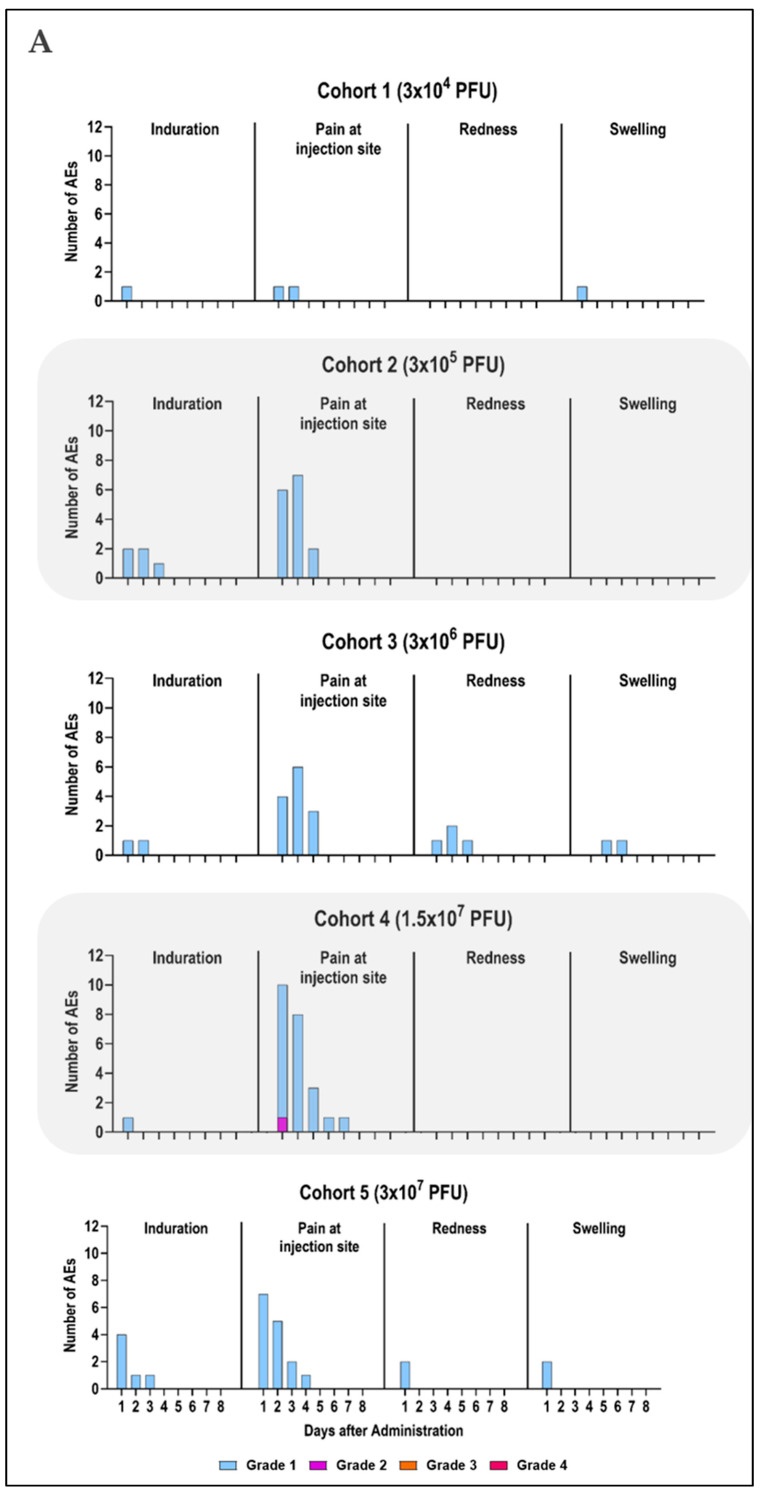

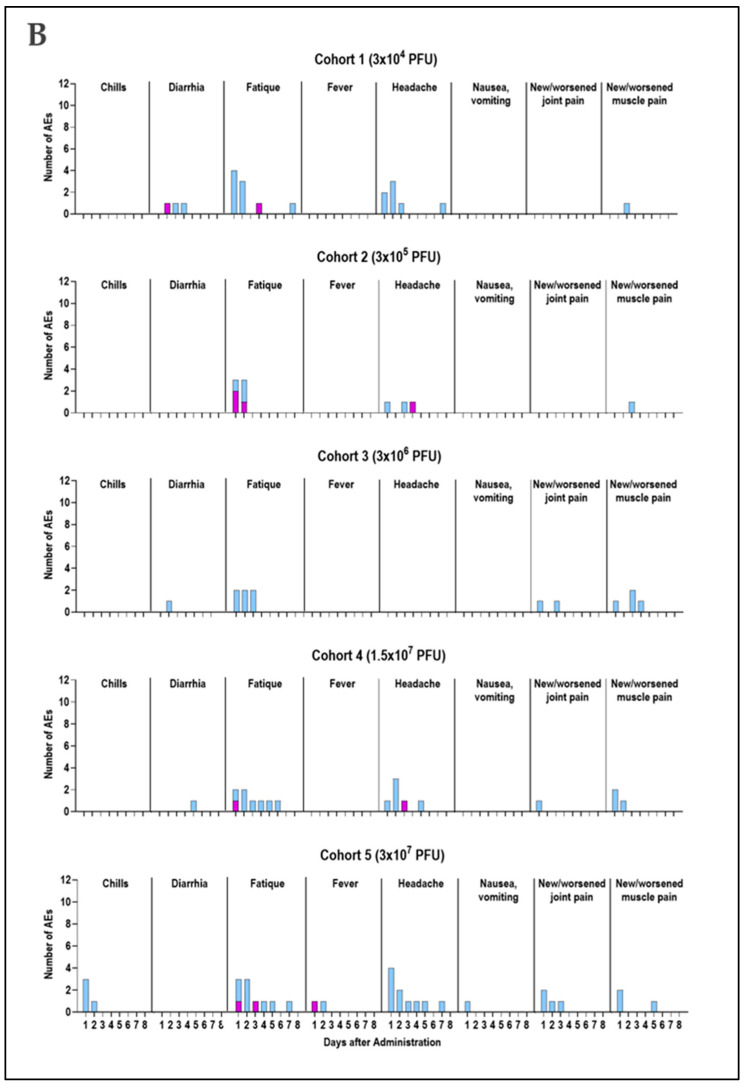
Incidence of solicited local (**A**) and systemic (**B**) adverse events within the first 7 days following Prime-2-CoV_Beta vaccination. Day 1 represents the day of vaccination. AEs were categorized as mild (grade 1), moderate (grade 2), severe (grade 3), and life-threatening (grade 4). All AEs reported during this 7-day period are recorded.

**Figure 3:**
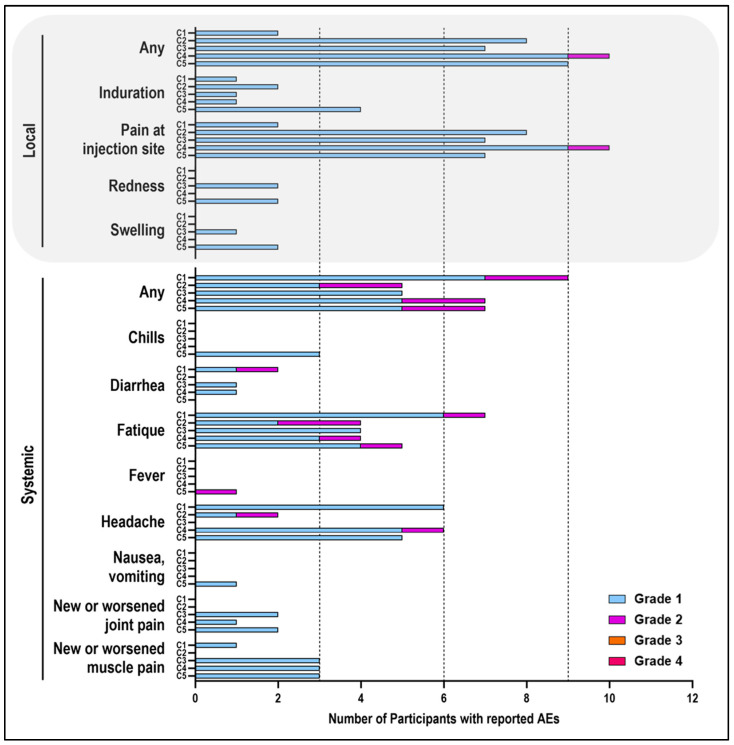
Summary of solicited local and systemic adverse events following Prime-2-CoV_Beta vaccination. The number of participants who had reported solicited local and systemic AEs within 7 days post-immunization. AEs were categorized as mild (grade 1), moderate (grade 2), severe (grade 3), and life-threatening (grade 4). Each type of AE is counted only once per participant, and only the most severe occurrence is recorded. C1-C5 denotes the Cohorts 1–5, respectively.

Overall, Prime-2-CoV_Beta was generally safe and well tolerated. Within the first 7 days following immunization, 125 AEs were reported, with 117 AEs considered related to the vaccine (Figure 2). The remaining eight unrelated AEs included three mild and five moderate occurrences. Related AEs were reported by 47 individuals (78.3%), with 36 (=60%) reporting local AE and 33 (=55%) reporting systemic AE. Most of the local AE were of grade 1 (35) with one single report of grade 2 being the exception, whereas systemic AE was of grade 1 in 25 cases and of grade 2 in 8 cases (Table 2, Figure 3). Thirteen study participants (21.7%) did not experience any AEs, with these participants being evenly distributed across cohorts 1, 2, 3, and 5 (3, 4, 3, and 3, respectively). No serious AEs, hospitalization, or deaths attributed to Prime-2-CoV_Beta were reported, and none of the prespecified stopping rules were met.

Most solicited AEs occurred during the first 2 days after vaccination and had a short duration, with a median time to resolution of one day. Pain at the injection site, fatigue, and headache sometimes persisted slightly longer (Figure 2).

The most common solicited AEs within 7 days of any dose were pain at the injection site, reported by 34 participants (56.7%). This was most frequently observed in cohort 4 (10 participants, 83.3%), followed by cohort 2 (8 participants, 66.7%), and cohort 3 (7 participants, 58.3%). Fatigue was reported by 24 participants (40%) and accounted for five of the eight grade 2 systemic AEs. Fatigue was most frequently reported in cohort 1 (seven participants, 58.3%) and in cohort 5 (five participants each, 41.7%). The grade 2 fatigue AEs were reported by participants in cohorts 1, 2, 4, and 5. Headache was reported by 19 of the participants (31.7%), with cohort 1 and cohort 4 reporting the highest number (6 participants each, 50%). The two grade 2 headache AEs were reported by participants in cohort 2 and cohort 4 (one participant each, 8.3%). New or worsened muscle pain was reported by 10 participants (16.7%), and induration by 9 participants (15%). All other AEs were occasionally reported by one to five participants (1.7–8.3%) (Figure 3, Table 2).

In cohort 1, two participants reported local AEs, while nine participants reported systemic AEs. Of the 19 AEs reported, 2 were of grade 2. The most frequent event reported was fatigue (seven participants), followed by headache (six participants), diarrhea, and pain at the injection site (two participants each) (Figure 2 and Figure 3). Similarly, in cohort 2, eight participants reported a total of 16 AEs, including 2 grade 2 events. The most frequent AEs in this cohort were pain at the injection site (eight participants) and fatigue (four participants). In cohort 3, 9 out of 12 individuals experienced a total of 21 AEs, all of which were grade 1, with pain at the injection site (7 participants) and fatigue (4 participants) being the most common. Participants in cohort 4 reported a total of 26 AEs, including 3 grade 2 events. The most frequent AEs in this cohort were pain at the injection site (10 participants), headache (6 participants), and fatigue (4 participants). The highest dose cohort, cohort 5, had 9 out of 12 participants reporting a total of 35 AEs, with 33 being grade 1 and 2 being grade 2 (see Figure 2 and Figure 3). The most frequent AEs in this cohort were pain at the injection site (seven participants), headache (five participants), and fatigue (five participants, one of which was grade 2) (Table 2). Occasional grade 1 and 2 laboratory abnormalities, considered clinically relevant, were observed in cohorts 1, 3, and 5. None of these post-vaccination abnormalities were associated with clinical findings. Twelve clinically relevant changes in laboratory values were judged as AEs by the investigators, but none were related to the vaccine. During the follow-up period, a total of 144 AEs were reported, and 6 were judged to be treatment-associated adverse events (TAEs).

**Figure 4:**
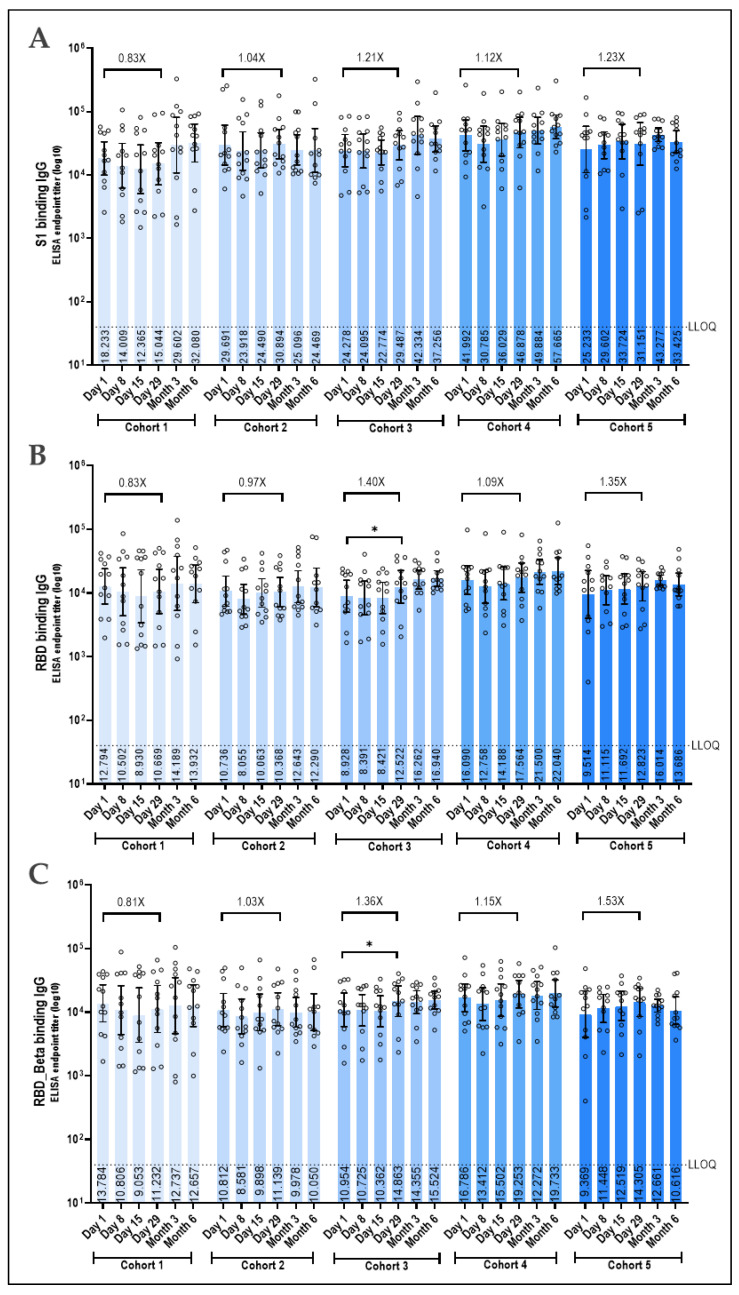

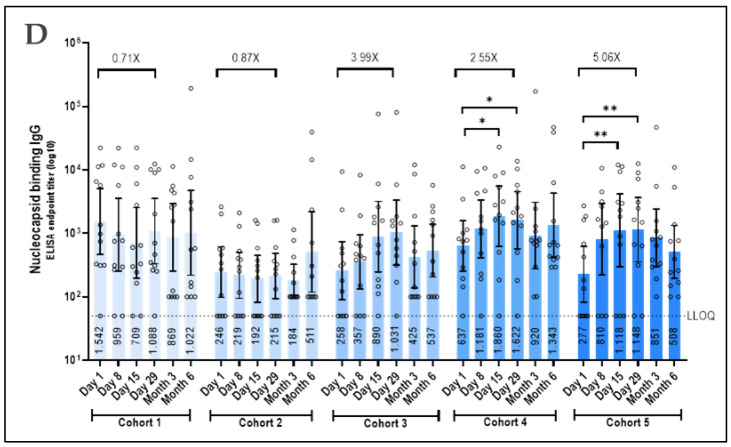
SARS-CoV-2 spike- and nucleocapsid-specific antibody responses following Prime-2-CoV_Beta vaccination. IgG binding antibody levels against the S1 subunit of spike of the ancient SARS-CoV-2 (**A**), the RBD of spike of the ancient SARS-CoV-2 (**B**), the RBD of spike of the SARS-CoV-2 Beta variant (**C**), and of the nucleocapsid of the ancient SARS-CoV-2 (**D**) as measured in serum samples obtained from vaccinated participants at indicated time points by a validated in-house ELISA. Logarithmic values are reported as the geometric mean titer (GMT), and the bars represent the geometric mean with a 95% CI. Fold change from Day 29 to baseline is denoted above the columns. GMT at each time point is indicated in the columns. LLOQ = Lower Limit of Quantification is indicated by the dotted line. For pairwise comparisons of time points within each dose group, the Friedman test was used, followed by the Wilcoxon signed-rank test if the Friedman test was significant (*p*-value ≤ 0.05). * *p* < 0.05; ** *p* < 0.01.

Humoral immune response was measured before and following booster vaccination with Prime-2-CoV_Beta over a period of 6 months. Notably, baseline antibody titers and neutralizing antibody levels on Day 1 against all tested antigens and SARS-CoV-2 variants were highly heterogeneous across the participants. This variability was due to participants having received at least three vaccinations with mRNA-based COVID-19 vaccines and potentially being infected with SARS-CoV-2 or its variants before inclusion in the study. Consequently, baseline titers, especially against the spike S1 subunit, the receptor-binding domain (RBD), the RBD from the SARS-CoV-2 Beta variant (RBD_Beta), and the nucleocapsid protein, were very high across all dose cohorts, with the 1.5 × 10^7^ PFU dose group showing particularly high titers (Figure 4). Antibodies against the S1 subunit and the RBD or RBD_Beta were modestly increased at doses equal to or greater than 3 × 10^6^ PFU, peaking on day 29 post-boost immunization. In the lower dose cohorts, titers either declined by approximately 0.8-fold in the 3 × 10^4^ PFU dose cohort or remained unchanged in the 3 × 10^5^ PFU dose cohort. The highest dose cohort exhibited the most pronounced elevations, with antibodies specific to the RBD of the SARS-CoV-2 Beta sequence slightly more elevated compared to those against the RBD of the original SARS-CoV-2, with a 1.53-fold increase versus a 1.35-fold increase, respectively (Figure 4A–C). Nucleocapsid-specific antibodies were distinctly increased in the three higher dose cohorts 3, 4, and 5, with cohort 5 showing the highest increase at 5.06-fold at Day 29. The two lower dose cohorts did not show enhanced nucleocapsid-specific antibody titers, and titers declined by Day 29 from baseline by 0.71-fold and 0.87-fold in cohorts 1 and 2, respectively, indicating a dose-dependent response for this antigen as well (Figure 4D).

**Figure 5:**
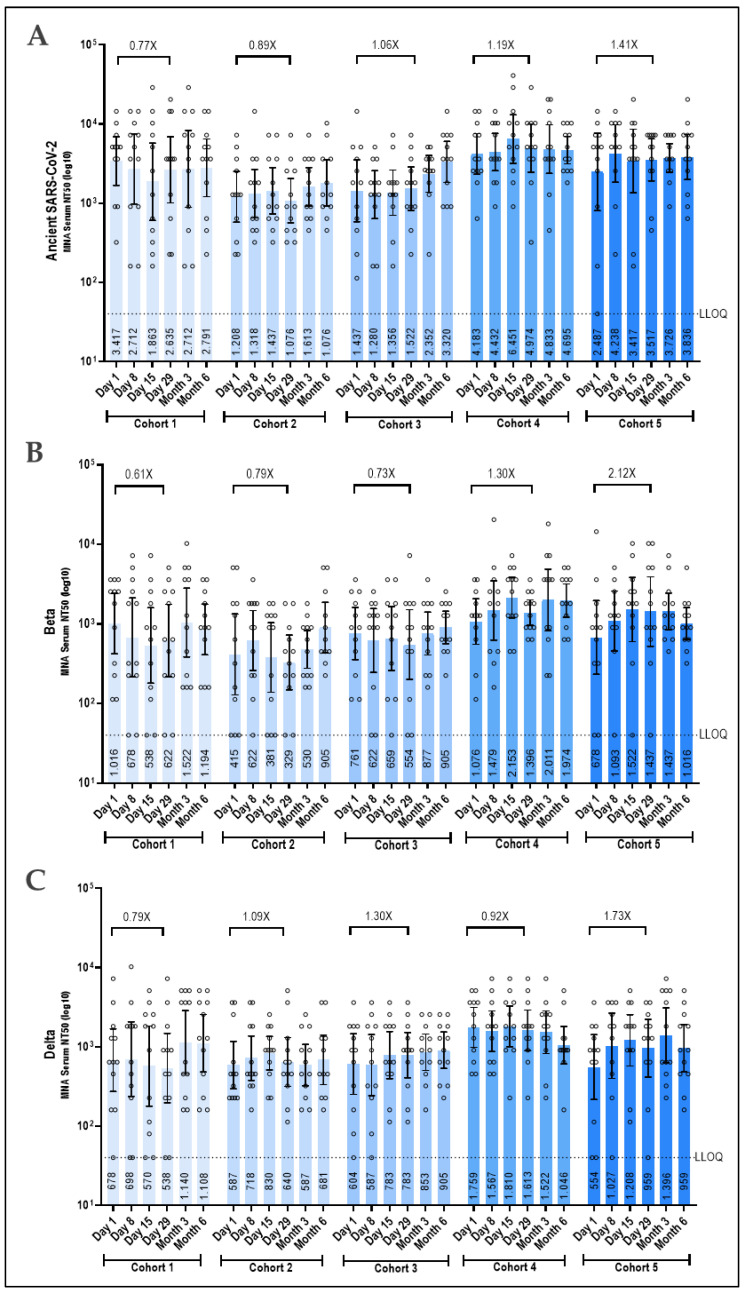

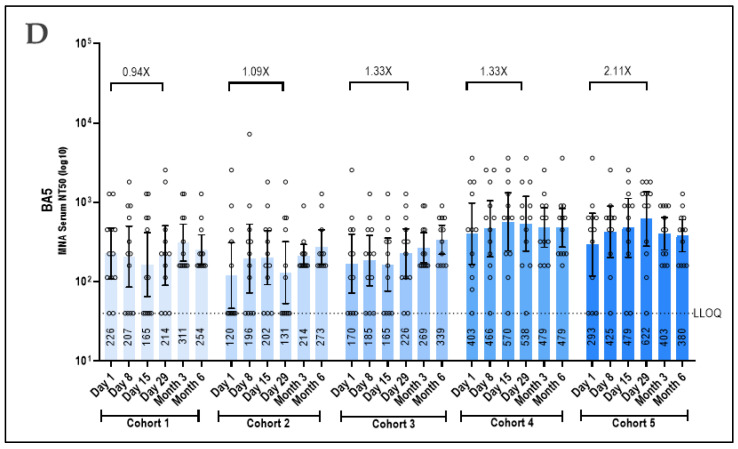
SARS-CoV-2 neutralization response following Prime-2-CoV_Beta vaccination to ancestral SARS-CoV-2, Beta, Delta, and BA.5. Neutralizing antibody levels against the ancient SARS-CoV-2 (**A**), the SARS-CoV-2 Beta variant (**B**), the SARS-CoV-2 Delta variant (**C**), and the SARS-CoV-2 Omicron BA.5 variant (**D**) as measured in serum samples obtained from vaccinated participants at indicated time points by a validated microneutralization assay. Logarithmic values are reported as the GMT, and the bars represent the geometric mean with a 95% CI. Fold change from Day 29 to baseline is denoted above the columns. GMT at each time point is indicated in the columns. LLOQ = lower limit of quantification is indicated by the dotted line. For pairwise comparisons of time points within each dose group, the Friedman test was used, followed by the Wilcoxon signed-rank test if the Friedman test was significant (*p*-value ≤ 0.05).

Neutralizing antibodies against SARS-CoV-2 and its variants increased in a dose-dependent manner, with the highest dose cohort eliciting the greatest increase in SARS-CoV-2 neutralizing serum antibodies. In line with the antibody data, increases in neutralization against the Beta variant were higher than against the original SARS-CoV-2 virus, with a 2.12-fold increase versus a 1.41-fold increase in the highest dose cohorts (Figure 5A,C). Furthermore, neutralization against the BA.5 and Delta variants showed a 2.11-fold and 1.73-fold increase, respectively, indicating broad neutralizing activity induced by the vaccine (Figure 5B,D). The two lowest dose cohorts failed to increase existing neutralizing antibody titers against all tested SARS-CoV-2 variants (Figure 5).

**Figure 6:**
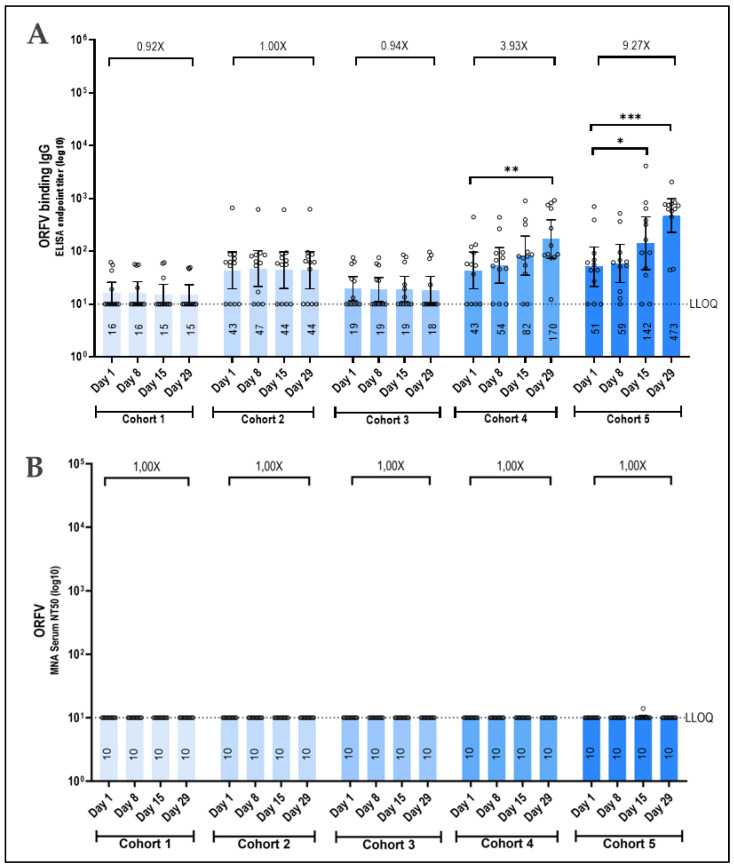
ORFV-specific immune response and ORFV neutralization following Prime-2-CoV_Beta vaccination. IgG binding antibody levels (**A**) and neutralizing antibody levels (**B**) against ORFV strain D1701-VrV as measured in serum samples obtained from vaccinated participants at indicated time points by ELISA or microneutralization assay, respectively. Logarithmic values are reported as the GMT, and the bars represent the geometric mean with a 95% CI. Fold change from Day 29 to baseline is denoted above the columns. GMT at each time point is indicated in the columns. LLOQ = lower limit of quantification is indicated by the dotted line. For pairwise comparisons of time points within each dose group, the Friedman test was used, followed by the Wilcoxon signed-rank test if the Friedman test was significant (*p*-value ≤ 0.05). * *p* < 0.05; ** *p* < 0.01; *** *p* < 0.001.

Antibodies against ORFV were measured to determine the immune response against the viral vector. No responses were measured in the three lowest dose cohorts. However, in the 1.5 × 10^7^ and 3 × 10^7^ PFU cohorts, a dose-dependent response was observed, with titers changing by 3.93-fold and 9.27-fold against baseline, respectively (Figure 6A). The ability to neutralize ORFV was tested in an ORFV-neutralizing antibody assay, but none of the participants induced an ORFV-neutralizing antibody response, regardless of the administered vaccine dose or ORFV binding titers (Figure 6B).

## 4. Discussion

In this interim report, we describe the tolerability, safety, and immunogenicity of the COVID-19 vaccine candidate Prime-2-CoV_Beta, marking the first use of an ORFV-based pharmaceutical in humans. Prime-2-CoV_Beta was designed as a multi-antigenic vector vaccine to express a prefusion-stabilized version of the spike protein, which has been shown to enhance vaccine immunogenicity and efficacy [21], updated with mutations within the RBD of the SARS-CoV-2 Beta variant, in combination with the nucleocapsid protein of the original SARS-CoV-2. Targeting the well-conserved nucleocapsid protein, in addition to the spike protein, has been suggested to potentially improve vaccine efficacy [26,27] by substantially increasing cell-mediated immunity [28] and facilitating cross-reactive T-cell responses [29] or via Fc. Other proposed mechanisms include Fc-dependent antibody functions, such as antibody-dependent cellular phagocytosis/cytotoxicity (ADCP/ADCC) [30,31,32]. This strategy could potentially enable protection independent of the frequently mutating spike protein (14), addressing the ongoing challenge posed by future SARS-CoV-2 variants [33]. Various approaches targeting the nucleocapsid protein have been evaluated preclinically [34,35,36,37,38] and are already in clinical development [23,39,40]. However, the additional benefit of addressing the nucleocapsid has not been unequivocally demonstrated. Antibodies against the N-protein are generally non-neutralizing and may not significantly contribute to protective immunity against SARS-CoV-2. Vaccines targeting the N-protein alone are reported to be ineffective [41] or provide only minimal protection at best [34], and convincing mechanisms of protection involving the N-protein are lacking and remain a subject of ongoing debate within the scientific community.

Seventy-eight subjects were screened for eligibility, and sixty were included in the study, with fifty-nine completing the study and one participant from cohort 2 losing to follow-up (unrelated to adverse events). The participants had received 3-5 mRNA vaccinations prior to the start of the study, with the vast majority having received either only BNT162b2 (50 out of 60) or a combination of BNT162b2 and mRNA-1273 (8 out of 60). The last vaccination was received on average 275 days before the study started. Ten percent of the participants, all of cohort 3 (four participants) or 4 (two participants), reported a previous SARS-CoV-2 infection. Interestingly, the baseline titer against the nucleocapsid protein (anti-N titer) of these previously SARS-CoV-2 infected participants was slightly lower than that of the other participants who not report a previous SARS-CoV-2 infection (GMT of 383 vs. 432). Additionally, the proportion of participants with a >3-fold higher anti-N titer above the lower detection limit was comparable between those with (67%) and without (65%) a reported previous SARS-CoV-2 infection. As none of the vaccines administered prior to the study contained the nucleocapsid, the baseline anti-N titer serves as a correlate of previous infections. This suggests that at least 65% of the participants had likely been infected with SARS-CoV-2 at least once before entering the study and therefore possess a hybrid immunity status. The high discrepancy between reported and detected previous infections was also found in other studies [42,43] and might represent asymptomatic breakthrough infections of vaccinated participants [44], misdiagnosis, or memory lapse.

Overall, a total of 125 local or systemic solicited AEs were reported during the first 7 days after vaccination of Prime-2-CoV_Beta by 47 distinct subjects, with 117 AEs being vaccine-related. One hundred seven were of grade 1, and ten were of grade 2, with no grade 3 or grade 4 events reported. The local and systemic reactions usually occurred within 48 h after immunization and generally resolved within 24 h. A trend towards a higher total incidence of solicited local and systemic AEs was noted for the two highest dose cohorts, although this could not be attributed to single symptoms but rather the total incidence. The most frequent symptoms were pain at the injection site (56.7%), fatigue (40%), and headache (31.7%). The profile of the reported reactions is comparable to other COVID-19 vaccines such as mRNA [45,46] or viral vector-based vaccines [23,47,48], but seemed to be milder and less frequent at the tested doses.

The induction of neutralizing antibodies in response to booster vaccination is a crucial parameter for assessing vaccine efficacy. Although there is no strict correlation of protection, the presence of neutralizing antibodies strongly correlates with protection against COVID-19 infections, thus providing important information on the vaccine’s effectiveness [49,50,51]. This vaccine was developed and tested during the Beta variant’s prevalence. However, the rapid evolution of SARS-CoV-2 has resulted in the emergence of additional COVID-19 variants that have replaced these early strains [3]. Due to cost and time constraints, continuous adjustment of the vaccine was not feasible. To determine if protection against these newer variants could be achieved, we also tested immunity against the Delta and Omicron BA.5 variants. At the highest tested dose, we observed a moderate increase in antibodies against the original virus (1.41-fold). As expected, the increase in antibodies was higher against the Beta variant (2.12-fold). Encouragingly, enhanced neutralization was also observed against the Delta and BA.5 variants (1.73-fold and 2.11-fold, respectively), indicating that not only the pre-existing immunity is boosted—a phenomenon known as “immune imprinting” [52,53]—but also a broader immunity against the new antigenic components is induced.

To contextualize the induced immunity, we refer to published studies, although these offer limited comparability due to differences in factors such as baseline antibody titers, inclusion and exclusion criteria, analytical methods, study populations, and study sites. For instance, a study with the protein-based vaccine NVX-CoV2373 (Novavax) was conducted on participants who, similar to our study, had previously received at least three mRNA vaccinations [42]. In this study, the titers of neutralizing antibodies against the original SARS-CoV-2 virus increased by a factor of 1.75 and against the BA.5 variant by a factor of 1.58. Thus, the increase in neutralizing antibodies against the original virus was higher (1.75-fold vs. 1.41-fold) and against the BA.5 variant lower (1.58-fold vs. 2.11-fold) than observed in our study.

In another study, the mRNA-based vaccine mRNA1273 (Moderna) was also tested on participants who had previously received three mRNA-based vaccinations [43]. Here, the titers against the original SARS-CoV-2 virus and the BA.5 variant increased by factors of 3.11 and 3.78, respectively, representing a significant increase compared to our vaccine candidate or the protein-based vaccine.

In our study, the immune response against the nucleocapsid protein increased by a factor of 5.06 in the highest dose cohort. This higher increase compared to the anti-spike titers can likely be attributed to the significantly lower baseline of the anti-nucleocapsid immune response at the start of the study. Notably, in preclinical studies, we could induce immunogenicity against the nucleocapsid protein only moderately and significantly less strongly than the immunogenicity against the spike protein [22]. In a study with a bivalent modified vaccinia virus Ankara (MVA)-based vaccine encoding both spike and nucleocapsid protein, a dose-dependent increase by a factor of 2–4.5 after a single immunization was observed, which roughly corresponds to the values observed in our trial. Following a subsequent immunization, this value increased by a factor of 15–50 [23].

Albeit a robust induction of humoral immunity against the nucleocapsid protein was shown, demonstrating the added benefit of including the nucleocapsid protein in a phase I study presents several challenges. Currently, there is no established evidence or demonstrated mechanism for this additional benefit. Furthermore, no correlate of protection for SARS-CoV-2 has been identified, although a higher virus-neutralizing antibody (VNA) titer correlates with enhanced protection [51]. Although we investigated T-cell responses in our study, the results were too inconsistent to draw definitive conclusions, and the role of T-cell responses in protection against SARS-CoV-2 remains inadequately understood.

To conclusively demonstrate an additional benefit in a clinical study, a control group receiving the same vaccine without the nucleocapsid protein would be required. The study cohort would need to be sufficiently large to show an enhanced protective effect, either through the prevention of new infections or a milder disease course. Despite these challenges, our studies in animal models have shown that hamsters vaccinated with the vaccine containing the nucleocapsid protein exhibited better protection of the upper respiratory tract compared to those vaccinated with the spike-only vaccine [22]. Furthermore, other research groups have also reported added value when combining the spike and the nucleocapsid antigens in a vaccine [34,37,54].

One of the main drawbacks of viral vector-based vaccines is the pre-existing or immunization-induced immunity against the vector virus, known as anti-vector immunity [55,56,57,58,59]. This immunity results in the neutralization of the viral vector, thereby significantly reducing its efficacy. Reports of recurrent infections with wild-type ORFV in sheep and goats, as well as preclinical studies with ORFV-based vaccine candidates, suggest that no long-lasting immunity against ORFV is established [10,11]. Although reports of ORFV infections are very rare and the likelihood of pre-existing anti-ORFV immunity seems negligible, the lack of anti-ORFV neutralizing antibodies presents a significant advantage. This would allow for repeated use and administration of ORFV without impairment by anti-vector immunity, offering a distinct advantage over other vector technologies and making the ORFV platform a sustainable option for addressing various diseases.

In this study, the induction of anti-ORFV antibodies was investigated to determine whether these antibodies can neutralize ORFV. A dose-dependent induction of ORFV-specific antibodies was noticed, with all subjects in the highest dose cohort seroconverting. However, these antibodies were unable to neutralize ORFV. It is anticipated that higher doses or repeated administration of ORFV would generate higher ORFV-specific antibody titers. Future research is needed to determine if ORFV-neutralization remains absent even at these higher antibody titers, corroborating findings from animal studies [12,22].

Our study has several limitations. Considering ethical aspects, we decided to include only participants who had been vaccinated at least three times with an approved COVID-19 vaccine, as this was the recommendation of the competent health authority in Germany at the time. To ensure a homogeneous participant population, we also restricted study inclusion to those vaccinated exclusively with mRNA-based COVID-19 vaccines, as this represented the largest cohort within society. Individuals vaccinated with other vaccine technologies were excluded, which means the study population does not fully reflect the broader societal composition. Additionally, previous COVID-19 infections were not considered in the inclusion criteria, which may have contributed to the very high baseline levels of antibodies against the spike protein at the start of the study. We also noted considerable variability in these immune responses. While the cohort size of 12 participants was appropriate for a phase I study, the high variability in immune responses limited the ability to perform meaningful statistical evaluations.

Another limitation of the study was that, for ethical reasons, no placebo group was included, nor was there a comparison group with an already approved vaccine, which significantly complicates the assessment and evaluation of the vaccine’s immunogenicity and efficacy. Furthermore, the study design was open-label and non-randomized, and the participants were not ethnically diverse. Neither older nor younger participants were included, nor were participants with pre-existing conditions included.

The additional benefit of immunization against the nucleocapsid protein could not be demonstrated by this study design and most likely can only be addressed in large studies allowing for assessment of COVID-19 incidence as a readout, as mentioned before.

Since this was the first use of an ORFV-based vaccine in humans, investigations into the shedding of the vector after its administration would be very interesting, although detailed studies in animals suggest this is a highly unlikely occurrence [8].

Ultimately, the optimal dose was very likely not achieved yet, as the side effects were still described as mild even in the highest dose cohort, and immune responses increased in a dose-dependent manner at higher doses. Considering the insights and results gained from this study, as well as the described limitations, we plan to continue the study with higher doses and a slightly modified study design. We aim to gradually increase the dose to 1.2x10^8^ PFU and include a cohort that allows participation regardless of previous COVID-19 vaccinations. Additionally, we plan to collect stool, urine, sputum, and blood samples to investigate potential ORFV shedding.

## 5. Conclusions

We demonstrated that Prime-2-CoV_Beta, when used as a booster vaccine in healthy subjects who had previously received at least three doses of an mRNA vaccine, was very well tolerated at the tested doses. Furthermore, at higher doses, the production of antibodies against the two encoded antigens, spike and nucleocapsid, was stimulated, and cross-reactive immunity against SARS-CoV-2 variants was induced. At the two highest doses, the production of ORFV-specific antibodies was induced, although these were not capable of neutralizing ORFV. In summary, these initial results are very promising and encourage further evaluation of this vaccine technology at higher doses.

## 6. Patents

R.A. and A.R. are inventors of patents related to ORFV, including a patent application of Prime-2-CoV_Beta (EP23730776).

## Figures and Tables

**Table 1:** Demographic characteristics. Data are mean (range) or n (%) or mean and standard deviation (SD). Solicited AEs from cohort 1 (3 × 10^4^ PFU/dose), cohort 2 (3 × 10^5^ PFU/dose), cohort 3 (3 × 10^6^ PFU/dose), cohort 4 (1.5 × 10^7^ PFU/dose), and cohort 5 (3 × 10^7^ PFU/dose) were reported within the first 7 days after vaccination. * The participant received two doses of an investigational COVID-19 vaccine (CVnCoV, Curevac) before receiving three doses of BNT162b2.

Demographic Characteristics		All Cohorts (n = 60)	Cohort 1 (n = 12)	Cohort 2 (n = 12)	Cohort 3 (n =1 2)	Cohort 4 (n = 12)	Cohort 5 (n = 12)
**Age, years**		28.4 (19–53)	26.8 (20–39)	26.2 (21–38)	33.3 (20–53)	27.7 (19–41)	27.9 (19–46)
**Gender**	Female	32 (53.3%)	5 (41.7%)	8 (66.7%)	6 (50%)	7 (58.3%)	6 (50%)
	Male	28 (46.7%)	7 (58.3%)	4 (33.3%)	6 (50%)	5 (41.7%)	6 (50%)
**Body-mass index, kg/m^2^**		23.0 (18.7–29.6)	22.8 (18.9–28.1)	23.1 (20.7–27.2)	22.9 (19.6–25.2)	23.7 (18.7–29.6)	22.5 (18.9–29.4)
**Race**	Caucasian	57 (95%)	11 (91.7%)	11 (91.7%)	12 (100%)	12 (100%)	11 (91.7%)
	Asian	2 (3.3%)	0	1 (8.3%)	0	0	1 (8.3%)
	Other	1 (1.7%)	1 (8.3%)	0	0	0	0
**Previous COVID-19 vaccine doses**	3x BNT162b2 only	50 (83.3%)	12 (100%)	11 (91.7%)	9 (75%)	10 (83.3%)	8 (66.7%)
	3x mRNA-1273 only	1 (1.6%)	0	0	1 (8.3%)	0	0
	Combination of BNT162b2 + mRNA-1273	8 (13.3%)	0	1 (8.3%)	2 (16.7%)	1 (8.3%)	4 (33.3%)
	Other *	1 (1.6%)	0	0	0	1 (8.3%)	0
**Time between previous COVID-19 vaccine and first study dose, days**	Mean (SD)	275 (61)	212 (36)	233 (26)	285 (62)	310 (35)	333 (39)
**Pre-Infections with COVID-19 (reported)**		6 (10%)	0	0	4 (33.3%)	2 (16.7%)	0

**Table 2:** Overall summary of adverse events following Prime-2-CoV_Beta vaccination. Data are n (%). Solicited AEs from cohort 1 (3 × 10^4^ PFU/dose), cohort 2 (3 × 10^5^ PFU/dose), cohort 3 (3 × 10^6^ PFU/dose), cohort 4 (1.5 × 10^7^ PFU/dose), and cohort 5 (3 × 10^7^ PFU/dose) were reported within the first 7 days after vaccination. Each type of AE is counted only once per participant, and only the most severe occurrence is recorded.

		All Cohorts (n = 60)	Cohort 1 (n = 12)	Cohort2 (n = 12)	Cohort3 (n = 12)	Cohort 4 (n = 12)	Cohort 5 (n = 12)
**Local**							
**Any**	Total	36 (60%)	2 (16.7%)	8 (66.7%)	7 (58.3%)	10 (83.3%)	9 (75%)
	Grade 1	35 (58.3%)	2 (16.7%)	8 (66.7%)	7 (58.3%)	9 (75%)	9 (75%)
	Grade 2	1 (1.7%)	0	0	0	1 (8.3%)	0
**Induration**	Total	9 (15%)	1 (8.3%)	2 (16.7%)	1 (8.3%)	1 (8.3%)	4 (33.3%)
	Grade 1	9 (15%)	1 (8.3%)	2 (16.7%)	1 (8.3%)	1 (8.3%)	4 (33.3%)
	Grade 2	0	0	0	0	0	0
**Pain at injection site**	Total	34 (56.7%)	2 (16.7%)	8 (66.7%)	7 (58.3%)	10 (83.3%)	7 (58.3%)
	Grade 1	33 (55%)	2 (16.7%)	8 (66.7%)	7 (58.3%)	9 (75%)	7 (58.3%)
	Grade 2	1 (1.7%)	0	0	0	1 (8.3%)	0
**Redness**	Total	4 (6.7%)	0	0	2 (16.7%)	0	2 (16.7%)
	Grade 1	4 (6.7%)	0	0	2 (16.7%)	0	2 (16.7%)
	Grade 2	0	0	0	0	0	0
**Swelling**	Total	3 (5%)	0	0	1 (8.3%)	0	2 (16.7%)
	Grade 1	3 (5%)	0	0	1 (8.3%)	0	2 (16.7%)
	Grade 2	0	0	0	0	0	0
**Systemic**							
**Any**	Total	33 (55%)	9 (75%)	5 (41.7%)	5 (41.7%)	7 (58.3%)	7 (58.3%)
	Grade 1	25 (41.7%)	7 (58.3%)	3 (25%)	5 (41.7%)	5 (41.7%)	5 (41.7)
	Grade 2	8 (13.3%)	2 (16.7%)	2 (16.7%)	0	2 (16.7%)	2 (16.7%)
**Chills**	Total	3 (5%)	0	0	0	0	3 (25%)
	Grade 1	3 (5%)	0	0	0	0	3 (25%)
	Grade 2	0	0	0	0	0	0
**Diarrhea**	Total	4 (6.7%)	2 (16.7%)	0	1 (8.3%)	1 (8.3%)	0
	Grade 1	3 (5%)	1 (8.3%)	0	1 (8.3%)	1 (8.3%)	0
	Grade 2	1 (1.7%)	1 (8.3%)	0	0	0	0
**Fatigue**	Total	24 (40%)	7 (58.3%)	4 (33.3%)	4 (33.3%)	4 (33.3%)	5 (41.7%)
	Grade 1	19 (31.7%)	6 (50%)	2 (16.7%)	4 (33.3%)	3 (25%)	4 (33.3%)
	Grade 2	5 (8.3%)	1 (8.3%)	2 (16.7%)	0	1 (8.3%)	1 (8.3%)
**Fever**	Total	1 (1.7%)	0	0	0	0	1 (8.3%)
	Grade 1	0	0	0	0	0	0
	Grade 2	1 (1.7%)	0	0	0	0	1 (8.3%)
**Headache**	Total	19 (31.7%)	6 (50%)	2 (16.7%)	0	6 (50%)	5 (41.7%)
	Grade 1	17 (28.3%)	6 (50%)	1 (8.3%)	0	5 (41.7%)	5 (41.7%)
	Grade 2	2 (3.3%)	0	1 (8.3%)	0	1 (8.3%)	0
**Nausea, vomiting**	Total	1 (1.7%)	0	0	0	0	1 (8.3%)
	Grade 1	1 (1.7%)	0	0	0	0	1 (8.3%)
	Grade 2	0	0	0	0	0	0
**New or worsened joint pain**	Total	5 (8.3%)	0	0	2 (16.7%)	1 (8.3%)	2 (16.7%)
	Grade 1	5 (8.3%)	0	0	2 (16.7%)	1 (8.3%)	2 (16.7%)
	Grade 2	0	0	0	0	0	0
**New or worsened muscle pain**	Total	10 (16.7%)	1 (8.3%)	0	3 (25%)	3 (25%)	3 (25%)
	Grade 1	10 (16.7%)	1 (8.3%)	0	3 (25%)	3 (25%)	3 (25%)
	Grade 2	0	0	0	0	0	0

## Data Availability

The protocol of the study is provided in the Supplementary Materials. Data sharing of the individual de-identified participant that underlies the results reported in this study will be made available upon request.

## Supplementary Materials

The study protocol can be downloaded at: https://www.mdpi.com/article/10.3390/vaccines12111288/s1.
