# Supplementary material for: First-in-Human Phase I Trial to Assess the Safety and Immunogenicity of an Orf Virus-Based COVID-19 Vaccine Booster
Source: Vaccines (Basel). 2024 Nov 18;12(11):1288. doi: 10.3390/vaccines12111288 (PMC11599021; doi:10.3390/vaccines12111288)
Supplement: Supplementary file 1 [file vaccines-12-01288-s001.zip › vaccines-3266366-supplementary.pdf]

STUDY PROTOCOL

A phase Ia, dose-finding study to assess the safety and immunogenicity of an Orf virus-based COVID-19 vaccine booster (Prime-2-CoV_Beta) in healthy adults

EudraCT No: 2021-001191-42

Protocol no.:	PRiME2_21_1
Sponsor:	University Hospital Tübingen<br>[redacted]<br>Geissweg 3<br>72076 Tübingen, Germany
Sponsor's signatory:	[redacted]
Chief investigator:	[redacted]<br>University Hospital Tübingen<br>Institute of Tropical Medicine,<br>University of Tübingen,<br>Wilhelmstr. 27<br>72074 Tübingen, Germany
Protocol version and date:	Final 5.0, 15-Jul-2022
Previous protocol version:	Final 4.0, 17-May-2022<br>Final 3.0, 02-May-2022<br>Final 2.0, 31-Mar-2022<br>Final 1.0, 07-Dec-2021

This study protocol must be kept strictly confidential. Disclosure of the contents (in whole or part) to third parties is permissible only with written consent of University Hospital Tübingen.

Revision chronology

- Original version: Final 1.0. 07-Dec-2021.
- Version 2.0, dated 31-Mar-2022
- Version 3.0, dated 02-May-2022

  Version 2.0 was erroneously submitted to the Paul-Ehrlich Institute and withdrawn on 26-Apr-2022. Version 3.0 will be submitted instead, and changes included in **Version 3.0 versus Version 1.0** are indicated below.

  The following changes were included in Version 3.0 compared with Version 1.0 based on comments from the Paul-Ehrlich Institute:

  - Table 1: ECG assessment and safety laboratory added on Day 29.
  - Section 1 and Section 9.3: history of myocarditis after mRNA vaccinations (Exclusion Criterion 17) and history of mRNA vaccination associated AEs that were in nature and severity beyond the common AEs expected (Exclusion Criterion 18) added as exclusion criteria; exclusion of participants with a history of myocarditis after mRNA vaccinations are also added as risk mitigation measurement in Section 8.5.
  - Section 5.2.1 and Section 8.5: added that ORFV-based vectors have not yet been used in humans.
  - Section 8.5 updated: risk of ineffective vaccination and cardiac toxicity of Prime-2-CoV_Beta added.
  - Section 8.4: clarified that the myocardial necrotic and inflammatory changes observed in rabbits after 3 vaccinations with Prime-2-CoV are considered to be an acute, short-lived and transient event.
  - Section 11.1.5: more details added on how SRC decides on dose escalation
  - Section 14.5, Table 4:
    - Coagulation parameters adapted, fibrinogen, high-sensitive C-reactive protein und biochemistry added.
  - To minimize the risk for participants of not receiving the most effective vaccination according to current recommendations, participant population to be included was changed from fully vaccinated to fully vaccinated + having received booster vaccination with Comirnaty with the booster dose being administered at least 10 weeks before Day 1; Sections 1, 8.1, 8.2, 8.5, and 9.2 (inclusion criteria 2) changed accordingly.
  - Vaccination schedule modified with a 48-hour observation period for the sentinel participant and further 48-hour observation periods between Participants 2+3 and

Participants 4-7, and between Participants 4-7 and Participants 8-12. Vaccinations will be performed with at least a 30-minute interval between participants in the last 2 participant groups. Additional phone visit on Day 3 included, due to the 48-hour observation period. The observation period for which safety data must be available to decide on dose escalation extended from at least 3 days to at least 7 days post vaccination. Sections 1, 8.1, 8.2, 8.4, 11.1.5, 12.5, 14.8, Table 1 and Figure 1 changed accordingly.

- Section 1 and Section 7: Assessment of neutralizing antibodies versus the SARS-CoV-2 variants Beta, Delta and Omicron added as new objectives and endpoints under further exploratory research analyses; clarified that under secondary endpoints neutralizing antibodies versus the Wuhan wild type are assessed; Sections 13 and 15.9 adjusted accordingly as applicable; in Section 13 also added that all tests are qualified and, when available, validated.
- Section 11.2: clarified that Comirnaty is allowed ≥10 weeks before Day 1 (instead of 'between 6 and 8 months).
- Section 14.7 Cardiac safety monitoring added to ensure sufficient cardiac monitoring during the study. Section on ECGs moved from Section 14.6 to Section 14.7; heading of Section 14.6 adjusted to reflect that change. Added that in case of troponin and/or CK-MB laboratory abnormalities and/or whenever clinical symptoms suggestive of cardiac injury occur an ECG as well as further sensitive diagnostic procedures (e.g., cardiac MRI) must be performed; Table 1, Sections 8.2, 8.4, 8.5, 11.1.5, and 14.5 changed accordingly as far as applicable considering new cardiac monitoring.

- Version 4.0, dated 17-May-2022

  The following changes were included in **Version 4.0 compared with Version 3.0** (all these changes were already included in Version 2.0)

  - Transthoracic echocardiography added as additionally cardiac safety monitoring tool at Screening and Month 3. Sections 8.2, 8.4, 8.5, 12.4, 14.7, 15.11, Table 1 changed accordingly.
  - Section 1: justification for starting dose and escalation step added; Section 8.3 changed accordingly.
  - Section 1 and Section 9.2: (Inclusion Criterion 4): body mass index limited to 30.0 kg/m² instead of 32.0 kg/m².
  - Section 1 and Section 22: study periods updated.
  - Section 2: Statistician changed.
  - Section 5.1: updated.
  - Sections 8.2: rationale for the use of Comirnaty extended.

- Section 8.5: outlined why the ORFV-based vector should have a favorable risk profile.
- Section 11.1.1: concentration of stock IP solutions added; for dilution of stock dilution referred to IP manual.
- Section 12.9: added that if needed ECGs and blood collections can also be performed during home visits.
- Section 14.2: in the sentence 'If pregnancy occurs, further vaccination will be discontinued but participants should stay in the study', 'further vaccination will be discontinued but' deleted as only 1 vaccination planned in the study.
- Section 14.5, Table 4: clarified that all parameters will be performed for both male and female participants.
- Previous Section 14.7 (vaccination holding rules) now Section 11.1.6 and new holding rule added i.e.:

  'Any participant experiences a clinically significant increase in CK-MB and/or troponin, a respective abnormal finding in the ECG, and/or relevant clinical symptoms suggestive of cardiac injury within 28 days after IP administration'.
- Minor corrections on wording and formatting were done throughout the document.

The following changes were included in **Version 4.0 compared with Version 3.0 and Version 2.0**:

- Section 11.2: clarified that the date of the 3 (instead of 2) vaccinations with Comirnaty will be documented.
- Section 12.3: clarified that participants are considered enrolled at the time of signing the consent form as indicated in Sections 1 and 9.2, and not at the time of vaccination, as erroneously stated in Section 12.3.
- Section 14.4: clarified that study personnel will review the diary with the participants additionally at 2 days (telephone) after Prime-2-CoV_Beta booster vaccination.
- Section 14.1.1: 'first' was deleted in the following sentences:
  - '….the time when that participant is ~~first~~ administered IP are defined as "**pre-treatment-emergent**" events'
  - 'All unsolicited AEs which occur after the ~~first~~ administration of IP are defined as **TEAEs'.**

- Version 5.0, dated 15-Jul-2022

  To account for the rapidly changing Corona situation and to accelerate participant recruitment that ensures a timely completion of the study, respective inclusion criteria were adapted and slightly modified (as compared to Version 4.0).

  - The permitted number of prior COVID-19 vaccinations is changed to at least 3 vaccinations (instead of 3 vaccinations) to allow subjects with 4 or more vaccinations to participate in the study.
  - Previously allowed COVID-19 vaccination is no longer restricted to the Pfizer/BioNTech-BNT162b2 vaccine (Comirnaty) and is now expanded to include all licensed RNA-based vaccines, which on the one hand still ensures a relatively homogeneous study cohort but on the other hand facilitates recruitment.
  - Subjects with a prior SARS-CoV-2 infection may now also be included, as the number of subjects infected with SARS-CoV-2 has meanwhile tremendously increased. Subjects with acute SARS-CoV-2 infections are still excluded.

  It is not anticipated that any of the outlined changes will affect participant safety or negatively impact the validity of the study results. The following sections were changed accordingly:

  - Section 1: methodology updated accordingly, Inclusion Criterion 2 changed (specifying that full vaccination means at least 3 doses of a licensed mRNA COVID-19 vaccine); Exclusion Criterion 2 modified (adapted to allow all mRNA COVID-19 vaccines as previous vaccinations); Exclusion Criterion 7 (i.e. Confirmed [by real-time quantitative polymerase chain reaction] SARS-CoV-2 infection after 2nd vaccination with Comirnaty) deleted
  - Section 4 updated
  - Sections 8.1, 8.2, 8.5, and 11.2 updated to include that all licensed mRNA-based COVID-19 vaccines are allowed for initial vaccination and that full course of vaccination means at least 3 vaccinations with a licensed mRNA-based COVID-19 vaccine
  - Section 9.2: Inclusion Criterion 2 changed specifying that full course of vaccination means at least 3 doses of a licensed mRNA COVID-19 vaccine
  - Section 9.3: Exclusion Criterion 2 modified (adapted to allow all mRNA COVID-19 vaccines as previous vaccinations); Exclusion Criterion 7 (Confirmed [by real-time quantitative polymerase chain reaction] SARS-CoV-2 infection after 2nd vaccination with Comirnaty) deleted

1 Summary and flow chart

Study title

A phase Ia, dose-finding study to assess the safety and immunogenicity of an Orf virus-based COVID-19 vaccine booster (Prime-2-CoV_Beta) in healthy adults

Study code

PRiME2_21_1

Study centers and countries

The study is planned to be conducted at 2 centers in Germany

Coordinating and chief investigator: [redacted]

Clinical phase

Phase Ia

Study duration (planned)

Planned study start (first participant in): May-2022

Planned study end (last participant out): Jan-2023

Estimated recruitment period: 4 months

Study periods

Screening: Day -22 to Day 1 before Prime-2-CoV_Beta booster vaccination; Prime-2-CoV_Beta booster vaccination on Day 1, safety follow-up until Month 6.

Participants are considered enrolled at the time of signing the consent form.

Study objectives and endpoints

Objectives	Endpoints
Primary	
• To assess the safety and tolerability	• Proportion of participants with treatment-emergent adverse events (TEAEs) and serious adverse events (SAEs) throughout the study<br>• Proportion of participants with<br>o Solicited local adverse events (AEs, first 7 days after Prime-2-CoV_Beta booster vaccination): pain at injection site, redness, induration, and swelling<br>o Solicited systemic AEs (first 7 days after Prime-2-CoV_Beta booster vaccination): fever, fatigue, headache, chills, vomiting, nausea, diarrhea, new or worsened muscle pain, new or worsened joint pain

Objectives	Endpoints
Primary (continued)	
	○ Unsolicited TEAEs throughout the study
Secondary	
• To assess the immune response elicited by Prime-2-CoV_Beta as booster vaccination<br>○ Based on neutralizing antibodies	• Level of neutralizing antibody titers versus SARS-CoV-2 (Wuhan wild type) at each post-booster vaccination assessment[a]<br>• Geometric mean fold rise (GMFR) of neutralizing antibodies (versus Wuhan wild type) from Baseline to each post-booster vaccination assessment[a]
○ Based on receptor-binding protein [RBD] antibodies	The following will be assessed at each post-booster vaccination assessment[a]:<br>• Immunoglobulin (Ig)G antibody titer versus SARS-CoV-2 RBD<br>• Geometric mean titers (GMT) of RBD-specific IgG antibodies<br>• GMFR of RBD-specific IgG antibodies from Baseline
• To assess further safety and tolerability parameters	• Proportion of participants with AEs of special interest throughout the study
**Further exploratory research analyses**	
• To assess the immune response elicited by Prime-2-CoV_Beta as booster vaccination<br>○ Based on S-specific antibodies	The following will be assessed at each post-booster vaccination assessment[a]:<br>• IgG antibody titer versus SARS-CoV-2 S1 protein<br>• GMT of IgG S1-specific antibodies<br>• GMFR of IgG S1-specific antibodies from Baseline

Objectives	Endpoints
**Further exploratory research analyses *(continued)***	
○ Based on nucleocapsid (N)-specific antibodies	The following will be assessed at each post-booster vaccination assessment[a]<br>• IgG antibody titer versus SARS-CoV-2 N-protein<br>• GMT of N-specific IgG antibodies<br>• GMFR of N-specific IgG antibodies from Baseline
○ Based on neutralizing antibodies	• Level of neutralizing antibody titers versus the Variants of Concern SARS-CoV-2_Beta, SARS-CoV-2_Delta and SARS-CoV-2_Omicron at each post-booster vaccination assessment[a]<br>• GMFR of neutralizing antibodies from Baseline to each post-booster vaccination assessment[a]
○ Based on protein specific T-cell response	• Percentage of cytokine producing S and N protein-specific T-cells compared to Baseline
○ Based on further parameters	• Additional assays to measure the immune response to Prime-2-CoV_Beta and immune response to the ORFV vector backbone

For all endpoints, Baseline will be defined as last measurement before Prime-2-CoV_Beta booster vaccination.
[a] With respective immunogenicity assessments.

Methodology

Overall study design

This is an open-label, first-in-human, dose-finding study to evaluate the safety and immunogenicity of a booster vaccination of Prime-2-CoV_Beta in healthy participants who had received the full course of vaccination, including booster vaccination (i.e., having received at least 3 doses) with a licensed messenger ribonucleic acid (mRNA)-based COVID-19 vaccine. Eligible participants will undergo baseline assessments and will receive 1 injection of Prime-2-CoV_Beta at Day 1. Participants will be followed up through 6 months post-booster vaccination. Follow-up visits will be performed at Days 4, 8, 15, 29, and Months 3 and 6, to assess the safety, tolerability, and immunogenicity of Prime-2-CoV_Beta. Additional safety and tolerability data will be assessed 1 day and 2 days after booster vaccination (Days 2 and 3) by telephone. A total of 60 participants is planned to be vaccinated in 5 cohorts of 12 participants each. Dose ranging of Prime-2-CoV_Beta will be done by dose escalation with doses ranging from $3x10^4$ plaque forming units (PFUs) up to $3x10^7$ PFUs (see Dose cohorts below).

All participants will record solicited AEs (local and systemic) in an electronic diary starting on the day of Prime-2-CoV_Beta booster vaccination throughout an additional 7 days (not counting vaccination day).

A safety review committee (SRC) will be established that will review safety data after the last participant of each of the Cohorts 1 to 5 has completed Day 8 and ad hoc in case holding rules apply. Further ad hoc SRC meetings may be scheduled upon request (e.g., for potential safety issues).

Vaccination sequence and dosing schedule

Cohorts 1 to 5 will include a safety lead with 1 sentinel participant. This sentinel participant will stay at the center for the first 4 hours and will be followed up for at least an additional 44 hours after Prime-2-CoV_Beta booster vaccination. One day after vaccination and at the end of the 48-hour observation period, center personnel will contact the participant by telephone to obtain information on solicited and unsolicited AEs and other safety issues. If no safety issues occurred within the on-site monitoring period as assessed by the investigator and solicited during the telephone visits at Days 2 and 3, the next 2 participants (Participant 2 and 3) in that dose cohort will be vaccinated with an interval of at least 4 hours between vaccinations. After an additional 48-hour observation period and assuming no safety issues were identified in these 2 participants (during the on-site monitoring or phone visits), an additional 4 participants will be vaccinated with at least 30 minutes between vaccinations. After a further 48-hour observation period, and assuming that no safety problems were noted in these 4 participants (during the on-site monitoring or phone visits), the remaining participants in the dosing group will be vaccinated with an interval of at least 30 minutes between vaccinations. Each participant will be observed for at least 4 hours at the study center after Prime-2-CoV_Beta booster vaccination.

After the last participant of each of the Cohorts 1 to 4 has completed 7 days of follow up after the Prime-2-CoV_Beta booster vaccination, all safety data available including full cardiac assessments and complete safety laboratory will be reviewed by the SRC. The independent members of the SRC will then provide recommendations whether to proceed with dose escalation or not. The SRC will also review Cohort 5 safety data after all Cohort 5 participants have completed 7 days of follow up and provide recommendations whether any modifications to study conduct should be implemented.

If a holding rule applies, further vaccination in any group will be paused until an ad hoc safety review by the SRC has been performed and conclusions and suggestions for potential measures were communicated to the sponsor. If considered appropriate, vaccination will be resumed. The safety review will consider e.g., the relationship of the AE or SAE to Prime-2-CoV_Beta, new and relevant safety information from ongoing research programs with Prime-2-CoV_Beta, and additional screening assessments to identify those participants who may develop similar symptoms.

Investigational products, dose cohort, and number of participants

Prime-2-CoV_Beta, dose ranging from $3x10^4$ to $3x10^7$ PFU, 1 intramuscular injection (1.0 mL each) into the deltoid muscle on Day 1.

As the vaccine has not been used in humans before and participants are healthy volunteers, the minimal anticipated biological effect level (MABEL) approach has been used to select the starting dose to ensure safety of the participants (EMA Guideline on strategies to identify and mitigate risks for first-in-human and early clinical trials with investigational medicinal products. EMEA/CHMP/SWP/28367/07 Rev. 1).

In mice, initial pharmacological responses (i.e., an immune response) were observed with 2 doses of about $5x10^4$ PFU/animal given 3 weeks apart. A starting dose in the low range of pharmacological activity is anticipated. An escalation by factor 10 per dose is justified by the excellent tolerability profile and very low physiological reactions in the various species and doses tested. The highest intended human dose of $3x10^7$ PFU, administered as total dose/animal, was tolerated well without local or adverse reactions in all species tested following a single administration.

Dose cohorts

Single booster dose as follows:

Cohort	Dose (PFU)	Number of participants
1	$3x10^4$	12
2	$3x10^5$	12
3	$3x10^6$	12
4	$1.5x10^7$	12
5	$3x10^7$	12
**All**		**60**

Depending on safety and immunogenicity data obtained in an informal analysis after all participants have completed Day 15 (see Statistical methods) further cohorts may be opened (after approval of a respective protocol amendment).

Participant population

Healthy adult men and women

Inclusion criteria

1. Healthy adult men or women aged 18 to 55 years
2. Full course of vaccination, including booster vaccination (i.e., having received at least 3 doses) with a licensed mRNA COVID-19 vaccine, with the last dose being administered at least 10 weeks before Day 1 as documented in a respective vaccination certificate
3. Able to understand the participant information and providing written informed consent

4. Body mass index of 18.5 to 30.0 kg/m² and weight > 50 kg at Screening
5. Women of childbearing potential must:
   a. have a negative pregnancy test at Screening (blood) and at Day 1 (urine)
   b. agree to use, and be able to comply with, highly effective measures of contraception without interruption, from 14 days before Prime-2-CoV_Beta booster vaccination until the end of the study.

      A highly effective method of contraception or birth control (failure rate less than 1% per year when used consistently and correctly) for this study: combined (estrogen and progestogen containing) hormonal contraception associated with inhibition of ovulation (oral, intravaginal, transdermal, injectable), progestogen-only hormonal contraception associated with inhibition of ovulation (oral, injectable, implantable), intrauterine device, intrauterine hormone-releasing system, bilateral tubal occlusion, sexual abstinence or vasectomized sexual partner. Abstinence is only acceptable as true abstinence when this is in line with the preferred and usual lifestyle of the participant (abstinent on a long-term and persistent basis). The reliability of sexual abstinence needs to be evaluated in relation to the duration of the clinical study and the preferred and usual lifestyle of the participant. (Periodic abstinence [e.g., calendar, ovulation, symptothermal, post-ovulation methods and withdrawal] are not acceptable methods of contraception.)

      Postmenopausal (no menses for at least 1 year without alternative medical cause) or surgically sterile women (tubal ligation, hysterectomy or bilateral oophorectomy) may be enrolled.
6. Male participants must agree not to intend to father a child or to donate sperm starting at Screening, throughout the clinical study. Male participants must also
   a. abstain from sexual intercourse with a female partner (acceptable only if it is the participant's usual form of birth control/lifestyle choice: abstinent on a long-term and persistent basis). The reliability of sexual abstinence needs to be evaluated in relation to the duration of the clinical study and the preferred and usual lifestyle of the participant or
   b. use adequate barrier contraception (male condom) during treatment with the investigational product until the end of the study, and
   c. ensure that, if they have a female partner of childbearing potential, the partner uses a highly effective contraceptive method as outlined in inclusion criterion number 5
   d. use condoms during the entire study if they have a pregnant partner, to avoid exposure of the fetus to the investigational product

7. Willing and able to comply with all study procedures based on the investigator's judgment

Exclusion criteria

Previous and concomitant therapy

1. Receipt of any vaccine (licensed or investigational) from 4 weeks before Prime-2-CoV_Beta booster vaccination or anticipated vaccination during the study until 6 weeks after the Prime-2-CoV_Beta booster vaccination
2. Previous vaccination against COVID-19 with vaccines (licensed or investigational) other than mRNA-based vaccines (licensed or investigational)
3. Current or previous treatment with another investigational drug and/or medical device (within 30 days of enrollment or 5 half-lives of that investigational drug)
4. Administration of immunoglobulins or any blood products within 2 months of Prime-2-CoV_Beta booster vaccination
5. Chronic administration of medication associated with impaired immune responsiveness as judged by the investigator (including, but not limited to: immunosuppressive therapy, systemic corticosteroids exceeding 10 mg/day of prednisone equivalent, allergy shots for hypo-sensitization, immunoglobulin, interferon, immunomodulators, cytotoxic drugs, or other similar or toxic drugs) within 2 months before the Prime-2-CoV_Beta booster vaccination (Day 1). Inhaled/nebulized, intra-articular, intrabursal, or topical (skin or eyes) corticosteroids are permitted.

Previous and concomitant medical condition

6. Active SARS-CoV-2 infection, confirmed by a commercially available SARS-CoV-2 rapid antigen test at Day 1, or currently on quarantine
7. *Deleted in protocol Version 5.0*
8. Known history of severe adverse reactions to any vaccine and/or severe allergic reactions to any component of the study vaccine, to any drug, or to any other exposure
9. Known history of angioedema
10. Pregnant or lactating women
11. Any confirmed or suspected immunosuppressive or immunodeficient condition
12. Known history of Guillain-Barré Syndrome
13. Known infection with human immunodeficiency virus, hepatitis C virus or hepatitis B virus
14. Active cancer (malignancy) within 5 years before Day 1 (except for adequately treated non-melanomatous skin carcinoma, at the discretion of the investigator)

15. Moderate or severe illness and/or fever > 38.0 °C within 1 week before Prime-2-CoV_Beta booster vaccination
16. Any clinically significant health problem (medical history and physical examination) or clinically significantly abnormal finding in biochemistry and/or hematology blood tests, urinalysis, or electrocardiogram at Screening according to the investigator's opinion
17. Current or history of cardiovascular disease or structural cardiac disease (including chronic or congenital heart conditions, such as chronic hypertension, coronary heart disease, myocardial infarction and arrhythmias, hypertrophic cardiomyopathy, as well as a history of myocarditis after mRNA vaccinations)
18. History of mRNA vaccination-associated AEs that were in nature and severity beyond the common AEs expected
19. Current or history of gastrointestinal disease, liver disease, renal disease or endocrine disorders, (including diabetes) and neurological illness (excluding migraine), when judged as clinically significant according to the investigator's opinion
20. Current or history of chronic respiratory diseases, including mild asthma treated by on-demand medication (resolved childhood asthma is allowed)
21. Current or history of alcohol and/or drug abuse within the last 6 months before Day 1

Previous and concomitant clinical study experience

22. Current participation in another study or previous enrollment in this clinical study

Other exclusion criteria

23. Investigator or employee of the study group or sponsor with direct involvement in the proposed study or relatives of the research staff with direct involvement in the proposed study
24. Prolonged exposure to sheep or goats (e.g., shepherds, sheep farmer)

Statistical methods

The sample size is based on clinical and practical considerations and not on statistical hypothesis testing. The sample size is considered sufficient to evaluate the objectives of the study.

All analyses will be done using descriptive methods.

No formal interim statistical analysis is planned. Safety data will be analyzed periodically for SRC review. An informal analysis of all collected data will be performed when all participants have been followed up for at least 14 days after Prime-2-CoV_Beta booster vaccination to determine the right dose group to move into an extension period and/or phase II. In case of any safety concerns, an additional safety analysis will be performed.

Figure 1: Dosing and vaccination schedule

Dosing schedule

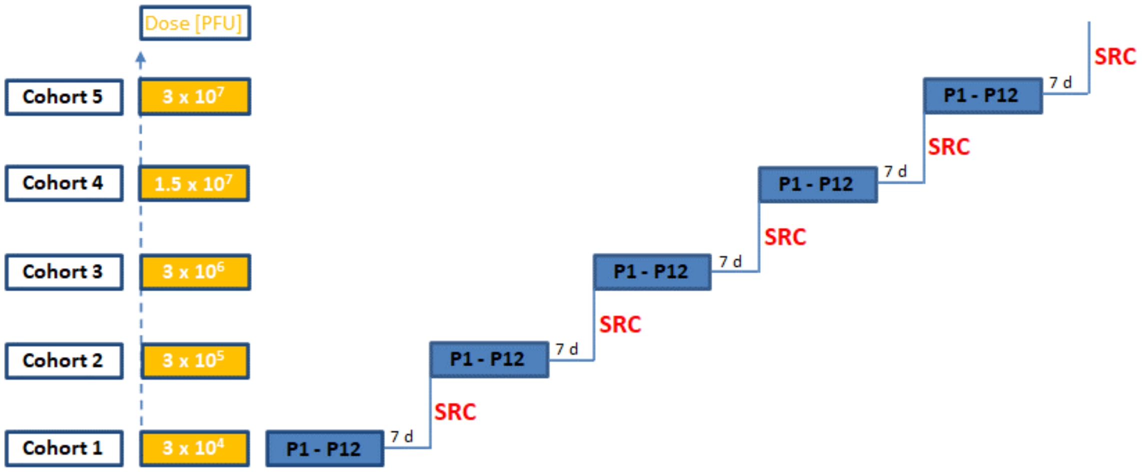

Vaccination schedule

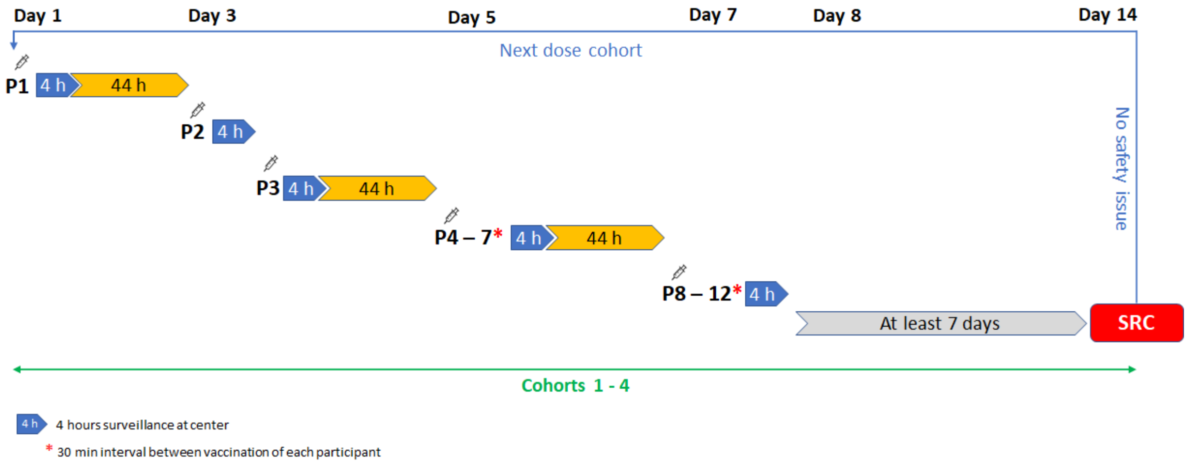

Time periods are minimum time spans.
d = day, SRC = safety review committee, P = participant.

Table 1: Schedule of assessments

Procedures	V0[a]	V1[a]	TV1	TV2	V2	V3	V4	V5	V6	V7	Unsch.
	Screen. D-22 to D1	D1	D2* 1d post-booster	D3* 2ds post-booster	D4	D8 ±1 d	D15 (W2) ±2 d	D29 (W4) ±1 d	M3 ±14 d	EoS M6±14 d	Visit
Informed consent	x										
In- and exclusion criteria	x	x[c]									
Demography	x										
Medical history[b]	x										
Height and weight (BMI)	x										
Physical examination	x	x[c]						x[d]		x[d]	x[d]
Vital signs	x	x[c]				x	x	x			x
12-lead ECG[j]	x					x		x		x	
Transthoracic echocardiography	x								x		
SARS-CoV-2 rapid Ag-test		x[c]									
Vaccination		x									
Pregnancy test[e]	x	x[c.f]									
Assess vital signs at center discharge[g]		x									
e-Diary instructions, provide measuring tool and thermometer		x									
Diary review			x	x	x	x					
Safety laboratory[h]	x				x	x		x		x	
Serology[i]	x										
Blood sampling for											
Humoral immunogenicity		x[c]				x	x	x	x	x	
Cellular immunogenicity		x[c]				x	x	x		x	
Plasma cytokine profile		x[c]			x						
Anti-vector immunity		x[c]					x	x		x	

Schedule of assessments *(continued)*

Procedures	V0[a]	V1[a]	TV1	TV2	V2	V3	V4	V5	V6	V7	Unsch.
	Screen. D-22 to D1	D1	D2* 1d post-booster	D3* 2ds post-booster	D4	D8 ±1 d	D15 (W2) ±2 d	D29 (W4) ±1 d	M3 ±14 d	EoS M6±14 d	Visit
Previous and concomitant medications	x	x--x								x	x
AEs, SAEs		x--x								x	x
Additional diagnostic work-up to monitor cardiac safety (e.g., ECG, more sensitive methods like cardiac MRI)		In case of abnormalities in cardiac biomarkers (creatine kinase-MB, troponin) and/or whenever clinical symptoms suggestive of cardiac injury occur									

* Telephone call 24 hours and 48 hours after Prime-2-CoV_Beta booster vaccination to assess safety.

[a] May be performed on the same day. If performed on the same day, assessments scheduled for both visits must be performed only once.

[b] Including previous SARS-CoV-2 infection and vaccination, and any connected symptoms.

[c] Performed before vaccination.

[d] Only symptom directed.

[e] Only for women of childbearing potential. Pregnancy testing will include a blood test at Screening and a urine test at Day 1.

[f] Negative test result must be available before vaccination. Result must not be older than 24 hours.

[g] All participants will be observed at the study center for at least 4 hours post Prime-2-CoV_Beta booster vaccination. Before discharge, vital signs will be assessed.

[h] Including hematology and biochemistry (including cardiac biomarkers and coagulation), and at Screening additionally urinalysis (dip stick). In case of abnormalities in cardiac biomarkers (creatine kinase-MB, troponin) and/or whenever clinical symptoms suggestive of cardiac injury occur further diagnostic work-up including ECG and/or more accurate and sensitive methods for cardiovascular disease diagnosis must be performed.

[i] Screening for HIV, HBV, HCV infection.

[j] An unscheduled ECG should be performed whenever clinical symptoms of cardiac problems occur, or in case of cardiac biomarker abnormalities.

AE = adverse event, Ag = antigen, BMI = body mass index, D = day, d = day(s), ECG = electrocardiogram, EOS = end of study, HBV = hepatitis B virus, HCV = hepatitis C virus, HIV = human immunodeficiency virus, M = month, min = minutes, MRI = magnetic resonance imaging, SAE = serious adverse event, SARS-CoV-2 = severe acute respiratory syndrome coronavirus-2, Screen. = screening, TV = telephone visit, unsch. = unscheduled, V = visit, W = week.

.

2 Addresses and responsibilities

**Sponsor's Responsible Medical Officer**	[redacted]<br>University Hospital Tübingen<br>Institute of Tropical Medicine<br>Wilhelmstr. 27<br>72074 Tübingen<br>Germany<br>[redacted]
**Chief investigator (coordinating investigator, Germany)**	[redacted]<br>University Hospital Tübingen<br>Institute of Tropical Medicine<br>Wilhelmstr. 27<br>72074 Tübingen<br>Germany<br>[redacted]
**Statistician**	[redacted]<br>FGK Clinical Research GmbH<br>Heimeranstr. 35<br>80339 München<br>Germany<br>[redacted]
**Monitoring, Drug Safety, and Data Management**	FGK Clinical Research GmbH<br>Heimeranstr. 35<br>80339 München<br>Germany<br>[redacted]

A complete list of study personnel will be available in the trial master file.

3 Table of contents

Page

REVISION CHRONOLOGY 2

1 SUMMARY AND FLOW CHART 6

2 ADDRESSES AND RESPONSIBILITIES 17

3 TABLE OF CONTENTS 18

4 ABBREVIATIONS AND DEFINITION OF TERMS 22

5 INTRODUCTION 24

5.1 The disease - COVID-19 24

5.2 The ORFV-based COVID-19 vaccine candidate - Prime-2-CoV_Beta 25

5.2.1 Composition 25

5.2.2 Nonclinical studies 26

6 INVESTIGATORS, STUDY ADMINISTRATIVE STRUCTURE, AND STUDY COMMITTEES 27

7 STUDY OBJECTIVES AND ENDPOINTS 27

8 STUDY DESIGN AND DESIGN RATIONALE 30

8.1 Overall study design 30

8.2 Study design rationale 30

8.3 Justification of dose 32

8.4 Justification for post vaccination surveillance period 33

8.5 Risk-benefit assessment 37

9 STUDY POPULATION 40

9.1 Sample size 40

9.2 Inclusion criteria 40

9.3 Exclusion criteria 41

9.4 Criteria for delaying vaccination 43

9.5 Re-screening 43

10 RANDOMIZATION, BLINDING AND UNBLINDING PROCEDURES 44

10.1 Randomization and blinding 44

10.2 Participant identification 44

11 TREATMENTS 44

11.1 Investigational products 44

11.1.1 Test product 45

11.1.2 Description, packaging and labeling 46

11.1.3 Storage and stability 46

11.1.4 Treatment dose, dose cohorts, and administration 46

11.1.5 Sequence of vaccination and dose escalation 47

11.1.6 Vaccination holding rules 48

11.1.7 Investigational product accountability and compliance 49

11.2 Previous and concomitant medication 49

12 STUDY SCHEDULE 50

12.1 Study conduct 50

12.2 Screening (Days -22 to Day 1) 51

12.3 Day 1 (Baseline) 51

12.4 Subsequent clinic visits 51

12.5 Telephone visits 51

12.6 Unscheduled visits 52

12.7 End-of-study visit and early termination 52

12.8 Medical care upon termination of the clinical study 52

12.9 Restrictions due to pandemics 52

13 IMMUNOGENICITY ASSESSMENTS 53

14 SAFETY ASSESSMENTS AND PROCEDURES 54

14.1 Adverse events 54

14.1.1 Definitions 54

14.1.2 Adverse events of special interest (AESI) 55

14.1.3 Classification of adverse events 56

14.1.4 Documentation of adverse events 57

14.1.5 Reporting of serious adverse events and suspected unexpected serious adverse reactions 58

14.1.6 Follow-up of adverse events 59

14.2 Pregnancy 60

14.3 Medication errors ... 60
14.4 Diary ... 61
14.5 Clinical safety laboratory investigations ... 62
14.6 Vital signs and physical examination ... 63
14.7 Cardiac safety monitoring ... 64
14.8 Safety monitoring ... 65

15 BIOSTATISTICAL METHODS ... 65
15.1 Sample size calculation ... 65
15.2 General approach ... 65
15.3 Handling of missing data ... 65
15.4 Analysis sets and types of analyses ... 66
15.5 Analysis of study conduct and participant disposition ... 66
15.6 Analysis of baseline characteristics ... 66
15.7 Analysis of the primary endpoints ... 66
15.8 Analyses of secondary immunogenicity endpoints ... 67
15.9 Further exploratory research analyses ... 67
15.10 Analyses of anti-vector immunity ... 67
15.11 Safety analyses ... 68
15.12 Interim analysis ... 69

16 PARTICIPANT WITHDRAWAL ... 70
16.1 Replacement of participants ... 70

17 ETHICAL AND LEGAL REQUIREMENTS ... 71
17.1 General requirements ... 71
17.2 Independent ethics committees ... 71
17.3 Participant information and consent procedure ... 71
17.4 Insurance coverage ... 72
17.5 Submission to authorities ... 72
17.6 Participant confidentiality ... 72

18 CRITERIA FOR PREMATURE TERMINATION OF THE STUDY AND CRITERIA FOR INITIALIZING AND CLOSING A STUDY CENTER ... 73
18.1 Criteria for terminating the clinical study ... 73

18.2 Criteria for closing a study center ... 73

19 STUDY PROTOCOL, DOCUMENTATION AND ARCHIVING OF DATA ... 74

19.1 Amendments to the protocol ... 74

19.2 Protocol deviations ... 74

19.3 Data retention ... 75

20 DATA COLLECTION, MONITORING AND QUALITY ASSURANCE ... 75

20.1 Data collection ... 75

20.2 Monitoring ... 76

20.3 Audits and inspections ... 77

20.4 Data management procedures ... 77

20.5 Training of center staff ... 78

21 STUDY REPORT AND PUBLICATIONS ... 78

22 STUDY PERIODS ... 78

23 REFERENCES ... 79

24 APPROVAL AND SIGNATURES ... 82

25 APPENDICES ... 84

APPENDIX 1 GRADING SCALES ... 84

APPENDIX 2 POTENTIAL IMMUNE-MEDIATED DISEASES ... 88

APPENDIX 3 ADVERSE EVENTS OF SPECIAL INTEREST FOR SARS-COV-2 VACCINES ... 91

4 Abbreviations and definition of terms

ADE	Antibody-dependent enhancement
ADR	Adverse drug reaction
AE	Adverse event
AESI	Adverse event of special interest
AR	Adverse reaction
CA	Competent authority
CBER	Center for Biologics Evaluation and Research
CI	Confidence interval
CK-MB	creatine kinase-MB
COVID-19	Corona virus disease 2019
CS	Clinically significant
ECG	Electrocardiogram
eCRF	Electronic case report form
EoS	End-of-study
GCP	Good Clinical Practice
GMFR	Geometric mean fold rise
GMT	Geometric mean titers
ICF	Informed consent form
ICH	International Council for Harmonization of Technical Requirements for Pharmaceuticals for Human Use
IEC	Independent ethics committees
Ig	Immunoglobulin
IM	Intramuscular
IMS	Immunogenicity set
IP	Investigational product
ISF	Investigator Site File
LLOQ	Lower limit of quantification
MDRD	Modification of Diet in Renal Disease
MedDRA	Medical Dictionary for Regulatory Activities
MRI	Magnetic resonance imaging
mRNA	messenger ribonucleic acid
N	Nucleocapsid
NCS	Not clinically significant
NHP	Non-human primate
ORFV	Orf virus
PFU	Plaque forming unit
PT	Preferred term

RBD	Receptor-binding protein
S	Spike protein
SAE	Serious adverse event
SAF	Safety analysis set
SARS-CoV-2	Severe acute respiratory syndrome coronavirus-2
SRC	Safety review committee
SUSAR	Suspected unexpected serious adverse reaction
TEAEs	Treatment-emergent adverse events
Th	T helper cells
UKT	University Hospital Tübingen
ULOQ	Upper limit of quantification
VoC	Variant of concern
WHO-DD	World Health Organization Drug Dictionary

5 Introduction

5.1 The disease - COVID-19

The new betacoronavirus severe acute respiratory syndrome coronavirus-2 (SARS-CoV-2) causes the corona virus disease-2019 (COVID-19), a severe acute respiratory and lung syndrome. Since the first reports in China end of 2019, infections with SARS-CoV-2 have rapidly spread across other countries resulting in a worldwide COVID-19 pandemic, challenging the global health care systems and economies. As of Week 10, 2022 over 450 million cases have been identified globally with more than 6.0 million fatalities. [1] Transmission of SARS-CoV-2 between people occurs primarily via respiratory (droplet and aerosol) routes.

COVID-19 presents with a great variety of symptoms, the most common ones being fever, continuous cough, shortness of breath, fatigue, loss of appetite, diarrhea, nausea and vomiting, anosmia (loss of smell) and ageusia (loss of taste). Critical complications with COVID-19 include acute respiratory distress syndrome, sepsis, septic shock, cardiac disease, thromboembolic events, such as pulmonary embolism and multi-organ failure, neurologic complications (e.g., encephalopathy), and inflammatory complications (exuberant inflammatory response). [2,3] Current data indicate that among people who develop symptoms 40% have mild symptoms without hypoxia or pneumonia, 40% have moderate symptoms and non-severe pneumonia, 15% have significant disease including severe pneumonia, and 5% experience critical disease with life-threatening complications. [2]

Severe illness may occur in otherwise healthy individuals of any age, but is primarily observed in the elderly and adults with certain underlying medical comorbidities (e.g., cardiovascular disease, diabetes mellitus, hypertension, chronic lung disease, cancer, chronic kidney disease, obesity, and smoking). [3]

Patients who have suffered from both mild or severe COVID-19 can experience prolonged symptoms or develop long-term complications (long COVID) including (but not limited to) respiratory and cardiovascular symptoms and conditions, protracted loss or change of smell and taste, mental health problems including depression, anxiety and cognitive difficulties, fatigue, weakness and sleeplessness.[2]

Although effective vaccines are available new vaccines eliciting broad, strong, and long-lasting immune responses, and which are less prone to mutations of the virus are still needed.

5.2 The ORFV-based COVID-19 vaccine candidate - Prime-2-CoV_Beta

5.2.1 Composition

Viral vector-based vaccines have the potential to elicit strong cellular and humoral immune responses. Within the genus Parapoxvirus of the Poxviridae, the Orf virus (ORFV) strain D1701-VrV comprises various properties particularly favorable for the development of a vector platform technology and facilitates various vaccination approaches:

- The intrinsic, ORFV vector-mediated effect of activating and stimulating innate immune mechanisms resulting in the induction of strong adaptive immune response obviates the need for adjuvants.
- Induction of robust cellular and humoral immune responses, in the absence of adjuvants, that mediate full protection against challenge infection as shown in several animal experiments in > 10 different species. [4-8]
- Development of an immunological memory that provides long-lasting protection against infections. [4]
- Because ORFV is known not to generate lasting anti-vector immunity, this viral platform, in contrast to other viral vector, is thought to allow repeated vaccinations. This will allow for adapting the vaccine to emerging strains/variants, such as SARS-CoV-2 Variant of Concern (VoC). In addition, there is (almost) no prevalence of immunity to wildtype strain ORFV in the population.
- Fully attenuated; completely avirulent even in immunocompromised primary host (sheep); no integration into the host genome possible, since poxvirus per se replicate in the cytoplasm and do not enter the nucleus; biological safety level category 1.
- Promising thermal stability at 4 °C and a formulation supporting lyophilization offering technical and logistical advantages.
- Large capacity to stably insert transgenes into ORFV genome: Due to the size of poxviral vectors including ORFV (approximately 150 kbp), the generation of polyvalent vaccines with up to 5 different, stably integrated transgenes of up to 10 kbp in total is feasible.

No ORFV-based vaccine has yet been approved for human use or administered to humans and thus there is no previous experience with the vector in humans. However, the vector used itself is not capable of replication *in vivo* (see above) and is assigned to biological safety level 1.

SARS-CoV-2 consists of a spherical, enveloped, large positive-sense single-stranded ribonucleic acid genome encoding 16 non-structural proteins and 4 structural proteins, i.e., the spike (S) glycoprotein, envelope, membrane and nucleocapsid (N) proteins.

Using the ORFV vector platform, an anti-COVID-19 recombinant vaccine candidate (Prime-2-CoV_Beta) was produced that encodes the following 2 genes of SARS-CoV-2:

- **The structural S protein** which is responsible for entry of the virus and a main target for virus neutralizing protective immune responses. Moreover, it induces antiviral systemic immunoglobulin G (IgG), local IgA, and lung-resident T-cell responses. [9-12] The receptor-binding domain (RBD) is localized in the center of the S1 subunit. RBD is found to induce robust SARS-CoV-2 neutralizing response. [13] The S2 subunit of the S protein represents an immunogenic protein [9] and seems to be required to elicit full protection. [12] The following mutations have been introduced into the S protein: D614G (change from an aspartate to a glycine residue at position 614, the most dominant form of SARS-CoV-2 worldwide), K986P and V987P (change from lysine or valine respectively to proline, proline stabilization), and AA682-685: RRAR to GSAS (furin cleavage site deletion with R = arginine, A = alanine, G = glycine, S = serine). These mutations have been introduced to stabilize the S protein. [14] In addition, the following mutations to the RBD site have been introduced as they represent the most critical mutations of the SARS-CoV-2_Beta VoC: K417N, E484K, N501Y.
- **The structural N protein** represents another potential target antigen for SARS-CoV-2 and is not expressed at the surface of infected cells. Furthermore, the N protein elicits a long-lasting T-cell response. [9,15,16] One working hypothesis of a multi-antigenic COVID-19 vaccine concept is around the quality of the humoral immune response. Indeed, because of induced CD4 T-cell help, which could be specific to the N or the S protein, affinity maturation and Ig class switch to a more favorable isotype of RBD-specific antibodies known to efficiently neutralize SARS-CoV-2 are expected. High T-cell help shall also contribute to broaden the antibody repertoire for potential enhanced cross-protections against emerging variants. In addition, N-specific CD8 cells could also contribute to strengthen the protection against severe disease.

To broaden the specific immunity and reduce potential immune evasion of SARS-CoV-2, a multivalent vaccine concept was developed that expressed the 2 antigens (S protein, N protein) simultaneously from a single recombinant ORFV vector.

5.2.2 Nonclinical studies

Nonclinical experiments in mice, hamsters and non-human primates demonstrated that the vaccine candidate can elicit a strong humoral and cellular immune response. The responses were comparable or even higher than induced after natural SARS-CoV-2 infection. The induced antibodies were able to effectively neutralize SARS-CoV-2. The response was T helper cell 1-dominated and, thus, limiting the risk of antibody-dependent enhancement (ADE). No severe side-effects upon immunization had been reported for all animals. With doses > 10^7 plaque forming units (PFU) an immune response comparable to that elicited after natural SARS-CoV-2

infection was detectable. With lower doses, a prime-boost regimen was necessary to induce immune responses comparable to that of a natural SARS-CoV-2 infection. More details are provided in the investigator's brochure.

6 Investigators, study administrative structure, and study committees

The clinical study is funded by the German "Bundesministerium für Wirtschaft und Energie (Federal Ministry for Economic Affairs and Energy)" and the "Forschungszentrum Jülich" (Jülich Research Center)". It is planned to be conducted at 2 centers in Germany (1 center each in Tübingen and Hamburg), under the supervision of [redacted], Institute of Tropical Medicine, UKT.

The sponsor, UKT, will be responsible for the overall supervision and administration of the study. Data management, statistical analysis and medical writing services will be done by FGK Clinical Research GmbH, München, Germany (a contract research organization [CRO] and referred to as sponsor' designee throughout this document). Safety laboratory will be done locally. Immunogenicity assays will be done in-house at the sponsor and/or by an outside vendor. The electronic diary and electronic case report form (eCRF) will be provided by an outside vendor.

A safety review committee (SRC) will be established that will monitor safety data. For more details refer to Section 14.8.

7 Study objectives and endpoints

**Objectives**	**Endpoints**
Primary	
• To assess the safety and tolerability	• Proportion of participants with treatment-emergent adverse events (TEAEs) and serious adverse events (SAEs) throughout the study<br>• Proportion of participants with<br>o Solicited local adverse events (AEs, first 7 days after Prime-2-CoV_Beta booster vaccination): pain at injection site, redness, induration, and swelling

Objectives	Endpoints
Primary (continued)	
	○ Solicited systemic AEs (first 7 days after Prime-2-CoV_Beta booster vaccination): fever, fatigue, headache, chills, vomiting, nausea, diarrhea, new or worsened muscle pain, new or worsened joint pain<br>○ Unsolicited TEAEs throughout the study
Secondary	
• To assess the immune response elicited by Prime-2-CoV_Beta as booster vaccination<br>○ Based on neutralizing antibodies	• Level of neutralizing antibody titers versus SARS-CoV-2 (Wuhan wild type) at each post-booster vaccination assessment[a]<br>• Geometric mean fold rise (GMFR) of neutralizing antibodies (versus Wuhan wild type) from Baseline to each post-booster vaccination assessment[a]
○ Based on RBD antibodies	The following will be assessed at each post-booster vaccination assessment[a]:<br>• IgG antibody titer versus SARS-CoV-2 RBD<br>• Geometric mean titers (GMT) of RBD-specific IgG antibodies<br>• GMFR of RBD-specific IgG antibodies from Baseline
• To assess further safety and tolerability parameters	• Proportion of participants with AEs of special interest throughout the study

**Objectives**	**Endpoints**
**Further exploratory research analyses**	
• To assess the immune response elicited by Prime-2-CoV_Beta as booster vaccination<br>○ Based on S-specific antibodies	The following will be assessed at each post-booster vaccination assessment[a]:<br>• IgG antibody titer versus SARS-CoV-2 S1 protein<br>• GMT of IgG S1-specific antibodies<br>• GMFR of IgG S1-specific antibodies from Baseline
○ Based on N-specific antibodies	The following will be assessed at each post-booster vaccination assessment[a]<br>• IgG antibody titer versus SARS-CoV-2 N-protein<br>• GMT of N-specific IgG antibodies<br>• GMFR of N--specific IgG antibodies from Baseline
○ Based on neutralizing antibodies	• Level of neutralizing antibody titers versus the VoC SARS-CoV-2_Beta, SARS-CoV-2_Delta and SARS-CoV-2_Omicron at each post-booster vaccination assessment[a]<br>• GMFR of neutralizing antibodies from Baseline to each post-booster vaccination assessment[a]
○ Based on protein specific T-cell response	• Percentage of cytokine producing S and N protein-specific T-cells compared to Baseline
○ Based on further parameters	• Additional assays to measure the immune response to Prime-2-CoV_Beta and immune response to the ORFV vector backbone

For all endpoints, Baseline will be defined as last measurement before Prime-2-CoV_Beta booster vaccination.

[a] With respective immunogenicity assessments.

8 Study design and design rationale

8.1 Overall study design

This is an open-label, first-in-human, dose-finding study to evaluate the safety and immunogenicity of a booster vaccination of Prime-2-CoV_Beta in healthy participants who had received the full course of vaccination, including booster vaccination (i.e., having received at least 3 doses) with a licensed messenger ribonucleic acid (mRNA)-based COVID-19 vaccine. Eligible participants will undergo baseline assessments and will receive 1 injection of Prime-2-CoV_Beta at Day 1. Participants will be followed up through 6 months post-booster vaccination. Follow-up visits will be performed at Days 4, 8, 15, 29, and Months 3 and 6, to assess the safety, tolerability, and immunogenicity of Prime-2-CoV_Beta. Additional safety and tolerability data will be assessed 1 day and 2 days after booster vaccination (Days 2 and 3) by telephone. A total of 60 participants is planned to be vaccinated in 5 cohorts of 12 participants each. Dose ranging of Prime-2-CoV_Beta will be done by dose escalation with doses ranging from $3x10^4$ plaque forming units (PFUs) up to $3x10^7$ PFUs (see Section 11.1.4).

Depending on safety and immunogenicity data obtained in an informal analysis after all participants have completed Day 15 (see 15.12) further cohorts may be opened (after approval of a respective protocol amendment).

All participants will record solicited AEs (local and systemic) in an electronic diary starting on the day of Prime-2-CoV_Beta booster vaccination throughout an additional 7 days (not counting vaccination day).

A safety review committee (SRC) will be established that will review safety data after the last participant of each of the Cohorts 1 to 5 has completed Day 8 and ad hoc in case holding rules apply. Further ad hoc SRC meetings may be scheduled upon request (e.g., for potential safety issues).

For vaccination sequence and dose escalation see Section 11.1.5.

8.2 Study design rationale

The first-in-human study with Prime-2-CoV_Beta will be a phase Ia, open-label, dose-finding study in healthy adults. Prime-2-CoV_Beta will be used as a booster vaccination, after participants have received the full vaccination, including booster vaccination (i.e., having received at least 3 doses) with an authorized mRNA-based COVID-19 vaccines. The proposed design is considered standard for this phase of development and type of product and provides the best option to evaluate the primary endpoint while ensuring the safety of participants.

Because seronegative participants will be difficult to recruit in Germany and to avoid withholding the currently recommended COVID-19 vaccination, a booster regimen was chosen

to assess the safety of Prime-2-CoV_Beta in this first-in-human study. To ensure comparability of cohorts and a homogenous population only participants having received mRNA-based COVID-19 vaccines are included. mRNA-based COVID-19 vaccines were chosen as they are the most frequently used vaccines in Germany, especially in the targeted study population.

This phase Ia study is uncontrolled since a control arm including participants receiving an mRNA-based COVID-19 vaccine booster or a placebo arm is not expected to significantly improve the understanding of safety. In addition, the dose-escalation design is an inherent control as the outcomes from the low dose group can be compared to the higher dose groups. An active comparator (i.e., a boost vaccination with an mRNA-based COVID-19 vaccine) is not planned since Prime-2-CoV_Beta is a vaccine using the Beta variant of SARS-CoV-2, while currently licensed mRNA-based COVID-19 vaccines comprise the original strain. Thus, also immunogenicity parameters (e.g., neutralizing antibodies) would not be easily comparable. In addition, the low numbers of subjects in each arm would not generate sufficient data to draw useful conclusions on the difference in safety or immunogenicity between the control arm and the ORFV arms. A side-by-side comparison is planned for later stages of clinical development.

To establish a safe and sufficiently immunogenic vaccine dose, the study uses a dose-escalation design that is commonly used in such studies. To minimize and assess potential risk to participants, the dose escalation will include a safety lead with 1 sentinel participant in all dose cohorts, who is followed up for at least 48 hours before further participants in that cohort are vaccinated. In addition, the next 2 participants in the dose cohort will be vaccinated at an interval of at least 4 hours. Remaining participants will be vaccinated at least 48 hours after Participant 3 and in 2 groups with an at least 48-hour interval between the vaccinations of these 2 groups (see Section 11.1.5). An SRC will review all safety data obtained after the last participant in each of the Cohorts 1 to 5 has completed 7 days of follow-up after the Prime-2-CoV_Beta booster vaccination and will give recommendations whether to proceed with dose escalation or not, and/or if any modifications to the study conduct should be implemented (see Section 11.1.5).

Participants exposed to sheep or goats are excluded as previous exposure to the wild-type Orf virus may have caused the production of neutralizing antibodies that reduce vaccine immunogenicity.

Solicited local and systemic reactions will be recorded over 7 days following the Prime-2-CoV_Beta booster vaccination using an e-diary. Because most adverse reactions (ARs) to vaccines occur within the first few days after vaccination, it is common practice and recommended by regulatory authorities [17] to collect solicited local and systemic symptoms for approximately 5 to 7 days after each dose. In line with these recommendations, an appropriate grading system is pre-defined in the protocol.

Staggered dosing, holding rules, the involvement of an SRC, and close safety monitoring including thorough cardiac monitoring (ECGs, cardiac biomarkers, echocardiography) after Prime-2-CoV_Beta booster vaccination are considered adequate to detect possible side effects of Prime-2-CoV_Beta early on, provide optimal safety to participants and ensure study integrity.

As recommended, [17] the immune response to each antigenic component of Prime-2-CoV_Beta is evaluated via the respective neutralizing, S and N-specific antibodies. In addition, the cell-mediated immunity of the immune response is assessed by quantifying antigen-specific T-cells. The immune response is assessed early after vaccination and up to 6 months to obtain long-term data.

A follow-up of 6 months is typical for a phase I vaccine study and is considered sufficient to elicit key safety information and immunogenicity data.

8.3 Justification of dose

The starting dose and escalation design are supported by nonclinical data, particularly data obtained in the non-human primates and hamster SARS-CoV-2 challenge studies, the pivotal repeat-dose toxicity study in rabbits and a telemetry study in non-human primates (NHP). The rabbits received an intramuscular (IM) dose of $5.7x10^7$ PFU (i.e., the full planned maximum human dose of at least $3x10^7$ PFU, and with an excess factor of 20 considering a human body weight of 50 kg and a rabbit body weight of 2.5 kg) 3 vaccinations every 2 weeks apart, i.e., Days 1, 15, 29. After finalization of the in-life phase, no acute ARs in in-life parameters or clinical pathology parameters were noted. Especially, immune stimulation by the vaccine in terms of hematology parameters, body temperature and cytokines were only minor and transient, indicating lack of a risk for cytokine release or enhanced disease and a low risk for side effects like fever, fatigue or pain at the injection site as commonly observed of other COVID-19 vaccines. No adverse effects were noted in rabbits, NHP, or hamsters. Effects observed could all be attributed to the mode of action of the vaccine. Up to the highest dose tested in all species (rabbit, NHP, rat, mouse, hamster, 5 - $5.7x10^7$ PFU) as well as after administration of 1.4 - $5x10^7$ PFU intravenously in rabbits, rats and mice, the vaccine was very well tolerated, demonstrating a good safety profile and resulting in a very good immune response.

The lowest dose tested in mice was $5x10^4$ PFU[1] applied IM on Day 1 and second time 3 weeks apart, leading to an immune response below (20 days after prime) or equal (7 days after second vaccination) to antibody titers of human convalescent serum (RBD-specific IgG). This dose led

[1] This titer corresponds to a titer as measured using a validated method at the Drug Substance manufacturer ABL. The nominal titer used in this study was $1x10^5$ PFU which was assessed by the UKT. The titer determined by ABL is about 50% of the titer determined by UKT; here only ABL-converted doses are used.

to nearly no cellular immune response in mice. Cytokines determined were hardly elevated 1 week after the second vaccination, whereas a clear cellular immune response was evident following doses of $5x10^5$, $5x10^6$ or $1.5x10^7$ PFUs. The animals received doses near the anticipated full human dose. The safety factor is 2400 between mice and humans, 24 between rabbits and humans, 17 between cynomolgus monkeys and humans, and 7.5 between rhesus monkeys and humans (considering a body weight of 60 kg for humans, 25 g for mice, 2.5 kg for rabbits, 3.5 kg for cynomolgus monkeys and 8 kg for rhesus monkeys) following the Guidance for Industry, Estimating the Maximum Safe Starting Dose in Initial Clinical Trials for Therapeutics in Adult Healthy Volunteers.

In this dose-finding study a starting dose of at least $3x10^4$ PFU is selected, as this was the minimal pharmacologic active dose in mice. This is justified by the excellent tolerability profile and very low physiological reactions in the various species and doses tested. Rabbits, NHP, rats, mice, and hamster received 1, 2, or 3 vaccinations with 5 - $5.7x10^7$ PFU, displaying factor 100 considering the intended starting dose and factor 750 for rhesus monkeys, 1700 for cynomolgus monkeys, 2400 for rabbits and 240.000 for mice considering the respective body weight. The vector has not been used in humans before and subjects are healthy volunteers, so the minimal anticipated biological effect level approach is selected to ensure safety of the participants ([18]). An escalation by factor 10 per dose cohort is justified by the excellent tolerability profile and very low physiological reactions in the various species and doses tested.

Up to the highest dose tested in the cardiovascular safety study in NHP ($4x10^7$ PFU), no effects were noted after single or repeated administration 4 weeks apart (see Figure 1).

8.4 Justification for post vaccination surveillance period

Nonclinical animal testing

Nonclinical animal testing was performed with Prime-2-CoV using an ORFV vector based recombinant vaccine encoding the N protein as well as the S protein. The latter was either derived from genes of the SARS-CoV-2 Wuhan wildtype strain (Prime-2-CoV), the SARS-CoV-2 Gamma strain (Prime-2-CoV_Gamma) or the SARS-CoV-2 Beta strain (Prime-2-CoV_Beta).

In none of the performed animal studies (mouse, rat, hamster, rabbits or NHP) any unphysiological clinical findings - apart from an isolated finding at the site of IM administration - were observed following the vaccinations with Prime-2-CoV and Prime2-CoV_Beta. No hints for any acute systemic ARs were given by existing animal data. The formulation contains no adjuvant, and hematology data sampled 3 days after the first application are nearly comparable to data of control-treated animals.

Rabbits in the repeat dose toxicity study were closely monitored for systemic and local reactions by clinical observations including injection site evaluation, body temperature measurement, and

body and food weight measurements after each intramuscular administration and additionally by determination of hematological and clinical chemistry parameters 3 days after the first vaccination to detect any potential early inflammatory reactions. None of the animals demonstrated any changes regarding the aforementioned parameters beyond the expectable local reactions upon administration of the vaccine. All parameters remained comparable to the control group, not showing any systemic activation of the immune system (no increased body temperature or signs of acute inflammation [C-reactive protein] were observed).

In this study, acute myocardial necrotic and inflammatory changes at or around the right atrioventricular groove region were observed in single rabbits 1 day after the 3rd vaccination, but in none of the recovery animals (2 weeks after the 3rd vaccination) and in none of the further nonclinical studies performed (in mouse, rat, hamster, NHP). Quantitative polymerase chain reaction and immunohistochemical evaluation demonstrated absence of Prime-2-CoV at the site of necrosis. Furthermore, no antibody-complexes (IgG, IgM) or increased levels of collagen I and II, indicating newly built collagen, were present at the site of lesion. From all parameters evaluated, it is concluded that the occurrence of this finding is of very acute nature, i.e., the histomorphological changes caused by the last treatment 24 hours before necropsy were transient in nature, as 14 days later no changes were observed. Central nervous system or blood parameters were neither affected by a single nor by up to 3 vaccinations in any of the species tested. A study focusing on the cardiovascular system was conducted in NHP following 2 vaccinations (administered 4 weeks apart). Electrocardiogram (ECG), blood pressure and respiratory parameters were not changed in the animals following both vaccinations (observation up to 96 hours after vaccination). Considering all investigations, the observed heart findings are most likely species specific and restricted to rabbits only. Based on additional study results, a direct cardiovascular effect in humans is assumed to be highly unlikely, and in the very improbable event of occurrence of any heart-related findings, a fast and complete recovery can be reasonably assumed. Anyhow, for risk mitigation reasons, ECG, echocardiography as well as measurements of troponin and creatine kinase-MB (CK-MB), as sensitive serum markers for cardiac damage, are included in the clinical study to ensure any cardiovascular effects are captured, allowing for timely and appropriate responses in any case. In addition, cardiac biomarkers abnormalities and/or the occurrence of clinical symptoms suggestive of cardiac injury will prompt further diagnostic work-up including ECG and/or more accurate and sensitive methods for cardiovascular disease diagnosis (e.g., cardiac magnetic resonance imaging [MRI]).

In rabbits, IM doses of up to 5.7×10^{7} PFU Prime-2-CoV or 6.1×10^{7} PFU Prime-2-CoV_Beta did not cause any swellings or reddening at the injection site. Two isolated cases of limping (one accompanied by a warming at the injection site, lasting for up to 3 days after the second vaccination) were assessed as most likely caused by IM administration rather than as local effects of the vaccine. In NHPs, a dose of 3×10^{7} PFU did not cause any swelling or reddening

at the site of injection neither after the 1st nor 2nd vaccination. A reddening in a single monkey in the control and low-dose Prime-2-CoV group ($1x10^6$ PFU) was observed after the 1st or the 2nd IM injection, respectively, both graded as mild. These effects developed 24 hours after treatment and lasted for a maximum of 48 hours following injection. As local effects were not observed in the high dose group and remained comparable in occurrence and intensity between control and ORFV-treatment groups, this was more likely a reaction to the injection procedure and the administered volume than reactivity to the vaccine itself. In mice, no local injection-site reactions after 1 to 3 repeated IM administrations have hitherto been noted when using Prime-2-CoV (up to $1x10^8$ PFU) or with any other ORFV-construct tested.

Biodistribution data for Prime-2-CoV indicate a rapid distribution and clearance from the site of injection. Following intramuscular injection, the distribution to tissues was limited, with quantifiable levels only 4 hours subsequent to administration in singe animals (heart, lung, spleen) apart from the injection site, where the vaccine could be quantified until 8 days post injection but showing a clear decline in levels determined. Remaining Prime-2-CoV 3 days after vaccination at the site of injection was at very low titers (in the range 10^2 to 10^3 PFU compared to levels of 10^6 PFU or 10^5 PFU 4 and 24 hours after injection, respectively). An occurrence of local reactions beyond 3 days following the vaccination is therefore highly unlikely.

Conclusion

Animal testing showed:

- no unphysiological clinical findings following vaccination except for mild local reactogenicity that developed within hours after administration
- no hints for systemic ARs following single and repeat vaccination IM in a dose range including the full human dose
- no alterations in hematology and clinical chemistry parameters following vaccination beyond minor expected reactions due to the immune-stimulating effect of the vaccine.

Clinical safety and vaccine reactogenicity

In absence of first-in-human data for the Prime-2-CoV_Beta vaccine, from a clinical standpoint and without any indication for any concerning side effects observed in comprehensive animal studies, most importantly from NHPs (but also in mice, hamsters and rabbits), it is most likely that AEs will remain restricted to the known and well-established reactogenicity of vaccines. Single findings of acute reversible cardial necrosis observed in rabbits after a 3rd dose remained species specific and detailed investigations of the issue could not substantiate any risk in more appropriate animal models (NHP) or after 1 or 2 injections of the vaccine, respectively.

It can be anticipated that injection site reactions including pain, redness, swelling and indurations may be observed in humans, but such reactions following vaccination are very

common and generally mild, also with vector vaccines. [19] Respective findings are usually observed relatively soon after vaccination and self-limiting within few days; a notion also supported by the available animal data for Prime-2-CoV_Beta.

Common systemic AEs usually evidenced in conjunction with vaccine administration are fever, headache, myalgia, arthralgia, and fatigue, which also usually start hours after vaccine administration but remain mild and self-limiting. [20]

Considering recent data self-reported from 2000 subjects from the general population who received COVID-19 vaccines, overall reactions to vaccines were predominantly mild and transient but showed increased local reactogenicity for mRNA-based vaccines in contrast to approved vector vaccines. [21]

Further, AEs such as local vesicular skin lesions and arthritis as observed with replication-competent virus vector vaccines can be excluded for Prime-2-CoV_Beta, which does not replicate in humans after vaccination.

In addition, we know that in general severe AEs such as anaphylaxis are very rare events after vaccinations (usually observed at a frequency of 1:1 million administered doses). [22] This also applies to vector vaccine development and e.g., in a comparable setting 2 SAE reports of anaphylaxis across a program with over 20,000 vaccinated subjects were observed. [19] Such events usually occur in a very short time after (re-) administration of the vaccines. Recently, also in mRNA-based COVID-19 vaccines anaphylactic reactions to an excipient were described also at a comparably low frequency, with all events occurring within minutes after administration and controllable by available drug treatments. [23]

Nevertheless, as a precautionary measure against any unexpected immediate reaction (e.g., exaggerated immune response or allergic reaction) to the vaccine or components contained in the formulation, sentinel participants will be followed up for at least 48 hours after vaccination for each escalating dose group, and staggered dosing applies for subsequent participants. Further, all study participants will stay at least 4 hours at the study site for surveillance.

Conclusion

Based on these considerations and comprehensive nonclinical data from the investigational product (IP) and comparable products, it can be assumed that a benign safety profile will also prevail with the Prime-2-CoV_Beta vaccine in humans. Most importantly there are no signals of concern that need further addressing or any critical issues at this stage of development. However, extensive precautionary measures are implemented.

Therefore, the suggested surveillance of 1 sentinel participant in each escalating dose group for 48 hours and the staggered vaccination of the next two subsequent participants with intervals of at least 4 hours (between Participant 2 and 3) or at least 48 hours (between Participant 3 and Participants 4-7, and between Participants 4-7 and Participants 8-12, see Figure 1) between

administrations to account for any acute reactions (anaphylaxis, acute systemic reactions) as well as surveillance for all study participants encompassing at least 4 hours at the site after vaccinations is deemed rather conservative and on the side of caution. As a risk mitigation strategy to the finding of isolated cardial necrosis witnessed only in rabbits and only after 3 vaccinations within 4 weeks, analyses of sensitive cardial serum markers troponin and CK-MB as well as ECGs and echocardiography were included at appropriate time points in the study protocol, enabling the early detection of any potential issues and a swift response. In addition, in case of cardiac biomarker or ECG abnormalities, or whenever clinical symptoms suggestive of cardiac injury occur further diagnostic work-up including more accurate and sensitive methods for cardiovascular disease diagnosis (e.g., cardiac MRI) must be scheduled. Participants with such symptoms and/or abnormalities should be referred to a specialist and/or, preferentially, admitted to hospital for cardiac monitoring and further medical management and the sponsor should be informed. Further the SRC will have data from all study participants over a minimum of 7 days available before approving the next dose group to be entered in the study.

It is assumed that this approach safeguards the health and protects study participants against any potentially arising issues that are very unlikely but must be controlled and that such issues are adequately controlled and subsequently addressed by the proposed study design, halting rules, and SRC involvement.

8.5 Risk-benefit assessment

Risks

Unexpected adverse reactions: Although in nonclinical studies the vaccine candidate was safe and well tolerated, it has not yet been administered to humans and no safety data in humans are yet available. Thus, unexpected ARs may occur (for safety in nonclinical studies see Section 8.4).

Also, there is no previous experience in humans with the ORFV because no ORFV-based vaccine has yet been approved for use in humans. However, the vector used is not able to replicate *in vivo* and is assigned to biological safety level 1 and therefore is considered to have a favorable risk profile.

Frequently observed ARs after IM vaccination include **local** (pain, tenderness, swelling, redness) and **systemic reactions** (fever, irritability, malaise, muscle pain, headache, loss of appetite). These reactions are generally mild or moderate, occur typically within a day or 2 after vaccination, and are short-lived (1 to few days). In addition, allergic reactions may occur but are uncommon and anaphylaxis following vaccination is rare, occurring at a rate of about 1 per 1 million doses for many vaccines [24] (also see Section 8.4).

Cardiac toxicity: Acute myocardial necrotic and inflammatory changes at or around the right atrioventricular groove region were observed in single rabbits 1 day after a 3rd vaccination with

Prime-2-CoV_Beta. Although these heart findings are considered species specific, occurrence of such events in humans cannot be completely ruled out. However, respective risk minimization procedures have been implemented (see below) and the risk of cardiac events is considered very low. Further, only a single vaccination with Prime-2-CoV_Beta will be performed in this study.

Theoretical risk of disease enhancement: Nonclinical data from SARS-CoV-2 and other respiratory viruses suggest that anti-SARS-CoV-2 antibodies might exacerbate COVID-19 through ADE, i.e., the induction of antibodies not neutralizing but enhancing SARS-CoV-2 infection. [25] However, in nonclinical studies with the vaccine candidate no ADE was observed in mice and nonhuman primates. Moreover, the response to Prime-2-CoV_Beta was Th1-dominated and thus, further limits the risk of potential ADE.

Venipuncture: Complications that can arise from venipuncture include bleeding, bruising, fainting, infections, and hematoma formation.

Vaccination efficacy: Prime-2-CoV_Beta has not yet been tested in humans. Although an adequate immune response to Prime-2-CoV_Beta was observed in non-clinical studies, an equivalent immune response in humans to protect against COVID-19 (obtained with currently available and licensed COVID-19 vaccines) cannot be guaranteed, especially with low doses. In addition, Prime-2-CoV_Beta targets the SARS-CoV-2 beta strain and not the current VoC. To avoid unnecessarily exposing participants to the risks associated with SARS-CoV-2 infections only participants who have received a full course of vaccination including booster vaccination (the currently recommended standard vaccination against COVID-19, at least 3 vaccinations in total) with a licensed and effective mRNA-based vaccine are allowed to be included.

Risk management

Risk minimization procedures are implemented for this study to minimize and assess potential risks to participants. These include, but are not limited to:

- Enrollment of participants who have received the full course of vaccination, including booster vaccination (i.e., have received at least 3 doses).
- Use of a dose escalation design with a safety lead-in in each escalating dose cohort (i.e., 1 sentinel participant followed up for at least 48 hours, before the next 2 participants are vaccinated with an interval of at least 4 hours).
- An SRC will be established that will review safety data, and holding rules are defined.
- A diary will be used to closely monitor potential ARs to the IP within 7 days after the vaccination day. In addition, each participant will be monitored for at least 4 hours after Prime-2-CoV_Beta booster vaccination at the center.

- ECG parameters and serum cardiac markers i.e., troponin and CK-MB will be regularly assessed and provided to the SRC. In addition, an unscheduled ECG will be performed whenever clinical symptoms suggestive of cardiac injury occur, or in case of cardiac biomarker abnormalities.
- An echocardiography will be performed 3 months after the Prime-2-CoV_Beta vaccination.
- In case of cardiac biomarker abnormalities and whenever symptoms suggestive of cardiac injury occur further diagnostic work-up including more accurate and sensitive methods for cardiovascular disease diagnosis (e.g., cardiac MRI) will be performed.
- Participants with a history of cardiovascular disease are excluded, as are participants who experienced myocarditis after an mRNA vaccination
- Participants who have evidence of an active COVID-19 infection and are considered at high risk of severe COVID-19 are excluded to reduce any risk of vaccine-associated enhanced respiratory disease.
- All participants are followed up for AESIs including AEs listed as holding rules, AEs with suspected immune-mediated etiology, AEs relevant to SARS-CoV-2 vaccine development, or the target disease (see Section 14.1.2).
- Venipuncture will only be performed by qualified staff.

Benefits

Participants do not have any guaranteed benefit. However, they may receive a potentially effective Prime-2-CoV_Beta booster vaccine against COVID-19 and will undergo regular antibody testing.

Prime-2-CoV_Beta contains the mutation E484K which is a known escape mutation resulting in reduced neutralization by Spike-specific antibodies. As this mutation is present in the VoCs Beta, Gamma and Omicron, Prime-2-CoV_Beta might be potentially effective against these VoCs. In addition, Prime-2-CoV_Beta encodes the well conserved N protein in addition to the S protein. This should induce a broader humoral and cellular immune response potentially resulting in improved affinity maturation of S-specific antibodies because of CD4 T-cell help.

Further, participants will support current research to develop a safe and effective vaccine against COVID-19, urgently needed to contain this pandemic.

Overall risk-benefit conclusion

Considering the safety data available, the risk minimization measures and the medical need for vaccines against COVID-19, the risk-benefit evaluation is considered favorable.

9 Study population

9.1 Sample size

A total of 60 participants are planned to be treated in 5 cohorts of 12 participants each. Depending on safety and immunogenicity data obtained in an informal analysis after all participants have completed Day 15 further cohorts may be opened. See also Section 15.1.

Efforts will be made to ensure good representation of both men and women to ensure that sex distribution in the group of participants is appropriate to identify possible sex differences in the safety or immune response to the IP.

Participants are considered enrolled at the time of signing the consent form.

9.2 Inclusion criteria

Participants meeting the following criteria will be considered for inclusion into the study:

1. Healthy adult men or women aged 18 to 55 years
2. Full course of vaccination, including booster vaccination (i.e., having received at least 3 doses) with a licensed mRNA COVID-19 vaccine, with the last dose being administered at least 10 weeks before Day 1 as documented in a respective vaccination certificate
3. Able to understand the participant information and providing written informed consent
4. Body mass index of 18.5 to 30.0 kg/m² and weight > 50 kg at Screening
5. Women of childbearing potential must:
   a. have a negative pregnancy test at Screening (blood) and at Day 1 (urine)
   b. agree to use, and be able to comply with, highly effective measures of contraception without interruption, from 14 days before Prime-2-CoV_Beta booster vaccination until the end of the study.

      A highly effective method of contraception or birth control (failure rate less than 1% per year when used consistently and correctly) for this study: combined (estrogen and progestogen containing) hormonal contraception associated with inhibition of ovulation (oral, intravaginal, transdermal, injectable), progestogen-only hormonal contraception associated with inhibition of ovulation (oral, injectable, implantable), intrauterine device, intrauterine hormone-releasing system, bilateral tubal occlusion, sexual abstinence or vasectomized sexual partner. Abstinence is only acceptable as true abstinence when this is in line with the preferred and usual lifestyle of the participant (abstinent on a long-term and persistent basis). The reliability of sexual abstinence needs to be evaluated in relation to the duration of the clinical study and

the preferred and usual lifestyle of the participant. (Periodic abstinence [e.g., calendar, ovulation, symptothermal, post-ovulation methods and withdrawal] are not acceptable methods of contraception.)

Postmenopausal (no menses for at least 1 year without alternative medical cause) or surgically sterile women (tubal ligation, hysterectomy or bilateral oophorectomy) may be enrolled.

6. Male participants must agree not to intend to father a child or to donate sperm starting at Screening, throughout the clinical study. Male participants must also
   a. abstain from sexual intercourse with a female partner (acceptable only if it is the participant’s usual form of birth control/lifestyle choice: abstinent on a long-term and persistent basis). The reliability of sexual abstinence needs to be evaluated in relation to the duration of the clinical study and the preferred and usual lifestyle of the participant or
   b. use adequate barrier contraception (male condom) during treatment with the investigational product until the end of the study, and
   c. ensure that, if they have a female partner of childbearing potential, the partner uses a highly effective contraceptive method as outlined in inclusion criterion number 5
   d. use condoms during the entire study if they have a pregnant partner, to avoid exposure of the fetus to the investigational product
7. Willing and able to comply with all study procedures based on the investigator’s judgment

9.3 Exclusion criteria

A participant will not be eligible for inclusion if any of the following criteria applies:

Previous and concomitant therapy

1. Receipt of any vaccine (licensed or investigational) from 4 weeks before Prime-2-CoV_Beta booster vaccination or anticipated vaccination during the study until 6 weeks after the Prime-2-CoV_Beta booster vaccination
2. Previous vaccination against COVID-19 with vaccines (licensed or investigational) other than mRNA-based vaccines (licensed or investigational)
3. Current or previous treatment with another investigational drug and/or medical device (within 30 days of enrollment or 5 half-lives of that investigational drug)
4. Administration of immunoglobulins or any blood products within 2 months of Prime-2-CoV_Beta booster vaccination

5. Chronic administration of medication associated with impaired immune responsiveness as judged by the investigator (including, but not limited to: immunosuppressive therapy, systemic corticosteroids exceeding 10 mg/day of prednisone equivalent, allergy shots for hypo-sensitization, immunoglobulin, interferon, immunomodulators, cytotoxic drugs, or other similar or toxic drugs) within 2 months before the Prime-2-CoV_Beta booster vaccination (Day 1). Inhaled/nebulized, intra-articular, intrabursal, or topical (skin or eyes) corticosteroids are permitted.

Previous and concomitant medical condition

6. Active SARS-CoV-2 infection, confirmed by a commercially available SARS-CoV-2 rapid antigen test at Day 1, or currently on quarantine
7. *Deleted in protocol Version 5.0*
8. Known history of severe adverse reactions to any vaccine and/or severe allergic reactions to any component of the study vaccine, to any drug, or to any other exposure
9. Known history of angioedema
10. Pregnant or lactating women
11. Any confirmed or suspected immunosuppressive or immunodeficient condition
12. Known history of Guillain-Barré Syndrome
13. Known infection with human immunodeficiency virus, hepatitis C virus or hepatitis B virus
14. Active cancer (malignancy) within 5 years before Day 1 (except for adequately treated non-melanomatous skin carcinoma, at the discretion of the investigator)
15. Moderate or severe illness and/or fever > 38.0°C within 1 week before Prime-2-CoV_Beta booster vaccination
16. Any clinically significant health problem (medical history and physical examination) or clinically significantly abnormal finding in biochemistry and/or hematology blood tests, urinalysis, or electrocardiogram at Screening according to the investigator’s opinion
17. Current or history of cardiovascular disease or structural cardiac disease (including chronic or congenital heart conditions, such as chronic hypertension, coronary heart disease, myocardial infarction and arrhythmias, hypertrophic cardiomyopathy, as well as a history of myocarditis after mRNA vaccinations)
18. History of mRNA vaccination-associated AEs that were in nature and severity beyond the common AEs expected

19. Current or history of gastrointestinal disease, liver disease, renal disease or endocrine disorders, (including diabetes) and neurological illness (excluding migraine), when judged as clinically significant according to the investigator's opinion
20. Current or history of chronic respiratory diseases, including mild asthma treated by on-demand medication (resolved childhood asthma is allowed)
21. Current or history of alcohol and/or drug abuse within the last 6 months before Day 1

Previous and concomitant clinical study experience

22. Current participation in another study or previous enrollment in this clinical study

Other exclusion criteria

23. Investigator or employee of the study group or sponsor with direct involvement in the proposed study or relatives of the research staff with direct involvement in the proposed study
24. Prolonged exposure to sheep or goats (e.g., shepherds, sheep farmer)

9.4 Criteria for delaying vaccination

Participants meeting any of the following criteria may be delayed for vaccination

- Current febrile illness (body temperature ≥ 38 °C) or respiratory and/or COVID-19 symptoms (i.e., cough, sore throat, shortness of breath, wheezing, sputum production, loss of taste and/or smell) within 1 week before Prime-2-CoV_Beta booster vaccination
- Other acute illness within 2 days before vaccination (e.g., gastroenteritis, migraine, urinary tract infection, injury) with symptoms that may impact the safety and tolerability assessment of the IP as judged by the investigator

In both cases, the participant may be vaccinated once symptoms have been resolved for at least 3 days.

9.5 Re-screening

A participant can be re-screened based on the judgment of the investigator in case of a transient disease status (e.g., participant complains of a temporary "cold or fever"), or if a protocol eligibility criterion that is not met at the initial screening, will be met at re-screening later (e.g., prohibited medication).

10 Randomization, blinding and unblinding procedures

10.1 Randomization and blinding

Randomization and blinding are not applicable as this is an open-label study with sequential cohort enrollment.

10.2 Participant identification

A unique participant number will be assigned to each participant enrolled in the study. The participant number will be assigned sequentially by the eCRF.

11 Treatments

11.1 Investigational products

All medications supplied by the sponsor will be manufactured, tested, and released according to current Good Manufacturing Practice guidelines. Applicable regulations on genetically modified organisms will be followed to minimize dissemination of recombinant viral vector-based vaccine virus into the environment.

11.1.1 Test product

Name:	Prime-2-CoV_Beta
Manufacturer:	Nova Laboratories, Martin House, Gloucester Crescent, Wigston, Leicester, UK
Description:	ORFV vector based recombinant vaccine encoding the following genes of SARS-CoV-2, Beta variant:<br>• **The S protein** containing the following mutations: D614G (change from an aspartate to a glycine residue at position 614, the most dominant form of SARS-CoV 2 worldwide), K986P and V987P (change from lysine or valine, respectively to proline, proline stabilization), and AA682-685: RRAR ⇒ GSAS (furin cleavage site mutation with R = arginine, A = alanine, G = glycine, S= serine), and K417N, E484K, N501Y<br>• The structural **N protein**
Formulation:	Solution of at least 3 x 10^5 PFUs (low dose) or at least 3 x 10^7 PFUs (high dose) in ([redacted]% (w/v) [redacted] and [redacted]% (w/v) sucrose in [redacted] mM [redacted] pH [redacted], and [redacted] mM [redacted]).<br>To obtain the appropriate concentration the respective stock solution must be diluted with isotonic saline solution according to the IP manual.
Appearance:	Clear, colorless solution
Application:	IM injection (1.0 mL) into the deltoid muscle
Frequency:	1 injection
Dose strength:	See Table 2
Packaging:	Clear glass vial (2R) with bromobutyl stopper, sealed with an aluminium seal and flip-off overcap
Content:	Each vial contains 1.17 mL of solution, enabling a minimum extractable volume of 1.0 mL for injection

11.1.2 Description, packaging and labeling

All IP will be packed and labelled according to applicable regulatory requirements. Labels will include at least the following information: Sponsor, route of administration, study code, participant number (only placeholder), batch number, expiry date, concentration, instructions for storage, and that it is for clinical study use only.

11.1.3 Storage and stability

The investigator is responsible for safe and proper handling and storage of the IP at the study center. The IP must be stored in a locked facility and monitored (manual or automated recording) area in accordance with the labeled storage conditions and with access limited to the investigator and authorized personnel. The investigator must ensure that the IP is administered only to the participants enrolled in this study.

The IP must be stored at - 20 °C ± 10 °C and protected from light.

11.1.4 Treatment dose, dose cohorts, and administration

The IP is planned to be administered in 5 cohorts of 12 participants. The dose of Prime-2-CoV_Beta will be sequentially increased in each cohort starting at $3x10^4$ PFUs up to $3x10^7$ PFUs (see Table 2). Depending on safety and immunogenicity data obtained in an informal analysis after all participants have completed Day 15 further cohorts may be opened (after approval of a respective protocol amendment).

Participants in each cohort will receive 1 dose of IP (1.0 mL) on Day 1. The IP should be administered IM into the deltoid muscle by an appropriately qualified member of the study staff.

All participants will be observed for at least 4 hours after vaccination at the study center. Vital signs (Section 14.6) will be checked before discharge from the center. In case of values outside the normal range, vital sign measurements will be repeated after 15 to 30 minutes or later if deemed necessary by the investigator. The participant should not be discharged until vital signs have returned to pre-vaccination levels.

During administration, appropriate medication and resuscitation equipment must be available for the management of acute hypersensitivity reactions.

Table 2: Dose cohorts

Cohort	Dose (PFU)	Number of vaccinations	Number of participants
1	$3x10^4$	1	12
2	$3x10^5$	1	12
3	$3x10^6$	1	12
4	$1.5x10^7$	1	12
5	$3x10^7$	1	12
**All**			**60**

PFU = plaque forming units

Details on how to prepare the IP for administration will be provided to the center in the IP manual.

11.1.5 Sequence of vaccination and dose escalation

The vaccination schedule is depicted in Figure 1.

Cohorts 1 to 5 will include a safety lead with 1 sentinel participant. This sentinel participant will stay at the center for the first 4 hours and will be followed up for at least an additional 44 hours after Prime-2-CoV_Beta booster vaccination. One day after vaccination and at the end of the 48-hour observation period, center personnel will contact the participant by telephone to obtain information on solicited and unsolicited AEs and other safety issues. If no safety issues occurred within the on-site monitoring period as assessed by the investigator and solicited during the telephone visits at Days 2 and 3, the next 2 participants (Participant 2 and 3) in that dose cohort will be vaccinated with an interval of at least 4 hours between vaccinations. After an additional 48-hour observation period and assuming no safety issues were identified in these 2 participants (during the on-site monitoring or phone visits), an additional 4 participants will be vaccinated with at least 30 minutes between vaccinations. After a further 48-hour observation period, and assuming that no safety problems were noted in these 4 participants (during the on-site monitoring or phone visits), the remaining participants in the dosing group will be vaccinated with an interval of at least 30 minutes between vaccinations. Each participant will be observed for at least 4 hours at the study center after Prime-2-CoV_Beta booster vaccination.

After the last participant of each of the Cohorts 1 to 4 has completed 7 days of follow up after the Prime-2-CoV_Beta booster vaccination, all safety data available including full cardiac assessments and complete safety laboratory will be reviewed by the SRC. The independent members of the SRC will then provide recommendations whether to proceed with dose escalation or not. The SRC will also review Cohort 5 safety data after all Cohort 5 participants have completed 7 days of follow up and provide recommendations whether any modifications to study conduct should be implemented.

The SRC will specifically evaluate AEs ≥Grade 3 and SAEs considered related to Prime-2-CoV_Beta. Abnormalities in laboratory values including troponin, CK-MB, and coagulation markers, deemed clinically significant by the investigator will be thoroughly reviewed as will any cardiac assessment (ECG, vital sings, and others if available). All reports from additional cardiac and safety assessments available at the respective meeting will be reviewed and considered. Any clinically unacceptable finding for which a relation to Prime-2-CoV_Beta cannot be excluded will prevent dose escalation. The independent members of the SRC will make the sole decision on the final recommendation for dose escalation or other necessary study modifications. Data listings provided to the SRC will be described in the SRC charter.

11.1.6 Vaccination holding rules

Any AEs meeting any one of the following criteria (based on review of AE data and diary data) will lead to a pause of further vaccinations. These data will be monitored on an ongoing basis by the investigator (or medically qualified designee), the SRC, and the sponsor.

1. Any participant experiences treatment-related ulceration, abscess, or necrosis at the injection site
2. Any participant experiences laryngospasm, bronchospasm, or anaphylaxis within 24 hours after IP administration
3. Any participant experiences a treatment-related SAE within 7 days after IP administration
4. Any participant experiences a treatment-related extensive dermal allergic reaction such as generalized urticaria (defined as occurring at 3 or more body parts) within 72 hours after IP administration
5. 2 or more participants experience the same ≥ Grade 3, treatment-related AE (solicited or unsolicited and/or clinical laboratory abnormality), in the same preferred terms based on the medical dictionary for regulatory activities (MedDRA) coding, which lasted at least 48 hours and occurred within 28 days after IP administration
6. Any participant experiences a Grade 4 treatment-related, local or systemic event within 7 days after IP administration
7. Any participant experiences a clinically significant increase in CK-MB and/or troponin, a respective abnormal finding in the ECG, and/or relevant clinical symptoms suggestive of cardiac injury within 28 days after IP administration

All events as defined above will be considered AESIs. For reporting rules see Section 14.1.2. If one of the above criteria applies, further vaccinations in any group will be paused until an ad hoc safety review by the SRC has been performed and conclusions and potential suggested measures were communicated to the sponsor. If considered appropriate according to the safety review, vaccination will be resumed. The safety review will consider e.g., the relationship of

the AE or SAE to the vaccine, new and relevant safety information from ongoing research programs with Prime-2-CoV_Beta, and additional screening tests to identify those participants who may develop similar symptoms.

All other study activities, including ongoing data entry, reporting of AEs, participant diary completion, blood sample collection, and participant follow-up of participants who have already been vaccinated will continue during the pause.

11.1.7 Investigational product accountability and compliance

IP must not be used outside the context of this study protocol. The investigator or authorized staff must document the receipt, storage, administration, and return of all IP received during this study. At the end of the study, all remaining IP must remain with or be returned to the sponsor or sponsor's designee for an accurate accounting of all delivered and returned IP.

Records on receipt, storage, use, return, loss, or other disposition of IP must be maintained. The investigator, his/her delegate, or, if applicable, pharmacist must sign the receipt forms. Records on IP delivery to the center, the inventory at the center, the use by each subject, and the return to the sponsor or sponsor's designee must be maintained by the investigator and/or a pharmacist or another appropriately trained individual at the study center. These records will include dates, quantities, batch numbers, and the participant numbers assigned to the IP and participants. The investigators must maintain records documenting that the participants were provided with the doses specified in the protocol. Furthermore, they should reconcile all IP received from the sponsor. It is the responsibility of the investigator to give reasons for any discrepancies in IP accountability. Forms will be provided to enhance IP accountability.

Following authorization by study management, IP may be destroyed at the study center following final accountability and in accordance with applicable regulations. Documentation of destruction is required. Alternatively, all unused products will be collected by the monitor or designee and returned to the sponsor or sponsor's designee for destruction.

All vaccinations will be administered by a qualified study staff. Administration and date, time, and location of injection will be entered into the eCRF. Because IP will not be handed out to participants, no further measures to assess compliance will be undertaken.

11.2 Previous and concomitant medication

All previous medication including previous vaccinations administered within 1 month before providing informed consent must be documented in the corresponding section of the eCRF. The date of the previous (at least 3) vaccinations with the licensed mRNA-based vaccine will also be recorded. Other relevant previous medication as judged by the investigator should also be documented.

All treatments being taken by the participants at entry into the study and all treatments given in addition to the IP during the study are regarded as concomitant treatments and must be documented in the eCRF.

The following medications and therapies are prohibited:

- Chronic administration of medication associated with impaired immune responsiveness (including, but not limited to: immunosuppressive therapy, systemic corticosteroids exceeding 10 mg/day of prednisone equivalent, currently undergoing allergy shots for hypo-sensitization, immunoglobulin, interferon, immunomodulators, cytotoxic drugs, or other similar or toxic drugs) within 2 months before Prime-2-CoV_Beta booster vaccination (Day 1) until 3 months after Prime-2-CoV_Beta booster vaccination. Inhaled/nebulized, intra-articular, intrabursal, or topical (skin or eyes) corticosteroids are permitted.
- Receipt of any vaccine (licensed or investigational) other than COVID-19 vaccines from 4 weeks before Prime-2-CoV_Beta booster vaccination through 6 weeks after the Prime-2-CoV_Beta booster vaccination.
- Receipt of any other, non-study COVID-19 vaccine at any time before or during the study, except for mRNA-based COVID-19 vaccines (licensed or investigational), that are allowed ≥10 weeks before Day 1.
- Receipt of immunoglobulins or any blood product blood/plasma products within 2 months of Prime-2-CoV_Beta booster vaccination through conclusion of the study.

12 Study schedule

12.1 Study conduct

An overview on study conduct is provided in the schedule of assessments (see Section 1, Table 1).

At each study visit and telephone visit participants will be reminded to contact the center immediately, if

- the participant experiences any local or systemic reactions or another medical event
- any medically attended event (e.g., doctor’s visit, emergency room visit) or hospitalization occurs
- the participant experiences any symptoms suggestive of COVID-19 or had a positive SARS-CoV-2 test performed

12.2 Screening (Days -22 to Day 1)

Participants for whom written informed consent (for consent procedures see Section 17.3) was obtained will undergo the assessments shown in Table 1. Screening assessment can occur up to 22 days before Prime-2-CoV_Beta booster vaccinations. Participants will be screened for eligibility based on the study's inclusion and exclusion criteria. If eligible, a Day 1 visit will be scheduled for the participant to receive the vaccine and subsequent follow-up or if possible, the screening and Day 1 visit maybe performed the same day. However, safety laboratory results and results of a pregnancy test not older than 24 hours (for women of childbearing potential) must be available before vaccine administration to confirm eligibility.

12.3 Day 1 (Baseline)

Assessments outlined in Table 1 will be performed. If the Day 1 visit is performed on the same day as the screening visit, assessments scheduled for both visits must be performed only once. Before Prime-2-CoV_Beta booster vaccination, an acute infection with SARS-CoV-2 must be excluded by a rapid antigen test. If all eligibility criteria are met, the participant will be vaccinated. Participants are considered enrolled at the time of signing the informed consent.

After vaccinations, participants will stay at the study center for at least 4 hours for observation, in case of immediate ARs.

Participants will receive the following:

- Participant card with an emergency 24-hour telephone number to contact the center, if needed
- Instructions on how to complete the e-diary (for more details see Section 14.4)
- Measuring tape to measure local reactions at the injection site and a thermometer for recording daily temperatures and instructions on their use

12.4 Subsequent clinic visits

Further visits will be performed as outlined in the schedule of assessments (Table 1) with respective time windows as indicated. Assessments will include vital signs, ECGs, echocardiography, review of e-diary data, blood tests for safety laboratory and immunology, and local and systemic AEs and SAEs.

The next visits will be scheduled.

12.5 Telephone visits

Center staff should contact the participant 1 and 2 day(s) after Prime-2-CoV_Beta booster vaccination to obtain or discuss the following information:

- Solicited and unsolicited AEs and other AEs (SAEs, medically attended AEs)
- Concomitant medications

If the participant reports any local or systemic AEs of potential concern these should be followed-up either by (a) telephone call(s) or by an unscheduled clinic visit based on the judgment of the investigator.

12.6 Unscheduled visits

Unscheduled visits may be performed at any time during the clinical study and will generally include the assessments listed in Table 1. Depending on the reason for the unscheduled visit, further appropriate assessments maybe done at the investigator's discretion. Results of the assessments and any change in concomitant medications will be recorded in the eCRF. After an unscheduled visit, the regular scheduled visits must continue according to Table 1.

12.7 End-of-study visit and early termination

The end-of-study (EoS) visit will be performed 6 months after Prime-2-CoV_Beta booster vaccination including the assessments outlined in Table 1.

In case a participant prematurely discontinues the clinical study (for reasons see Section 16), whenever possible, the participant will be asked to complete an early termination visit at which all assessments normally performed at the EoS visit will be performed.

In case of premature withdrawal from the study or IP, reasons, circumstances, and findings will be fully described on the corresponding page in the eCRF respecting the participant's rights.

Study staff will contact participants who fail to return for a final assessment and will ask these participants to come for a final visit. The status of participant who fail to complete the final assessments will be documented in the eCRF.

12.8 Medical care upon termination of the clinical study

After the final visit participants will not receive any further study-specific treatment.

12.9 Restrictions due to pandemics

During, e.g., governmental imposed, curfews in response to COVID-19 spread or other pandemic periods, the participant may be unable to attend scheduled on-site visits for their own safety or the safety of others, or due to restrictions on leaving home, or restrictions on social contacts.

If, due to restrictions, on-site visits are no longer possible, the visits will be performed by telephone. During the telephone visits, at least the following will be recorded:

- Any changes in health (new unsolicited AEs and/or the status of reported unsolicited AEs)
- Changes in concomitant medications taken by the participant

Also, study visits may be carried out as home visits, if possible and desired by the participant. The participant's consent to these visits will be covered in the informed consent form (ICF). Home visits are only to be performed by members of the study team (e.g., study nurses or flying study nurses). If needed ECGs and blood collections can also be performed during these home visits.

All general and clinic-specific applicable, e.g., COVID-19 pandemic, rules for performing the required examinations must be considered.

If the participant withdraws his/her consent for participation in the study, the investigator will contact him/her by telephone to assess the health condition.

13 Immunogenicity assessments

Serum samples will be obtained for immunogenicity testing at the visits specified in Table 1. Details for the collection, processing, storage, and shipping of blood samples for serological and cellular immunology assays are outlined in respective laboratory manuals. All assays used are qualified and, when available, validated.

The following assays will be performed:

Serological immunogenicity assays will include but are not limited to:

- IgG enzyme-linked immunosorbent assay to SARS-CoV-2 S1-, RBD-, N-protein
- Neutralization assay using SARS-CoV-2 Wuhan wildtype strain
- Neutralization assay using the following VoCs: SARS-CoV-2_Beta, SARS-CoV-2_Delta, SARS-CoV-2_Omicron
- Neutralization assay to evaluate neutralizing antibody responses against the ORFV vector

Cellular immunology assays:

- SARS-CoV-2 S and -, N protein-specific T-cells isolated from human peripheral blood mononuclear cells (PBMCs) will be assessed by enzyme-linked immuno-spot assay.

In addition, exploratory analyses to measure the immune response to Prime-2-CoV_Beta and immune response to the ORFV vector backbone will be performed.

Participants will be asked to provide consent to use stored blood samples for additional tests to obtain further data on the immune response to Prime-2-CoV_Beta and to support further vaccine development, if not prohibited by local requirements or ethics committee regulations.

14 Safety assessments and procedures

14.1 Adverse events

14.1.1 Definitions

An **AE** is any untoward medical occurrence in a participant administered a medicinal product and which does not necessarily have to have a causal relationship with the treatment. An AE can therefore be any unfavorable and unintended sign (including an abnormal laboratory finding, for example), symptom, or disease temporally associated with the use of a medicinal product, whether or not considered related to the IP.

Any worsening in severity or frequency of a concomitant disease or any new disease diagnosed in the study must be documented as an AE.

Clinically significant abnormal laboratory findings are considered AEs; however, abnormal laboratory findings may not be considered AEs, if there is no change compared to baseline values.

A surgery or procedure scheduled to occur during the study will not be considered an AE, if the surgery or procedure will be performed for a pre-existing condition and the surgery or procedure was planned before study entry. However, if the pre-existing condition deteriorates unexpectedly during the study (e.g., surgery performed earlier than planned), then the deterioration of the condition for which the surgery or procedure is being done will be considered an AE.

Unsolicited AEs of all severities will be reported from the time of signing the informed consent through EoS. Unsolicited AEs that occur between signing the informed consent and the time when that participant is administered IP are defined as "**pre-treatment-emergent**" events.

All unsolicited AEs which occur after the administration of IP are defined as **TEAEs**.

Procedures for solicited AEs are described in Section 14.4.

An **SAE** is any AE occurring at any dose that:

- results in death
- is life-threatening
- requires inpatient hospitalization or prolongation of existing hospitalization
- results in persistent or significant disability/incapacity
- is a congenital anomaly/birth defect
- is an important medical event/other.

Important medical events that may not have resulted in death, were not life-threatening, or did not require hospitalization are considered an SAE when, based upon appropriate medical judgment, they may have jeopardized the participant and required medical or surgical intervention to prevent one of the outcomes listed in this definition. Examples of such events are intensive treatment in an emergency room or at home for allergic bronchospasm; blood dyscrasias or convulsions that do not result in hospitalization; or development of IP dependency or IP abuse.

An AE is considered “life-threatening” if, in the view of either the investigator or sponsor, its occurrence places the participant at immediate risk of death. It does not include an AE that, had it occurred in a more severe form, might have caused death.

Adverse drug reaction (ADR) is any noxious and unintended response to a medicinal product related to any dose of the product. In accordance with International Council for Harmonisation of Technical Requirements for Pharmaceuticals for Human Use (ICH) guideline-E2A, the definition of an ADR implies a reasonable possibility of a causal relationship between the AE and the IP.

An **unexpected ADR** is an AR, the nature and severity of which is not consistent with the applicable reference document (reference safety information in the investigator’s brochure).

Reports which add significant information on specificity or severity of a known, already documented AE constitute unexpected AEs. Specific examples would be: acute renal failure as a labeled AE with a subsequent new report of interstitial nephritis or hepatitis with a subsequent report of fulminant hepatitis.

A **suspected unexpected serious adverse reaction (SUSAR)** is a suspected AR related to the IP that is both unexpected and serious. SUSARs will be subject to expedited regulatory reporting.

14.1.2 Adverse events of special interest (AESI)

AESIs represent any events for which additional data (besides the standard AE data) are desired. These may be at the request of the regulatory agency, industry partner, or driven by a regulatory requirement, or by a known or potential risk from the product or class. Non-structured data similar to SAEs will be collected for AESIs. AESIs encompass:

- Any event listed under holding rule in Section 11.1.6
- any new ICD diagnosis (per current International Statistical Classification of Diseases and Related Health Problems) that is applied to the participant during the course of the study after receipt of the IP and that is expected to continue for at least 3 months and requires continued health care intervention

- defined as hospitalization, an emergency room visit, or any otherwise unscheduled visit to or from medical personnel for any reason in connection with
  - suspected immune-mediated conditions (potential immune-mediated disease, see Appendix 2), or
  - AEs relevant to SARS-CoV-2 vaccine development or the target disease (based on Brighton Collaboration via CEPI's Safety Platform for Emergency vACcines (SPEAC) Project; see Appendix 3)

AESIs should be reported to the sponsor within 24 hours of awareness and documented in the eCRF AESI page. In case of a serious AESI, the SAE Form must also be completed.

14.1.3 Classification of adverse events

Causality

The causal relationship between the AE and the IP will be assessed as follows:

- Related: There is a reasonable possibility of a causal relationship between the event and the IP. This means that there are facts (evidence) or arguments to suggest a causal relationship.
- Not related: There is no reasonable possibility of a causal relationship between the event and the IP. This means that there are neither facts (evidence) nor arguments to suggest a causal relationship.

Severity

All AEs and SAEs will be assessed for severity, according to Table 3.

Table 3: Severity grading of adverse events

Grade	Intensity	Description
1	Mild	Transient symptoms, no interference with the participant's daily activities
2	Moderate	Some interference with activity not requiring medical intervention
3	Severe	Prevents daily activity and requires medical intervention
4	Life-threatening	Life-threatening consequences; urgent intervention indicated. Emergency room visit or hospitalization.

Outcome categories

- Resolved: The participant has fully recovered from the event or the condition has returned to the level observed at Baseline
- Resolving: The participant has recovered from the event, but the condition has not returned to the level observed at Baseline
- Not resolved: The event is ongoing at the time of reporting and the condition has not returned to the level observed at Baseline
- Resolved with sequelae: As a result of the AE, the participant suffered persistent and significant disability or incapacity (e.g., became blind, deaf, or paralyzed) or the condition became chronic or stabilized and no worsening is expected
- Fatal: The participant died due to the event. If the participant died due to other circumstances than the event, the outcome should be stated otherwise (not resolved or resolving)
- Unknown: If the outcome is not known or not reported

Action taken with IP

Action taken with the IP will be assigned to one of the following categories:

- Administration delayed: administration of the IP must be delayed
- Drug withdrawn: discontinuation of the IP
- Not applicable: this category should be used in circumstances such as when the participant has died, or the treatment had been completed prior to reaction(s) or event(s) or the IP had not been administered

14.1.4 Documentation of adverse events

All AEs that occur after the participant has signed the ICF until the participant's study completion (EoS) must be recorded. The occurrence of unsolicited AEs should generally be sought by non-directive questioning of the participant at each visit during the study, unless required otherwise, e.g. "Have you experienced any problems since your last contact?" AEs may also be detected when they are volunteered by the participant during or between visits or through physical examination, laboratory test, or other assessments.

The AE term should be reported in standard medical terminology when possible. AEs with verbatims matching with the terms listed in the FDA Center for Biologics Evaluation and

Research (CBER) guidelines on toxicity grading scales will be graded according to the specific definitions of severity provided in Appendix 1. Unsolicited AEs will be graded on a 4-point scale (see Section 14.1.3).

All unsolicited AEs (and solicited AEs that are ongoing at Day 8 after Prime-2-CoV_Beta booster vaccination), which occur during the study, will be recorded in the participant's AE section of the eCRF and will include the following information: event term, date of onset and resolution, severity, intensity, relationship to IP, action taken with IP, and outcome. For SAEs, the SAE Form must also be completed.

If possible, a diagnosis should preferably be entered as the AE term rather than signs or symptoms of an event. If a diagnosis changes over time, the event term should be modified accordingly.

Changes in severity or frequency: A continuous event changing in severity will be considered 1 event with the most severe intensity documented.

Intermittent AEs will only be recorded once, as long as the severity does not change and 'intermittent' is added to the verbatim text of the AE term. If, however, the AE changes from intermittent to continual, the original event should be closed out and reopened as a new AE.

Changes in seriousness: A continuous event with a changing seriousness will be considered 1 serious event.

14.1.5 Reporting of serious adverse events and suspected unexpected serious adverse reactions

The investigator must record all SAEs on the AE page form within the eCRF within 24 hours of awareness of the event. Upon submission of this form by the investigator, the eCRF system automatically releases a notification of the initial report to the drug safety department of the sponsor's designee.

If, due to any reason, the electronic completion and forwarding of the SAE form via the eCRF is not possible, the investigator must complete a paper SAE report and send it within the same timeframe to the safety department of the sponsor's designee by efax or e-mail ([redacted]**@fgk-cro.com** or **+49**[redacted]), the efax or email will automatically forward the report to both parties.

The initial report should contain as much information as possible, but at least the following information:

- Participant identification
- IP information (date of administration)
- Event term (only one term should be entered)

- Date and time of onset

  An actual time point or best estimate (hh:dd:mm:yy) of onset of the event should be given. If the hour of onset is not available, day, month and year are acceptable. If the day of onset is not available, month and year are acceptable. If the month of onset is not available, year is acceptable.

- Name of the investigator
- Causality assessment (relationship to IP)
- Severity

All SAEs must be followed up by the investigator until the event has resolved, resolved with sequelae, or until the participant died, whichever occurs first. The investigator should complete missing or requested information and submit follow-up reports, as appropriate. Some events do not have an “end”, such as metastasis; however, once these events are determined by the investigator to be stable or chronic, the investigator may consider the event to be resolved with sequelae.

Although post-study events are not required to be routinely sought or collected by the sponsor, SAEs that occur after a participant has completed a clinical study (including any protocol required post-treatment follow-up) must be reported by the investigator to the sponsor. Such cases should be regarded for expedited reporting purposes as though they were study reports. Therefore, a causality assessment and determination of expectedness are needed for a decision if expedited reporting is required.

SUSARs will be expedited reported to independent ethics committees (IECs), competent authority (CA) and investigators following pertinent national legislation. In any case, fatal and life-threatening SUSARs will be reported within 7 calendar days timeline after becoming aware of the reaction and any further relevant information will be reported within 8 calendar days of the initial report.

All other SUSARs should be reported within 15 calendar days.

Moreover, all SUSARs for the relevant IMP for all studies with the same sponsor should be reported to the principal investigators, regardless whether the event occurred in the present study or not.

Annual safety reporting to regulatory authorities and the IECs will follow pertinent national legislation.

14.1.6 Follow-up of adverse events

If an AE is still ongoing at the participant’s study completion visit (EoS), the AE documentation period for this participant will be prolonged until the AE is resolved or until the investigator

assesses the AE as stabilized (resolved with sequelae), and all AE-related queries were resolved, or the investigator considers the event as not medically concerning (resolving).

The investigator will take all appropriate and necessary therapeutic measures required for resolution of the AE, if applicable. All efforts to collect follow-up information must be documented in the source data.

14.2 Pregnancy

Any pregnancy that occurs during the study participation in any female participant or any female partner of a male participant must be recorded on the appropriate pregnancy form and reported within 24 hours of the first awareness of the event to the safety department of the sponsor's designee by efax or email ([redacted]**@fgk-cro.com** or **+49**[redacted]).

If pregnancy occurs participants should stay in the study. Pregnancy is not regarded an AE unless there is a suspicion that the IP may have interfered with the effectiveness of a contraceptive method.

The outcome of all pregnancies (spontaneous miscarriage, elective termination, normal birth, or congenital abnormality) must be followed up and documented. All reports of congenital abnormalities or birth defects are SAEs. Spontaneous miscarriages should also be reported and handled as SAEs. Elective abortions without complications should not be handled as AEs.

Pregnancy follow-up will be recorded on the pregnancy outcome form and will include an assessment of the possible relationship of the IP to any pregnancy outcome. The pregnancy outcome form should be reported within 4 weeks after delivery to the safety department of sponsor's designee by efax or email ([redacted]**@fgk-cro.com or +49**[redacted]).

14.3 Medication errors

Medication errors may result from the administration of the IP to the wrong participant, or at the wrong time, or at the wrong dose strength. Examples for medication errors include administration of expired IP, administration of IP that has undergone temperature excursion from the specified storage range, use of IP outside of what is foreseen in the protocol, administration of IP to subjects not involved in the study or foreseen to receive another IP.

Medication errors should be recorded in the eCRF as protocol deviation.

If a medication error is accompanied by an AE, as determined by the investigator, the AE should be recorded on the AE page of the eCRF.

14.4 Diary

Participants will be required to complete a web-based e-diary using the participant's own personal device. The participant will be asked to daily monitor and record local and systemic solicited events including start and stop day, and whether medication was taken to relieve the symptoms for 7 days after Prime-2-CoV_Beta booster vaccination. The diary is an integrated part of the eCRF and diary data are, thus, immediately available for review by the investigator. Each participant receives a user account to be able to use the e-diary application. The account is automatically generated in the eCRF when the investigator initiates the e-diary for the participant.

Participants will be instructed on how to complete the diary and how to self-assess the severity of solicited events. The participant will assess the event as absent, mild, moderate, or severe based on the FDA CBER guidelines on toxicity grading scales Appendix 1.

The following solicited events will be collected:

- Local events: pain at injection site, swelling, induration, and redness.
- Systemic events: fever (measured orally), fatigue, headache, chills, vomiting, nausea, diarrhea, new or worsened muscle pain, new or worsened joint pain

Redness, swelling and induration will be assessed with a measuring tape and then categorized according to Appendix 1.

For solicited AEs, the investigator will assess the relationship between study vaccine and occurrence of each AE.

In case of a solicited event of at least Grade 3, participants are asked to contact the center for further evaluation that may include an unscheduled clinic visit. However, in case of an emergency, the participant should call first an emergency physician, if needed.

If a participant experiences a confirmed Grade 4 local or systemic event, the investigator must immediately notify the sponsor. Only an investigator or medically qualified person is able to classify a participant's local or systemic reaction as Grade 4. Solicited Grade 4 events must be documented on the AE and SAE page of the eCRF.

Study personnel will review the diary with the participants at 1 day and 2 days (telephone), and 3 and 7 days (at center) after Prime-2-CoV_Beta booster vaccination. If any of the solicited events are ongoing 7 days after the vaccination day, they must be recorded as an AE in the eCRF with the same start date as indicated on the diary and followed up as described in Section 14.1.6. Otherwise solicited events will not be recorded as AEs in the eCRF.

14.5 Clinical safety laboratory investigations

All laboratory samples must be handled and labeled according to the respective laboratory manuals.

Biological samples for clinical safety laboratory tests will be collected as indicated in Table 1. Analyses will be done at the center's local laboratory. All safety laboratory assessments and parameters are specified in Table 4.

A blood sample for pregnancy testing will be taken from women of childbearing potential on Screening and a urine sample on Day 1 prior to study vaccination to establish eligibility.

The results of hematology, biochemistry, coagulation, and urinalysis must be available and assessed by the investigator before vaccination to confirm the eligibility of the participant.

The laboratory reports received from the laboratory will be reviewed, signed, and dated by the investigator, and filed at the center.

Clinically significant abnormal laboratory findings as judged by the investigator should be recorded as AE in the eCRF in accordance with the FDA toxicity grading scale (Appendix 1, A-Table 2).

In case of cardiac biomarkers abnormalities further diagnostic work-up including ECG and/or more accurate and sensitive methods for cardiovascular disease diagnosis must be performed.

Table 4: Clinical safety laboratory parameters

Laboratory assessment	Parameters
Hematology	Platelets, platelet distribution width, mean platelet volume, red blood cell counts, red blood cell distribution width, leukocytes, white blood cell counts with differentials and absolute counts (neutrophils, lymphocytes, monocytes, eosinophils, basophils, immature granulocytes), normoblasts (differential and absolute), hemoglobin, hematocrit, mean corpuscular volume, mean corpuscular hemoglobin, mean corpuscular hemoglobin concentration, reticulocytes (differential and absolute), reticulocyte hemoglobin equivalent, reticulocyte production index
Biochemistry	• Liver: alanine aminotransferase, aspartate aminotransferase, gamma-glutamyl transferase, alkaline phosphatase, total bilirubin<br>• Kidney: urea, creatinine, estimated glomerular filtration rate (CKD-EPI and/or MDRD[a])<br>• Heart: troponin, creatine kinase MB<br>• Other: potassium, sodium, magnesium, calcium, lactate dehydrogenase, total protein, albumin, high-sensitive C-reactive protein
Coagulation	Prothrombin time (international normalized ratio), partial thromboplastin time, fibrinogen
Urinalysis (performed only at Screening)	pH, glucose, protein, blood, ketones, nitrite, leukocytes by dipstick<br>microscopic examination (only if blood, protein, nitrite, or leukocytes are positive on the dipstick)
Serology	Hepatitis B core antibody, hepatitis B surface antigen, hepatitis C antibody, human immunodeficiency virus
Pregnancy test urine and blood:	Human chorionic gonadotropin (only for women of childbearing potential)

[a] Estimated glomerular filtration rate will be calculated according to the Chronic Kidney Disease Epidemiology Collaboration (CKD EPI) and/or Modification of Diet in Renal Disease (MDRD) equation.

14.6 Vital signs and physical examination

Vital signs and physical examinations will be performed at time points as specified in the schedule of assessments (Table 1).

Vital signs

Vital signs, including oral temperature, pulse rate and diastolic and systolic blood pressure (after participant was seated for at least 5 minutes) should be measured before blood is drawn for laboratory tests. Clinically significant abnormal findings in vital signs as judged by the investigator must be recorded as AE in the eCRF in accordance with the FDA toxicity grading scale (Appendix 1, A-Table 3).

At Screening, height, and weight (to calculate the body mass index) will also be assessed.

Physical examination

A physical examination will be performed evaluating any clinically significant abnormalities within the following body systems: general appearance; skin; head, eyes, ears, nose, and throat; heart; lungs; abdomen; musculoskeletal; extremities; neurological; and lymph nodes.

A targeted or symptom-directed physical examination will be performed at the time points specified in the schedule of assessments (Table 1).

14.7 Cardiac safety monitoring

Cardiac risk monitoring has been established based on cardiac histopathological findings observed after repeated vaccinations in rabbits (Section 8.4).

Laboratory

Serum cardiac markers i.e., troponin and CK-MB, will be assessed at all time points when safety laboratory will be assessed as specified in (Table 1).

ECG

ECGs will be performed at time points as specified in the schedule of assessments (Table 1). In addition, ECGs must be performed whenever there are abnormalities in troponin and/or CK-MB laboratory values and/or whenever clinical symptoms suggestive of cardiac injury occur.

ECGs will be recorded before blood is drawn for laboratory tests, if applicable, and after the participant has been in supine position for at least 5 minutes. The ECG results will be reviewed by a cardiologist or qualified medical specialist.

Echocardiography

A transthoracic echocardiography will be performed at time points specified in the schedule of assessments (Table 1). The echocardiography will be performed and evaluated (clinically significant finding yes or no) locally according to local clinical practice by an experienced cardiologist.

Other procedures

In addition to ECGs, the participant should be referred to additional sensitive diagnostic measures, e.g., cardiac MRI in case of troponin and/or CK-MB laboratory abnormalities and/or whenever clinical symptoms suggestive of cardiac injury occur.

Clinically significant abnormal findings in vital signs as judged by the investigator must be recorded as AE in the eCRF. Investigators should discuss any findings of concern or possible cardiac events with the medical monitor.

Participants experiencing clinical symptoms suggestive of cardiac injury and/or cardiac biomarker or ECG abnormalities should be referred to a specialist and/or, preferentially,

admitted to hospital for cardiac monitoring and further medical management, and the sponsor should be informed.

14.8 Safety monitoring

Safety oversight will be conducted by an SRC including several medical experts.

The SRC will review safety data after all participants of a dose cohort have completed Day 8, ad hoc in case holding rules (Section 11.1.6) apply. Further ad hoc SRC meetings may be scheduled upon request by the sponsor or principal investigators. After such safety reviews the SRC will make recommendations to the sponsor whether to continue, modify, or stop the study (see also Section 11.1.5).

Procedures for SRC reviews and meetings will be described in a charter that will be approved by the sponsor.

15 Biostatistical methods

Statistical methods are briefly described below and will be further detailed in a statistical analysis plan, which will be finalized before vaccination of the first participant.

15.1 Sample size calculation

The sample size is based on clinical and practical considerations and not on statistical hypothesis testing. The sample size is considered sufficient to evaluate the objectives of the study.

15.2 General approach

For qualitative variables, the frequencies (absolute and relative) will be calculated. Quantitative parameters will be described by declaring the mean value, standard deviation, minimum, first quartile, median, third quartile, and maximum. Analyses will be done by cohort and overall (except for immunogenicity endpoints).

Baseline values for, e.g., vital signs, safety and immunogenicity laboratory assessments, are defined as the last available assessments before Prime-2-CoV_Beta booster vaccination.

15.3 Handling of missing data

Statistical analyses will be done using observed values only. No imputation of missing values (e.g., no last observation carried forward) is planned, unless stated otherwise.

15.4 Analysis sets and types of analyses

Safety analysis set (SAF)
The safety analysis set (SAF) will consist of all participants who received at least 1 dose of IP. If it is uncertain if the participant has received IP, the participant will be included in the SAF.

Immunogenicity set (IMS)
The immunogenicity set (IMS) will consist of all participants who received at least 1 dose of IP and have at least 1 post baseline immunogenicity assessment.

Immunogenicity per-protocol set
The immunogenicity per protocol set (IM-PPS) will consist of all participants included in the IMS, who have no major protocol deviations.

Assignment of analysis sets to analyses and allocation of participants

All analyses will be based on the SAF, except for immune response endpoints that will be analyzed for the IMS and the IM-PPS.

Participants will be allocated to analysis sets during a data review meeting before data base lock.

15.5 Analysis of study conduct and participant disposition

The number of participants enrolled and receiving IP injection will be presented in detail to clearly describe the dose pattern used. The proportion of participants who prematurely discontinued the clinical study will be summarized together with the reason of discontinuation.

15.6 Analysis of baseline characteristics

Demographic and baseline characteristics (e.g., age, sex, height, weight) as well as medical history, will be summarized by cohort using descriptive statistics (quantitative data) and contingency tables (qualitative data).

15.7 Analysis of the primary endpoints

The primary endpoint (see Section 7) is safety including the proportion of participants with

- serious and non-serious AEs
- local and systemic solicited AEs.

The analysis of these endpoints will be done descriptively and is described under safety in Section 15.11.

15.8 Analyses of secondary immunogenicity endpoints

Immunogenicity endpoints will be descriptively analyzed by cohort and visit. Separate analyses will be done for the different parameters, e.g., different antibodies evaluated with different serological immunogenicity assays.

Secondary immunogenicity endpoints based on RBD-specific antibodies and neutralizing antibodies

Titer values < lower limit of quantification (LLOQ) will be converted for antibody analyses to 0.5 × LLOQ, and values > upper limit of quantification (ULOQ) to ULOQ if not stated otherwise.

Absolute values of serological immunogenicity assays as described will be presented using descriptive summary statistics by cohort and visit. Additionally, GMT with 95% confidence interval (CI) will be provided. For each cohort, serological immunogenicity assays will be presented graphically displaying GMT with 95% CIs at all visits.

GMT and 2-sided 95% CI will be calculated by exponentiating the mean logarithm of the titers and the corresponding CIs (based on Student´s t distribution).

The fold change from Baseline will be computed for each participant. The fold rise is calculated as the ratio of the post- vs pre-vaccination titer value. The fold change from Baseline will be summarized using descriptive statistics by cohort and visit. Additionally, the GMFR with 95% CI will be presented.

When the pre-vaccination value is < LLOQ and the post value is at or above LLOQ, < LLOQ will be converted to LLOQ. When the pre-vaccination value is at or above LLOQ and post vaccination value is < LLOQ, < LLOQ will be converted to 0.5 × LLOQ. When both the pre and post values are < LLOQ, the fold rise is set to 1.

15.9 Further exploratory research analyses

Analyses based on neutralizing antibodies, and S- and N-specific antibodies will be performed as described above for RBD-specific antibodies. Additional exploratory immunogenicity analyses will be done descriptively and described elsewhere, as applicable.

15.10 Analyses of anti-vector immunity

ORFV-specific antibodies will be summarized descriptively as described above for virus-specific antibodies.

15.11 Safety analyses

All safety data will be summarized descriptively overall and by cohort.

Solicited AEs

Solicited local and systemic AEs within 7 days after Prime-2-CoV_Beta booster vaccination will be summarized by descriptive statistics using contingency tables (counts of events, number and proportion of participants with events). For participants with more than 1 episode of the same AE within 7 days after a vaccination, the maximum intensity will be used for analysis. Solicited local and systemic AEs will also be presented by intensity and solicited systemic AE and also by relationship to IP. Time to onset (in days) and duration (in days) will also be analyzed using descriptive summary statistics for each solicited local and systemic AE.

Unsolicited AEs

Unsolicited AEs will be coded according to MedDRA. Analyses will focus on TEAEs. Pre-treatment-emergent AEs will only be listed. The frequency (% of participants) of treatment-emergent SAEs, TEAEs, and AESIs throughout the study will be analyzed.

TEAEs will be summarized by descriptive statistics using contingency tables (counts of events, number and proportion of participants with events) and presented by system organ class and preferred term (PT), as well as by severity. Similar tables will be provided for serious TEAEs, related TEAEs, AESIs, related AESIs, TEAEs leading to death, and TEAEs resulting in discontinuation of the study. If the start or stop date/time of an AE is incomplete and the allocation to pre-treatment emergent or treatment emergent is not clear, the event will be considered treatment emergent. AESIs will be identified during a data review meeting prior to data base lock.

The frequency (% of participants) of treatment-emergent SAEs, TEAEs, and AESIs throughout the study will be tabulated.

Clinical laboratory

Laboratory variables will be assessed by the investigator as: 'normal', 'abnormal, not clinically significant (NCS)', or 'abnormal, clinically significant (CS)' in relation to the normal range given by the laboratory. The absolute values, and absolute and relative change from Baseline will be analyzed using summary statistics by parameter and visit. Urinalysis data will only be listed.

In addition, shift tables will be created for each laboratory value to display the change from Baseline to each post-baseline visit[2] in the number and proportion of participants with normal, abnormal-NCS, or abnormal-CS values.

---

[2] Refers only to post-baseline visits with safety laboratory or vital signs assessments, respectively.

The number and percentage of participants with clinically significantly abnormal values (as assessed by the investigator) during the study but without a clinically significantly abnormal value for the respective baseline assessment will be analyzed by laboratory parameter and by visit.

Physical examinations, ECG, echocardiography, vital signs and other safety measures

For vital signs and ECG assessments absolute values as well as the absolute and relative change from Baseline (last value before vaccination) will be analyzed by parameter and visit using summary statistics.

For ECG and vital sings: the number and proportion of participants with clinically significantly abnormal values (as assessed by the investigator) during the study but without a clinically significantly abnormal value for the respective baseline assessment will be analyzed by parameter and by visit.

Physical examination results will be analyzed using descriptive statistics. Additionally, shift tables will be produced to assess the changes from Baseline to respective post-booster visits.

Results of the echocardiography will be listed.

Medications will be coded according to the World Health Organization drug dictionary (WHO-DD). Previous medications will be listed. Concomitant medications will be presented by anatomic therapeutic chemical classification Level 1 and Level 4, and PT. The number of medications, as well as the number and proportion of participants taking the medication will be presented. If the start or stop date is incomplete and the allocation to previous or concomitant is not clear the medication will be considered concomitant.

15.12 Interim analysis

No formal interim statistical analysis is planned.

Safety data will be analyzed periodically for SRC review. An informal analysis of all collected data will be performed when all participants have completed Day 15 to determine the right dose group to move into an extension period and/or phase II. This analysis will focus on the primary endpoints and secondary endpoints evaluated at Day 15. Further endpoints and assessments may be evaluated as appropriate.

In case of any safety concerns, an additional safety analysis will be performed.

16 Participant withdrawal

Participants are free to withdraw from participation in the study at any time upon request, without giving reasons and without any consequence. The investigator may withdraw the participant at any time in the interests of the participant's health and well-being. In addition, the participant may withdraw or be withdrawn for any of the following reasons:

- Participant refuses further follow-up
- Lost to follow-up
- Death
- Participant request (withdrawal of consent)
- Investigator request
- Significant protocol deviation
- Non-compliance with study requirements
- AE, which requires discontinuation of the study involvement

The reason for withdrawal will be recorded in the eCRF. If withdrawal is due to an AE, the AE form of the eCRF must be completed, appropriate follow-up visits or medical care must be arranged, and the AE followed up until it is resolved or until the investigator assesses the AE as stabilized (resolved with sequelae), and all AE-related queries were resolved.

If a participant is withdrawn from the study for multiple reasons that include AE, the study completion eCRF page should indicate that the discontinuation was related to an AE.

If a participant does not return for a scheduled visit, every effort should be made to contact the participant to complete EoS assessments, or at least participant outcome, if possible. All attempts to contact the participant and information received during contact attempts must be documented in the participant's source document.

If the participant withdraws consent, no further study-related procedures must be performed, and no attempts should be made to collect additional data.

16.1 Replacement of participants

Participants who withdraw or are withdrawn from this study or are lost to follow-up after signing the informed consent but before Prime-2-CoV_Beta booster vaccination may be replaced. Participants who receive study vaccine and subsequently withdraw from the study will not be replaced.

17 Ethical and legal requirements

17.1 General requirements

The study will be performed in accordance with the ICH guideline for good clinical practice (GCP; CPMP/ICH/135/95), the appropriate national regulations and the Declaration of Helsinki in its currently acknowledged version.

The investigator and the sponsor must also comply with the General Data Protection Regulation [26].

17.2 Independent ethics committees

Before the initiation of the clinical study, the final protocol, any amendments if applicable, the participant information sheet and consent form, as well as any additional documents which are required by national regulations and the IEC will be submitted to the competent IEC for review. A favorable opinion for the clinical study must be obtained from the IEC before any participant is enrolled at the respective center.

If appropriate, any additional requirements imposed by the IEC will be followed. Amendments to the study documents will be notified to, or approved by, the IEC before implementation, if applicable.

17.3 Participant information and consent procedure

Before any clinical study-related activities are performed, the investigator (or authorized designee) must review the ICF and explain the study to potential study participants. The investigator must ensure that the participant is fully informed about the aims, procedures, potential risks, any discomforts, and expected benefits of the clinical study. Before consenting, the participant must be left with ample time to consider and ask questions. It must be emphasized that participation is voluntary, and that the participant has the right to withdraw from the clinical study at any time without prejudice and without disadvantages. The participant must then sign and date the consent form before the conduct of any study procedures. The investigator must sign and date the informed consent as well.

A copy of the participant information and ICF will be given to the participants for their records. The rights and welfare of the study participants will be protected by emphasizing to them that the quality of their medical care will not be adversely affected if they decline to participate in this clinical study.

If amendments to the final study protocol affect the participant’s participation in the clinical study (e.g., a change in any procedure), the participant information and ICF must be updated to

incorporate this modification, and participants must agree to sign the amended form indicating that they re-consent to participate in the clinical study.

Participants will be asked to provide consent for the use of blood samples for future testing to obtain further data on the immune response to Prime-CoV-2_Beta and to support further vaccine development. Aliquots of all collected samples from this study may be retained for additional testing for a maximum of 15 years (starting from the date at which the last participant had the last study visit), as long as this procedure is in line with applicable local rules, regulations, and guidelines.

17.4 Insurance coverage

Insurance coverage for damages emerging from the clinical study will be provided according to applicable legal requirements. During the informed consent procedure, the investigator must inform the participant accordingly. Insurance details will be provided to the participant within the participant information sheet.

17.5 Submission to authorities

Documents required for the study application will be submitted to the responsible CA. The study will not start until this authority has authorized the study. Amendments to the study protocol or to any other documents that must be reviewed by the CA will also be submitted to the CA in accordance with the regulatory requirements. If applicable, authorization of the amendment must be awaited before implementing any changes.

17.6 Participant confidentiality

The study protocol, documentation, data, and all other information generated will be held in strict confidence. No information concerning the clinical study or the data will be released to any unauthorized third party without prior written approval of the sponsor.

Personal participant data will be kept confidential in compliance with the EU General Data Protection Regulation [26] and other applicable international and national requirements.

The investigator must ensure that the pseudonymity of study participants will be maintained and that their identities are protected from unauthorized parties. On eCRFs, compensation documentation, or any other documents submitted to the sponsor’s representative or to any other parties outside the study center, participants must be identified only by their participant number; it is not allowed to use their names, addresses, telephone numbers, or similar information. The investigator will keep the original of the Participant Identification Log (including complete name and date of birth of each participant) in his/her file. The investigator must maintain these documents in strict confidence.

To allow compliance with GCP, all participants will be asked for consent regarding the access to their personal clinical study-related data for monitoring, audits, and inspections as well as regarding transmission and storage of their pseudonymous data; a respective statement will be part of the ICF. Professionals getting access to source data for monitoring, audits and inspections are bound to preserve strict confidentiality.

18 Criteria for premature termination of the study and criteria for initializing and closing a study center

18.1 Criteria for terminating the clinical study

The sponsor reserves the right to halt or terminate the study at any time. Reasons for termination include but are not limited to:

- Potential health risk for participants
- High withdrawal rate
- New scientific knowledge becomes available that makes the objectives of the trial no longer feasible or valid
- Insufficient enrolment of participant
- Request of the sponsor or regulatory authority, or IEC withdraws its approval

If the study is prematurely terminated or suspended, the sponsor will promptly notify the relevant CA, IEC, and the investigator(s) in writing about the termination of the study and its reasons.

18.2 Criteria for closing a study center

A study center may be closed for the following reasons:

- The center is unable to recruit enough participants within the agreed time frame
- The center does not respond to study management requests
- Repeated protocol or GCP violations

The investigator may terminate study participation prematurely. In this case, the investigator must notify the sponsor in writing stating the reasons for early termination. In terminating the study, the sponsor and the investigator will ensure that adequate measures are taken to protect the participants' interests.

The investigator will notify the relevant CA or IEC in writing if required, submit a copy of this notification to the sponsor and return all IP supplies and all related study material, as applicable, to the sponsor.

19 Study protocol, documentation and archiving of data

19.1 Amendments to the protocol

Any change to the protocol concerning e.g., the purpose of the study, the study design, or the participant's eligibility can only be made in the form of a written amendment to the study protocol. Protocol waivers are not allowed.

These amendments must be discussed and signed by all relevant parties before implementation.

Substantial amendments, i.e., amendments which are likely to affect to a significant degree

- the safety or physical or mental integrity of the study participants
- the scientific value of the study
- the conduct or management of the study, or
- the quality or safety of any IP used in the study

will be submitted to the CA and IEC for an authorization and favorable opinion as required by applicable regulations. If these amendments affect the participant's participation in the clinical study (e.g., a change in any procedure), the participant information sheet and the ICF must be updated to incorporate this modification, and currently enrolled participants must re-consent to study participation.

Non-substantial changes, e.g., minor corrections of administrative nature or rephrasing, which do not meet the above criteria for being substantial, are considered editorial changes. Such minor corrections will be reported to the IEC and CA together with the next substantial amendment.

If new events occur related to the conduct of the study or the development of the IP, which may affect the safety of the participants, the sponsor and the investigator will take appropriate safety measures to protect the participants against any immediate hazard. The sponsor will immediately inform the CA and IEC of the new events and the measures taken.

19.2 Protocol deviations

A protocol deviation is a failure to follow, intentionally or unintentionally, the requirements of the protocol. As required by national regulation or guidelines, requests for deviations and reports of deviations will be provided to the IEC if the deviation affects the participant's rights, safety and well-being, or the scientific integrity of the study.

Under emergency circumstances, deviations from the protocol may proceed without prior approval by the sponsor and favorable opinion of the IEC if the rights, safety, and well-being of participants need to be protected. Such deviations will be documented and reported to the sponsor and the IEC as soon as possible in accordance with national regulations.

All protocol deviations will be listed. Whether or not the participants concerned will be evaluable for analysis will be discussed in a data review meeting prior to the statistical analysis.

19.3 Data retention

The sponsor's designee will provide an Investigator Site File (ISF) to each center. The ISF will include essential documents as defined by the ICH GCP guideline and applicable local requirements.

The investigator will be responsible to update and maintain the ISF, which will be reviewed periodically by the monitor(s). These documents may be reviewed during an audit by the sponsor or an inspection by the CA or IEC.

All essential documents at the study center, the sponsor or the sponsor's designee should be retained until at least 2 years after the last approval of a marketing application in an ICH region, until there are no pending or contemplated marketing applications in an ICH region, and until at least 2 years have elapsed since the formal discontinuation of clinical development of the tested IP and at least 15 years, whichever period is longer. The final report will be kept for another 5 years after the tested IP has been taken from the market according to the legal stipulations.

The documents should, however, be archived for a longer period if required by the applicable regulatory authorities or if agreed with the sponsor. The sponsor is responsible for informing the investigators when these documents do no longer need to be retained.

The medical files of study participants must be retained in accordance with local legislation and in accordance with the maximum period permitted by the hospital, institution or private practice.

20 Data collection, monitoring and quality assurance

20.1 Data collection

All data generated after the participant has given informed consent must be recorded in the eCRF in a timely manner. The principal investigator is responsible to ensure accurate and proper completion of the eCRF.

Only investigators and their designees will enter and edit the data via a secure network and a secure access system. Completed data for each visit will be approved by the principal investigator or his/her deputy using an electronic signature to confirm the accuracy of the data on a regular basis. Any change or addition will be recorded by an electronic audit trail system.

A list of all persons who are allowed to make entries in the eCRF must be available in each study center.

The investigator must verify that all data entries in the eCRF are accurate and correct. Entries will be checked against appropriate source documentation by the monitor (Section 20.2).

20.2 Monitoring

The extent of monitoring and source data verification will be specified in a monitoring plan.

The study center may not obtain consent of any participant before the initiation visit. During the study further monitoring visits will be performed according to ICH GCP, the sponsor's designee's standard operating procedures, and local regulations. eCRFs will be reviewed against source data for adherence to the study protocol and ICH GCP, as well as for completeness, accuracy, and consistency of data. Additionally, the monitor will check the progress of enrolment, and will ensure that the IP is being stored, administered, and accounted for according to specifications. Key study personnel must be available to assist the monitor during these visits.

Risk-based monitoring including reduced source data verification, triggered monitoring, centralized monitoring, and targeted monitoring may be conducted [27-30]. The extent and details of this monitoring methods will be defined in the monitoring plan.

The investigator must allow the monitor access to the participant's medical records and all applicable source documents. Throughout the study, all data captured in the eCRF will only be identified by a participant number. The data will be pseudonymized correspondingly in all data analyses.

It is the investigator's obligation to assure documentation of all relevant data in the participant's file, such as medical history and concomitant diseases, date of written informed consent, visit dates, results of examinations, administrations of medication, and AEs.

Should physical monitoring visits at the center not be feasible or restricted for some time due to an epidemic situation, either a combined remote and on-site monitoring or full remote monitoring visit may be conducted during these periods. In line with local laws and regulations remote source data verification may be part of the remote monitoring visits and if so, must be agreed between the sponsor's designee and the study center. If necessary, the relevant study documents (e.g., monitoring plan) will be adjusted to reflect these activities.

Options for remote monitoring - in compliance with the local, country specific regulations, requirements and relevant guidelines - might be telephone calls, audio/videoconferencing, and sharing of medical records (e.g., direct access to Electronic Health Records [31,32] sharing redacted/blinded medical records in a secured way, etc.), depending on the center's capability to contribute.

The ICH-GCP requirements and applicable data protection and privacy regulations must be met in any case and for any selected monitoring approach. In case of a remote monitoring of data, the participants need to agree to it in the ICF.

20.3 Audits and inspections

During the study, audits may be performed by independent auditors, the sponsor, or representatives of the sponsor. Audits of clinical research activities will be performed in accordance with corresponding standard operating procedures to ensure compliance with the principles of GCP.

Regulatory authorities may wish to conduct an inspection. If an inspection is requested, the investigator must inform the sponsor or the sponsor's designee immediately.

The investigator must allow the persons performing the audits or inspections access to source data and documents and will answer any questions.

Inspections by CA and IEC are possible at any time, even after the end of study.

20.4 Data management procedures

All data management activities will be conducted by the sponsor's designee following their standard operating procedures. The database will be built by the sponsor's designee.

Details on data handling will be described in the data management plan. Data entered into the eCRF will be validated through online edit checks and offline checks run by the data manager or designee according to the data validation plan. All identified discrepancies will be queried and addressed to the investigator.

The sponsor's designee will handle the data cleaning process, query process, and coding.

For the final analysis, the database will be soft locked when the last participant has completed the last visit and when it is considered complete and accurate (i.e., all data cleaning activities performed). The database will be hard locked after all the changes following the data review meeting have been done and the database is considered complete and accurate. All changes will be tracked (audit trail). The sponsor's approval prior to database hard lock is mandatory.

The MedDRA will be used for coding of AEs and medical history. Concomitant medication will be coded using the WHO-DD **A**(natomical) **T**(heraputical) **C**(hemical) code.

During the study set-up, it is recommended to use the most recent version of WHO-DD and MedDRA dictionaries to ensure accurate and appropriate medical coding terminologies are referred for medical monitoring as well as safety review. Should there be any newer or upgraded dictionary versions released during the study, it will be under the sponsor's discretion to decide whether an upgraded dictionary version is needed for the rest of the study conduct.

20.5 Training of center staff

The investigator will ensure that everyone assisting with the clinical study is adequately informed about the protocol, the IPs and their study-related duties and functions. Furthermore, the investigator will maintain a list of qualified persons to whom the investigator has delegated study-related duties.

21 Study report and publications

After completion of the study, the results will be summarized in a clinical study report according to the ICH E3 Note for guidance on structure and content of clinical study reports. A summary report will also be prepared for the informal data analysis done after all participants have completed Day 15.

The sponsor or sponsor’s designee will send a summary of this clinical study report to the IEC and CA within the timeframes defined per national regulation or by the IEC.

By signing the study protocol, the investigator agrees with the use of results of the study for the purposes of national and international registration, publication, and information for medical and pharmaceutical professionals. If necessary, the authorities will be notified of the investigator’s name, address, qualifications, and extend of involvement.

An investigator shall not publish any data (poster, abstract, paper, etc.) without having consulted with the sponsor in advance. The sponsor maintains the right to be informed of any plans for publication and to review any resulting works, including abstracts, presentations, or manuscripts, before they are submitted. The sponsor will return its comments to the author in a timely manner.

The preparation and submission of abstracts or manuscripts including the study results must be in line with the process specified in the investigator’s clinical study agreement. The publication or presentation of any study results shall comply with all applicable privacy laws.

The study will be registered before the inclusion of the first participant in a public data base according to local regulations.

22 Study periods

Planned study start (first participant in):	May-2022
Planned study end (last participant out):	Jan-2023
Estimated recruitment period:	4 months

The end of the study is defined as last participant last visit.

23 References

1. CDC. COVID-19 situation update worldwide.; Published 2021. Updated 12 August 2021. Available at: https://www.ecdc.europa.eu/en/geographical-distribution-2019-ncov-cases.
2. Government UK. Guidance COVID-19: epidemiology, virology, and clinical features. Updated 18 February 2021. Available at: https://www.gov.uk/government/publications/wuhan-novel-coronavirus-background-information/wuhan-novel-coronavirus-epidemiology-virology-and-clinical-features.
3. McIntosh K. Coronavirus disease 2019 (COVID-19): Clinical features. UpToDate. Updated 20 November 2020. Available at: https://www.uptodate.com/contents/coronavirus-disease-2019-covid-19-clinical-features?topicRef=126981&source=see_link.
4. Amann R, Rohde J, Wulle U, Conlee D, Raue R, Martinon O, et al. A new rabies vaccine based on a recombinant ORF virus (parapoxvirus) expressing the rabies virus glycoprotein. J Virol 2013;87(3):1618-30.
5. Dory D, Fischer T, Béven V, Cariolet R, Rziha HJ, Jestin A. Prime-boost immunization using DNA vaccine and recombinant Orf virus protects pigs against Pseudorabies virus (Herpes suid 1). Vaccine 2006;24(37-39):6256-63.
6. Fischer T, Planz O, Stitz L, Rziha HJ. Novel recombinant parapoxvirus vectors induce protective humoral and cellular immunity against lethal herpesvirus challenge infection in mice. J Virol 2003;77(17):9312-23.
7. Henkel M, Planz O, Fischer T, Stitz L, Rziha HJ. Prevention of virus persistence and protection against immunopathology after Borna disease virus infection of the brain by a novel Orf virus recombinant. J Virol 2005;79(1):314-25.
8. Rohde J, Schirrmeier H, Granzow H, Rziha HJ. A new recombinant Orf virus (ORFV, Parapoxvirus) protects rabbits against lethal infection with rabbit hemorrhagic disease virus (RHDV). Vaccine 2011;29(49):9256-64.
9. Ahmed SF, Quadeer AA, McKay MR. Preliminary Identification of Potential Vaccine Targets for the COVID-19 Coronavirus (SARS-CoV-2) Based on SARS-CoV Immunological Studies. Viruses 2020;12(3).
10. Dhama K, Sharun K, Tiwari R, Dadar M, Malik YS, Singh KP, et al. COVID-19, an emerging coronavirus infection: advances and prospects in designing and developing vaccines, immunotherapeutics, and therapeutics. Hum Vaccin Immunother 2020;16(6):1232-8.
11. Tay MZ, Poh CM, Rénia L, MacAry PA, Ng LFP. The trinity of COVID-19: immunity, inflammation and intervention. Nat Rev Immunol 2020;20(6):363-74.
12. Yu J, Tostanoski LH, Peter L, Mercado NB, McMahan K, Mahrokhian SH, et al. DNA vaccine protection against SARS-CoV-2 in rhesus macaques. Science 2020;369(6505):806-11.
13. Quinlan BD, Mou H, Zhang L, Guo Y, He W, Ojha A, et al. The SARS-CoV-2 receptor-binding domain elicits a potent neutralizing response without antibody-dependent enhancement. bioRxiv 2020:2020.04.10.036418.
14. Wrapp D, Wang N, Corbett KS, Goldsmith JA, Hsieh CL, Abiona O, et al. Cryo-EM structure of the 2019-nCoV spike in the prefusion conformation. Science 2020;367(6483):1260-3.

15. Ahlén G, Frelin L, Nikouyan N, Weber F, Höglund U, Larsson O, et al. The SARS-CoV-2 N Protein Is a Good Component in a Vaccine. J Virol 2020;94(18).
16. Dutta NK, Mazumdar K, Gordy JT. The Nucleocapsid Protein of SARS-CoV-2: a Target for Vaccine Development. J Virol 2020;94(13).
17. EMA. Guideline on clinical evaluation of vaccines (draft) EMEA/CHMP/VWP/164653 rev.1 April 2018.[Online]. Available at https://www.ema.europa.eu/en/documents/scientific-guideline/draft-guideline-clinical-evaluation-vaccines-revision-1_en.pdf.
18. EMA. Guideline on strategies to identify and mitigate risks for first-in-human and early clinical trials with investigational medicinal products. EMEA/CHMP/SWP/28367/07 Rev. 1. 2017;[Online]. Available at: https://www.ema.europa.eu/en/documents/scientific-guideline/guideline-strategies-identify-mitigate-risks-first-human-early-clinical-trials-investigational_en.pdf.
19. Monath TP, Fast PE, Modjarrad K, Clarke DK, Martin BK, Fusco J, et al. rVSVΔG-ZEBOV-GP (also designated V920) recombinant vesicular stomatitis virus pseudotyped with Ebola Zaire Glycoprotein: Standardized template with key considerations for a risk/benefit assessment. Vaccine X 2019;1:100009.
20. Hervé C, Laupèze B, Del Giudice G, Didierlaurent AM, Tavares Da Silva F. The how's and what's of vaccine reactogenicity. NPJ Vaccines 2019;4:39.
21. Mathioudakis AG, Ghrew M, Ustianowski A, Ahmad S, Borrow R, Papavasileiou LP, et al. Self-Reported Real-World Safety and Reactogenicity of COVID-19 Vaccines: A Vaccine Recipient Survey. Life (Basel) 2021;11(3).
22. Su JR, Moro PL, Ng CS, Lewis PW, Said MA, Cano MV. Anaphylaxis after vaccination reported to the Vaccine Adverse Event Reporting System, 1990-2016. J Allergy Clin Immunol 2019;143(4):1465-73.
23. Sellaturay P, Nasser S, Islam S, Gurugama P, Ewan PW. Polyethylene glycol (PEG) is a cause of anaphylaxis to the Pfizer/BioNTech mRNA COVID-19 vaccine. Clin Exp Allergy 2021.
24. ACIP. Preventing and Managing Adverse Reactions. General Best Practice Guidelines for Immunization: Best Practices Guidance of the Advisory Committee on Immunization Practices (ACIP). Available at: https://www.cdc.gov/vaccines/hcp/acip-recs/general-recs/adverse-reactions.html.
25. Lee WS, Wheatley AK, Kent SJ, DeKosky BJ. Antibody-dependent enhancement and SARS-CoV-2 vaccines and therapies. Nat Microbiol 2020;5(10):1185-91.
26. European Parliament. Regulation (EU) 2016/679 of the European Parliament and of the Council of 27 April 2016 on the protection of natural persons with regard to the processing of personal data and on the free movement of such data, and repealing Directive 95/46/EC (General Data Protection Regulation). [Online]. Available from. http://data.europa.eu/eli/reg/2016/679/oj. .
27. EMA. Guidance on the Management of Clinical Trials during the COVID-19 (Coronavirus) pandemic. Version 4. 2021;[Online]. Available at: https://ec.europa.eu/health/sites/health/files/files/eudralex/vol-10/guidanceclinicaltrials_covid19_en.pdf.
28. EMA. Reflection paper on risk based quality management in clinical trials (EMA/269011/2013). 2013.
29. FDA. Guidance on Conduct of Clinical Trials of Medical Products during COVID-19 Pandemic, Guidance for Industry, Investigators, and Institutional Review Boards.

30. FDA. Guidance for Industry - Oversight of Clinical Investigations - A Risk-Based Approach to Monitoring. Available at: https://www.fda.gov/media/116754/download.
31. EMA. Reflection paper on expectations for electronic source data and data transcribed to electronic data collection tools in clinical trials, 09 June 2010, EMA/INS/GCP/454280/2010. Available at: https://www.ema.europa.eu/en/documents/regulatory-procedural-guideline/reflection-paper-expectations-electronic-source-data-data-transcribed-electronic-data-collection_en.pdf.
32. FDA. Guidance for Industry, Use of Electronic Health Record Data in Clinical Investigations. Available at: https://www.fda.gov/media/97567/download.
33. FDA. Guidance for Industry: Toxicity Grading Scale for Healthy Adult and Adolescent Volunteers Enrolled in Preventive Vaccine Clinical Trials. Published 2007. Available at: https://www.fda.gov/media/73679/download.

24 Approval and signatures

Protocol agreed to by sponsor:

Sponsor’s signatory name (print)

Sponsor’s signatory signature | Date

Protocol agreed to by statistician:

Statistician name (print)

Statistician signature | Date

Protocol agreed to by chief investigator:

Chief investigator Name (print)

Coordinating investigator signature | Date

Principal Investigator Agreement Page for the protocol

I agree:

- To assume responsibility for the proper conduct of the clinical study at this site, and to conduct the study in compliance with national law, the valid version of the Declaration of Helsinki, the ICH guideline for GCP (CPMP/ICH/135/95), the present study protocol including its amendments, and with any other study conduct procedures provided by the sponsor or authorized representatives.
- Not to implement any deviations from or changes to the protocol (including protocol amendments) without agreement from the sponsor and prior review and favorable opinion from the Ethics Committee and approval from the Competent Authority, if applicable, except where necessary to eliminate an immediate hazard to the participant(s), or for administrative aspects of the clinical study (where permitted by all applicable regulatory requirements).
- That I am familiar with the appropriate use of the investigational product as described in this protocol and any other information provided by the sponsor including, but not limited to, the current Investigator's Brochure or equivalent document provided by the sponsor.
- To ensure that all persons assisting me with the clinical study are adequately informed about the investigational product and of their study-related duties and functions.
- That I have been informed that certain regulatory authorities require the sponsor to obtain and supply details about the investigator's ownership interest in the sponsor or the study product, and more generally about his/her financial ties with the sponsor. The sponsor will use and disclose the information solely for the purpose of complying with regulatory requirements.

____
Principal investigator name (print)

____________________________________ ________________
Principal investigator signature Date

25 Appendices

Appendix 1 Grading scales

A-Table 1: Local and systemic reaction grading scale

	**Mild (Grade 1)**	**Moderate (Grade 2)**	**Severe (Grade 3)**	**Potentially life threatening (Grade 4)**
**Local**				
Pain	Does not interfere with activity	Repeated use of nonnarcotic pain reliever >24 hours or interferes with activity	Any use of narcotic pain reliever or prevents daily activity	Emergency room (ER) visit or hospitalization
Erythema/ Redness	2.5 - 5 cm	5.1 - 10 cm	> 10 cm	Necrosis or exfoliative dermatitis
Induration/ Swelling[a]	2.5 - 5 cm and does not interfere with activity	5.1 - 10 cm or interferes with activity	> 10 cm or prevents daily activity	Necrosis
**Systemic**				
Fever[b]	38.0 - 38.4 °C	38.5 - 38.9 °C	39.0 - 40.0 °C	> 40.0 °C
Nausea/ vomiting	No interference with activity or 1 - 2 episodes/24 hours	Some interference with activity or >2 episodes/24 hours	Prevents daily activity, requires outpatient IV hydration	ER visit or hospitalization for hypotensive shock
Diarrhea	2 - 3 loose stools in 24 hours	4 - 5 loose stools in 24 hours	6 or more loose stools in 24 hours or requires outpatient IV hydration	ER visit or hospitalization
Headache	No interference with activity	Repeated use of non-narcotic pain reliever >24 hours or some interference with activity	Significant; any use of narcotic pain reliever or prevents daily activity	ER visit or hospitalization
Fatigue	No interference with activity	Some interference with activity	Significant; prevents daily activity	ER visit or hospitalization
Chills	No interference with activity	Some interference with activity	Significant; prevents daily activity	ER visit or hospitalization
Muscle pain	No interference with activity	Some interference with activity	Significant; prevents daily activity	ER visit or hospitalization
Joint pain	No interference with activity	Some interference with activity	Significant; prevents daily activity	ER visit or hospitalization

[a] Induration/swelling should be evaluated and graded using the functional scale as well as the actual measurement.

[b] Oral temperature; no recent hot or cold beverages or smoking.

From [33] (slightly adapted).

A-Table 2: Laboratory abnormal grading scales

Hematology *	Mild (Grade 1)	Moderate (Grade 2)	Severe (Grade 3)	Potentially life threatening (Grade 4)
Hemoglobin (Female) - g/dL	11.0 - 12.0	9.5 - 10.9	8.0 - 9.4	< 8.0
Hemoglobin (Female), change from baseline value - gm/dL	Any decrease - 1.5	1.6 - 2.0	2.1 - 5.0	> 5.0
Hemoglobin (Male) - g/dL	12.5 - 13.5	10.5 - 12.4	8.5 - 10.4	< 8.5
Hemoglobin (Male), change from baseline value - g/dL	Any decrease – 1.5	1.6 - 2.0	2.1 - 5.0	> 5.0
WBC Increase - cell/mm^3	10,800 - 15,000	15,001 - 20,000	20,001 - 25, 000	> 25,000
WBC Decr. - cell/mm^3	2,500 - 3,500	1,500 - 2,499	1,000 - 1,499	< 1,000
Lymphocytes Decr. - cell/mm^3	750 - 1,000	500 - 749	250 - 499	< 250
Neutrophils Decr. - cell/mm^3	1,500 - 2,000	1,000 - 1,499	500 - 999	< 500
Eosinophils - cell/mm^3	650 - 1500	1501 - 5000	> 5000	Hypereosinophilic
Platelets Decr. - cell/mm^3	125,000 - 140,000	100,000 - 124,000	25,000 - 99,000	< 25,000
PT - increase by factor	1.0 - 1.10 x ULN	1.11 - 1.20 x ULN	1.21 - 1.25 x ULN	> 1.25 ULN
PTT - increase by factor	1.0 - 1.2 x ULN	1.21 - 1.4 x ULN	1.41 - 1.5 x ULN	> 1.5 x ULN
Fibrinogen increase - mg/dL	400 - 500	501 - 600	> 600	--
Fibrinogen decrease - mg/dL	150 - 200	125 - 149	100 - 124	< 100 or associated with gross bleeding or disseminated intravascular coagulation (DIC)
Other values (as defined according to applicable regulations)	No interference with activity	Some interference with activity not requiring medical intervention	Prevents daily activity and requires medical intervention	ER visit or hospitalization

A-Table 2: Laboratory abnormal grading scales *(continued)*

**Serum ***	**Mild (Grade 1)**	**Moderate (Grade 2)**	**Severe (Grade 3)**	**Potentially Life Threatening (Grade 4)[a]**
Sodium - Hyponatremia mEq/L	132 - 134	130 - 131	125 - 129	< 125
Sodium - Hypernatremia mEq/L	144 - 145	146 - 147	148 - 150	> 150
Potassium - Hyperkalemia mEq/L	5.1 - 5.2	5.3 - 5.4	5.5 - 5.6	> 5.6
Potassium - Hypokalemia mEq/L	3.5 - 3.6	3.3 - 3.4	3.1 - 3.2	< 3.1
Glucose - Hypoglycemia mg/dL	65 - 69	55 - 64	45 - 54	< 45
Glucose - Hyperglycemia<br>Fasting - mg/dL<br>Random - mg/dL	 100 - 110<br>110 - 125	 111 - 125<br>126 - 200	 >125<br>>200	Insulin requirements or hyperosmolar coma
Blood Urea Nitrogen (BUN) mg/dL	23 - 26	27 - 31	>31	Requires dialysis
Creatinine - mg/dL	1.5 - 1.7	1.8 - 2.0	2.1 - 2.5	> 2.5 or requires dialysis
Calcium - hypocalcemia mg/dL	8.0 - 8.4	7.5 - 7.9	7.0 - 7.4	< 7.0
Calcium - hypercalcemia mg/dL	10.5 - 11.0	11.1 - 11.5	11.6 - 12.0	> 12.0
Magnesium - hypomagnesemia mg/dL	1.3 - 1.5	1.1 - 1.2	0.9 - 1.0	< 0.9
Phosphorous, hypophosphatemia mg/dL	2.3 - 2.5	2.0 - 2.2	1.6 - 1.9	< 1.6
CPK - mg/dL	1.25 - 1.5 x ULN	1.6 - 3.0 x ULN	3.1 –10 x ULN	> 10 x ULN
Albumin - Hypoalbuminemia g/dL	2.8 - 3.1	2.5 - 2.7	< 2.5	--
Total Protein - Hypoproteinemia g/dL	5.5 - 6.0	5.0 - 5.4	< 5.0	--
Alkaline phosphate - increase by factor	1.1 - 2.0 x ULN	2.1 - 3.0 x ULN	3.1 - 10 x ULN	> 10 x ULN
Liver Function Tests - ALT, AST increase by factor	1.1 - 2.5 x ULN	2.6 - 5.0 x ULN	5.1 - 10 x ULN	> 10 x ULN
Bilirubin - when accompanied by any increase in LFT increase by factor	1.1 - 1.25 x ULN	1.26 - 1.5 x ULN	1.51 - 1.75 x ULN	> 1.75 x ULN
Bilirubin - when LFT is normal; increase by factor	1.1 - 1.5 x ULN	1.6 - 2.0 x ULN	2.0 - 3.0 x ULN	> 3.0 x ULN
Cholesterol	201 - 210	211 - 225	> 226	---
Pancreatic enzymes - amylase, lipase	1.1 - 1.5 x ULN	1.6 - 2.0 x ULN	2.1 - 5.0 x ULN	> 5.0 x ULN
Other values (as defined according to applicable regulations)	No interference with activity	Some interference with activity not requiring medical intervention	Prevents daily activity and requires medical intervention	ER visit or hospitalization

* The laboratory values provided in the tables serve as guidelines and are dependent upon institutional normal parameters. Institutional normal reference ranges should be provided to demonstrate that they are appropriate.

[a] The clinical signs or symptoms associated with laboratory abnormalities might result in characterization of the laboratory abnormalities as Potentially Life Threatening (Grade 4). For example. a low sodium value that falls within a grade 3 parameter (125-129 mE/L) should be recorded as a grade 4 hyponatremia event if the subject had a new seizure associated with the low sodium value

ALT = alanine aminotransferase, AST = aspartate aminotransferase, decr. = decrease, ER = emergency room, LFT = liver function test, PT = prothrombin time, PTT = partial thromboplastin time, WBC = white blood cell count, ULN = upper limit of normal.

From [33].

A-Table 3: Vital sign grading scale

	**Mild (Grade 1)**	**Moderate (Grade 2)**	**Severe (Grade 3)**	**Potentially Life Threatening (Grade 4)**
Tachycardia [beats per minute]	101 – 115	116 – 130	> 130	ER visit or hospitalization for arrhythmia
Bradycardia [beats per minute][a]	50 – 54	45 – 49	< 45	ER visit or hospitalization for arrhythmia
Hypertension (systolic) [mm Hg]	141 – 150	151 – 155	> 155	ER visit or hospitalization for malignant hypertension
Hypertension (diastolic) [mm Hg]	91 – 95	96 – 100	> 100	ER visit or hospitalization for malignant hypertension
Hypotension (systolic) [mm Hg]	85 – 89	80 – 84	< 80	ER visit or hospitalization for hypotensive shock
Respiratory rate – breaths per minute	17 – 20	21 – 25	> 25	Intubation

[a] When resting heart rate is between 60 to 100 beats per minute. Use clinical judgement when characterizing bradycardia among some healthy subject populations (e.g., conditioned athletes).

From [33].

Appendix 2 Potential immune-mediated diseases

Gastrointestinal disorders:

- Celiac disease
- Crohn's disease
- Ulcerative colitis
- Ulcerative proctitis

Liver disorders:

- Autoimmune cholangitis
- Autoimmune hepatitis
- Primary biliary cirrhosis
- Primary sclerosing cholangitis

Metabolic diseases:

- Addison's disease
- Autoimmune thyroiditis (including Hashimoto thyroiditis)
- Diabetes mellitus type I
- Grave's or Basedow's disease

Musculoskeletal disorders:

- Antisynthetase syndrome
- Dermatomyositis
- Juvenile chronic arthritis (including Still's disease)
- Mixed connective tissue disorder
- Polymyalgia rheumatica
- Polymyositis
- Psoriatic arthropathy
- Relapsing polychondritis
- Rheumatoid arthritis
- Scleroderma, including diffuse systemic form and CREST syndrome
- Spondyloarthritis, including ankylosing spondylitis, reactive arthritis (Reiter's Syndrome) and undifferentiated spondyloarthritis
- Systemic lupus erythematosus
- Systemic sclerosis

Neuro-inflammatory disorders:

- Acute disseminated encephalomyelitis, including site specific variants (e.g., noninfectious encephalitis, encephalomyelitis, myelitis, myeloradiculomyelitis)
- Cranial nerve disorders, including paralyses/paresis (e.g., Bell's palsy)
- Guillain-Barré syndrome, including Miller Fisher syndrome and other variants
- Immune-mediated peripheral neuropathies, Parsonage-Turner syndrome and plexopathies, including chronic inflammatory demyelinating polyneuropathy, multifocal motor neuropathy, and polyneuropathies associated with monoclonal gammopathy
- Multiple sclerosis
- Narcolepsy
- Optic neuritis
- Transverse myelitis

Skin disorders:

- Alopecia areata
- Autoimmune bullous skin diseases, including pemphigus, pemphigoid and dermatitis herpetiformis
- Cutaneous lupus erythematosus
- Erythema nodosum
- Morphoea
- Lichen planus
- Psoriasis
- Sweet's syndrome
- Vitiligo

Vasculitides:

- Large vessels vasculitis including: giant cell arteritis such as Takayasu's arteritis and temporal arteritis
- Medium sized and/or small vessels vasculitis including: polyarteritis nodosa, Kawasaki's disease, microscopic polyangiitis, Wegener's granulomatosis, Churg-Strauss syndrome (allergic granulomatous angiitis), Buerger's disease thromboangiitis obliterans, necrotizing vasculitis and anti-neutrophil cytoplasmic antibody (ANCA) positive vasculitis (type unspecified), Henoch-Schonlein purpura, Behcet's syndrome, leukocytoclastic vasculitis

Others:

- Antiphospholipid syndrome

- Autoimmune hemolytic anemia
- Autoimmune glomerulonephritis (including IgA nephropathy, glomerulonephritis rapidly progressive, membranous glomerulonephritis, membranoproliferative glomerulonephritis, and mesangioproliferative glomerulonephritis)
- Autoimmune myocarditis/cardiomyopathy
- Autoimmune thrombocytopenia
- Goodpasture syndrome
- Idiopathic pulmonary fibrosis
- Pernicious anemia
- Raynaud's phenomenon
- Sarcoidosis
- Sjögren's syndrome
- Stevens-Johnson syndrome
- Uveitis

Appendix 3 Adverse events of special interest for SARS-CoV-2 Vaccines

Current List of AESIs related to SARS-CoV-2 vaccines based on Brighton Collaboration via CEPI's Safety Platform for Emergency vACcines (SPEAC) Project (as of March 2021):

Immunological disorders:

- Anaphylaxis
- Vasculitides
- Multisystem inflammatory syndrome, Kawasaki Syndrome
- Systemic anaphylaxis
- Rheumatoid arthritis

Symptomatic infection with target disease:

- Acute respiratory distress syndrome (ARDS)
- Cytokine-release syndrome
- Pneumonitis
- Symptomatic COVID-19

Cardiac disorders:

- Acute cardiovascular injury including:
  - Cardial microangiopathy
  - Heart failure and cardiogenic shock
  - Stress cardiomyopathy
  - Coronary artery disease
  - Arrhythmia
  - Myocarditis, pericarditis

Hematological disorders:

- Thrombocytopenia (defined as < 50K/µL)
- Vasculitis

Coagulation disorder:

- Sinus venosus thrombosis
- Deep vein thrombosis
- Pulmonary embolism
- Cerebrovascular stroke
- Thromboembolic limb ischemia
- Coagulopathy (bleeding diathesis)

Renal disorders:

- Acute kidney injury

Gastrointestinal disorders

- Acute liver injury*
- Acute pancreatitis

Neurological disorders:

- Generalized seizure (grand mal)
- Acute disseminated encephalomyelitis
- Anosmia, ageusia
- Aseptic (meningo-) encephalitis/ encephalomyelitis
- Idiopathic peripheral N. facialis palsy

Dermatologic disorder:

- Chilblain-like lesions
- Single organ cutaneous vasculitis
- Erythema multiforme

Other:

- Acute aseptic arthritis
- Rhabdomyolysis
- Serious local/systemic AR following immunization
- Subacute thyreoditis

* Defined as:
AST/ALT > 3-fold upper limit of normal
GGT/AP > 2-fold upper limit of normal

ALT = alanine aminotransferase, AP = alkaline phosphatase, AST = aspartate aminotransferase, GGT = gamma-glutamyl transferase.
